# Multi-Parameter Genomic Evolution of SARS-CoV-2 Across Ten US States During the Vaccination Era, 2020–2025

**DOI:** 10.3390/bioengineering13101136

**Published:** 2026-09-28

**Authors:** Som V. Thomas, Jing Chen, Dong Qian, Paul Spearman, Donglu Shi

**Affiliations:** 1 Department of Biomedical Engineering, University of Cincinnati, Cincinnati, OH 45267, USA; valicksm@mail.uc.edu; 2 Cincinnati Children’s Hospital Medical Center, Cincinnati, OH 45229, USA; jing.chen2@cchmc.org (J.C.); paul.spearman@cchmc.org (P.S.); 3 Department of Mechanical Engineering, The University of Texas at Dallas, Richardson, TX 75080, USA; dong.qian@utdallas.edu; 4 The Materials Science and Engineering Program, University of Cincinnati, Cincinnati, OH 45267, USA

**Keywords:** SARS-CoV-2, molecular evolution, dN/dS, positive selection, immune escape, spike glycoprotein, genomic surveillance, GISAID

## Abstract

SARS-CoV-2 has adapted continuously since 2019, yet no multi-parameter framework has characterized this trajectory across geographically distinct United States populations. We present a systematic 13-parameter analysis of 137,059 high-quality genomes from ten diverse US states across three phases: pre-vaccination (January–December 2020), early vaccination (January–June 2021), and sustained immune pressure (July 2021–December 2025), the latter dominated by the high-volume Delta period with an early-Omicron contribution. Approximately 5000 genomes were retrieved per state per phase from GISAID EpiCoV under stringent quality filters, and each phase dataset was partitioned into three subsets, giving 90 subsamples in total, to quantify subsampling stability. Parameters spanned genome-wide mutation rate, dN/dS and Ti/Tv, site-specific selection (HyPhy FUBAR), spike and ACE2 contact-residue profiling, insertion–deletion characterization, furin cleavage site evolution, N-linked glycosylation dynamics, lineage–mutation concordance, amino acid physicochemical shifts, and protein-wise dN/dS ranking and mutation load. Genome-wide mutation rate rose monotonically in all ten states, from 0.050% to 0.149%, and dN/dS rose from 0.611 to 1.103, crossing neutrality in nine of ten states by Phase 3. Amino acid mutation load increased 3.95-fold, from 9.12 to 36.03 substitutions per sequence, while Ti/Tv fell from 3.69 to 2.11. Spike position 142 was diversifying in all ten states in Phase 3, with positions 484, 452, and 478 recurring in 70, 60, and 60 percent of states. ORF8 followed a spike-then-collapse trajectory (dN/dS 3.43, 14.23, 1.01), consistent with progressive loss of function. The reproducibility of these effects across ten geographically distinct state populations establishes a state-level baseline for SARS-CoV-2 immune escape.

## 1. Introduction

### 1.1. Background and Epidemiological Context

Severe acute respiratory syndrome coronavirus 2 (SARS-CoV-2), first described in patients with pneumonia in Wuhan in December 2019 [1], was declared the causative agent of a global pandemic by the World Health Organization on 11 March 2020 [2]. The United States bore one of the heaviest national burdens, accumulating over 100 million confirmed infections and nearly 1.2 million deaths through 2025 [3]. SARS-CoV-2 is a positive-sense, single-stranded RNA virus with a genome of approximately 29,903 nucleotides encoding 29 proteins [4,5]. Its relatively low evolutionary (substitution) rate—estimated at 1–2 × 10^−3^ substitutions per site per year—is constrained by the proofreading activity of the non-structural protein 14 (NSP14) ExoN domain, which limits the intrinsic polymerase error rate [6,7]. Nevertheless, the unprecedented global scale of replication, estimated at trillions of viral particles per infected host [8], has generated an enormous population-genetic substrate upon which natural selection, genetic drift, and recombination collectively shape viral evolution.

The emergence of Variants of Concern (VOC) beginning with Alpha (B.1.1.7) in late 2020 and progressing through Beta, Gamma, Delta (B.1.617.2), and the highly divergent Omicron lineage (B.1.1.529) [9,10] demonstrated that SARS-CoV-2 possesses substantial evolutionary plasticity [11,12,13,14]. The introduction of mRNA vaccines in early 2021 fundamentally altered the host immune landscape, intensifying selective pressure toward immune escape and accelerating functional evolution across the spike glycoprotein and other proteins [15,16,17].

### 1.2. Rationale for State-Level Analysis

The ten US states selected—California, Texas, New York, Florida, Minnesota, Colorado, Massachusetts, Washington, Illinois, and North Carolina—collectively represent over 40% of the US population and span distinct geographic regions, demographic profiles, and public health policy environments. Prior studies have characterized SARS-CoV-2 evolution at global or country scales [16,18,19,20], but systematic multi-parameter comparative analysis across US states has received limited attention. The existing literature typically examines single parameters such as lineage prevalence [21], spike receptor-binding domain (RBD) mutations [17], or phylogeographic spread [22], without integrating molecular, functional, and epidemiological dimensions into a unified framework. This fragmentation limits understanding of how different evolutionary facets are coupled within distinct regional contexts.

This study is presented as a computational, multi-parameter engineering framework for systematic surveillance of viral genomic evolution, intended for a computational/quantitative-genomics and biomedical-engineering readership rather than as a single-hypothesis virological study. Accordingly, the manuscript is organized around the framework itself: each of the thirteen parameters is benchmarked against prior literature and then evaluated across states and phases, and the framework’s contributions are stated explicitly.

### 1.3. Comprehensive Literature Review of the 13 Analytical Parameters

#### 1.3.1. Parameter 1: Genome-Wide Mutation Rate—Prior Literature

The genome-wide metric reported here quantifies the mean number of nucleotide differences per sequence relative to the Wuhan-Hu-1 reference (NC_045512.2), normalized to genome length and expressed as a percentage, following the approach of Gupta et al. Because this quantity derives from consensus sequences sampled from circulating populations, it measures the accumulation and fixation of substitutions detectable in sampled viral populations rather than the intrinsic rate at which replication errors arise. It is therefore a substitution (evolutionary divergence) measure rather than a mutation rate in the strict polymerase-error sense, and it is not expressed in substitutions per site per year. The term “mutation rate” is retained for consistency with the figure labels and the source methodology, but should be read throughout in this normalized, population-level sense.

The genome-wide mutation rate of SARS-CoV-2 has been intensively investigated since the earliest days of the pandemic. Early phylodynamic analyses [23] converged on an evolutionary rate of about 1.1 × 10^−3^ substitutions per site per year during 2020, broadly consistent with other betacoronaviruses and significantly slower than influenza A. Real-time genomic surveillance platforms such as Nextstrain [24] made rates of this kind trackable at scale; across the 29,903-nucleotide genome, the rate above corresponds to approximately 33 substitutions per genome per year. The landmark study by Gupta et al. [18] characterized genome-wide mutation rates across seven countries across all three pandemic phases, demonstrating a statistically significant progressive increase in per-genome mutation rates from pre-vaccination to the Omicron era, interpreted as reflecting both increased viral population size and positive selection for immune escape mutations. van Dorp et al. [25] performed a comprehensive analysis of early 2020 genomes and identified that most circulating mutations were selectively neutral or mildly deleterious, consistent with the dominance of purifying selection during early host adaptation. The most dramatic acceleration was associated with Omicron (B.1.1.529), which harbored over 50 genome-wide substitutions, suggesting an accelerated evolutionary origin possibly involving prolonged infection of an immunocompromised host [14]. Subsequent Omicron subvariant diversification through BA.2, BA.4/5, BQ.1, XBB, and JN.1 has maintained elevated substitution rates through 2023–2025, with convergent mutations appearing independently across multiple sublineages [26,27,28]. Turakhia et al. [29] introduced UShER, a maximum-parsimony method for placing new samples onto an existing phylogeny, which made pandemic-scale tree building and real-time lineage tracking tractable.

Recent experimental work has significantly advanced understanding of SARS-CoV-2 mutation mechanisms. Haddox et al. [30] analyzed ~16 million GISAID sequences and demonstrated that mutation rates are highly variable between individual genomic sites, influenced by sequence context, genomic region, and RNA secondary structure; synonymous C→T counts are substantially higher at sites predicted to be unpaired than at paired sites, providing a molecular basis for the observed C→T transition bias. Amicone et al. [31] estimated a spontaneous mutation rate of 1.3 × 10^−6^ ± 0.2 × 10^−6^ per base per infection cycle from experimental evolution of two SARS-CoV-2 lineages over fifteen passages in Vero cells, and report that the spike gene accumulated mutations at approximately five times the genomic average. Ma et al. [32] recently emphasized that SARS-CoV-2 continues to evolve under shifting immune pressures, with mutation accumulation patterns reflecting the interplay between viral fitness and population-level immunity even five years into the pandemic.

#### 1.3.2. Parameter 2: Nonsynonymous-to-Synonymous Substitution Ratio (dN/dS)—Prior Literature

The ratio of nonsynonymous (dN)-to-synonymous (dS) substitution rates provides a foundational measure of the direction and magnitude of natural selection. A dN/dS < 1 indicates net purifying selection; dN/dS = 1 indicates neutral evolution; dN/dS > 1 indicates positive selection. Gupta et al. [18] reported dN/dS rising from approximately 0.48 in Phase 1 to 0.99 in Phase 2 before easing to 0.90 in Phase 3 (Table 1b), a near doubling that approaches but does not cross the neutral threshold, indicating progressive relaxation of purifying selection without a net shift to positive selection at the genome level. Kryazhimskiy and Plotkin [33] showed that dN/dS behaves differently for sequences sampled from a single population, whose differences are segregating polymorphisms, than for distantly diverged lineages, whose differences are substitutions fixed independently: within a population the ratio is relatively insensitive to the selection coefficient, the relationship between the two is not monotonic, and the conventional dN/dS > 1 signature of adaptive evolution does not carry its usual meaning. Every sequence analysed here is a segregating polymorphism drawn from one viral population, so that regime applies; the genome-wide ratio is therefore read as a comparative trajectory measured identically across phases and states, and its crossing of unity as a shift in the balance of constraint rather than as a demonstration of adaptation. Gene-wise analyses of early SARS-CoV-2 genomes report strong purifying selection across ORF1ab, ORF3a, and N, with weaker constraints in the spike receptor-binding domain [18]. Yang et al. [34] developed the widely used PAML codon substitution models underpinning dN/dS analysis. Murrell et al. [35] introduced the Mixed Effects Model of Evolution (MEME) for detecting episodic diversifying selection at individual sites.

**Table 1:** **(a) Genome-wide Mutation Rate (%).** Statistical significance: *** *p* < 0.001; Welch’s unpaired *t*-test on per-sequence values within each phase; see Section 2.8. All *p*-values for Phase 1 vs. Phase 3 comparisons across the 10 US states are below 10^−10^, and below 10^−300^ in most; these are extremely small because the test is applied across thousands of individual sequences that share lineage ancestry and are therefore not independent observations, and they should be read as indicating the consistency and direction of the phase differences rather than as calibrated significance (see Section 2.8). **(b): Genome-wide dN/dS Ratio.** Statistical significance: *** *p* < 0.001; ns = not significant (Welch’s unpaired *t*-test on per-sequence values within each phase; see Section 2.8). All *p*-values for Phase 1 vs. Phase 3 comparisons across the 10 US states are below 10^−10^, and below 10^−300^ in most; these are extremely small because the test is applied across thousands of individual sequences that share lineage ancestry and are therefore not independent observations, and they should be read as indicating the consistency and direction of the phase differences rather than as calibrated significance (see Section 2.8). Colorado P(1v2) = 0.403 (ns) and North Carolina P(1v2) = 0.060 (ns) reflect limited Phase 1 sample sizes. **(c): Transition/Transversion (Ti/Tv) Ratio.** Statistical significance: *** *p* < 0.001; Welch’s unpaired *t*-test on per-sequence values within each phase; see Section 2.8. All *p*-values for Phase 1 vs. Phase 3 comparisons across the 10 US states are below 10^−10^, and below 10^−300^ in most; these are extremely small because the test is applied across thousands of individual sequences that share lineage ancestry and are therefore not independent observations, and they should be read as indicating the consistency and direction of the phase differences rather than as calibrated significance (see Section 2.8).

(**a**)									
**Location**	**Nᴵ**	**Phase 1**	**Phase 2**	**Phase 3**	**Fold (P3/P1)**	***p* (1 vs. 2)**	***p* (2 vs. 3)**	***p* (1 vs. 3)**	**Sig.**
**California**	**5000**	0.0601 ± 0.0005	0.0994 ± 0.0006	0.1436 ± 0.0006	**2.39×**	<10^−300^	<10^−300^	<10^−300^	***
**Colorado**	**2950**	0.0546 ± 0.0008	0.1172 ± 0.0007	0.1458 ± 0.0005	**2.67×**	<10^−300^	<10^−300^	<10^−300^	***
**Florida**	**2743**	0.0473 ± 0.0008	0.1144 ± 0.0006	0.1327 ± 0.0003	**2.81×**	<10^−300^	<10^−300^	<10^−300^	***
**Illinois**	**3749**	0.0460 ± 0.0006	0.1124 ± 0.0005	0.1529 ± 0.0010	**3.33×**	<10^−300^	<10^−300^	<10^−300^	***
**Massachusetts**	**1928**	0.0437 ± 0.0010	0.1059 ± 0.0004	0.1434 ± 0.0004	**3.28×**	<10^−300^	<10^−300^	<10^−300^	***
**Minnesota**	**5000**	0.0519 ± 0.0004	0.1126 ± 0.0004	0.1400 ± 0.0002	**2.70×**	<10^−300^	<10^−300^	<10^−300^	***
**New York**	**5000**	0.0497 ± 0.0002	0.0947 ± 0.0008	0.1777 ± 0.0004	**3.58×**	<10^−300^	<10^−300^	<10^−300^	***
**N. Carolina**	**689**	0.0526 ± 0.0007	0.1025 ± 0.0001	0.1465 ± 0.0012	**2.79×**	<10^−300^	<10^−300^	<10^−300^	***
**Texas**	**5000**	0.0553 ± 0.0000	0.1042 ± 0.0006	0.1484 ± 0.0004	**2.68×**	<10^−300^	<10^−300^	<10^−300^	***
**Washington**	**5000**	0.0396 ± 0.0005	0.1132 ± 0.0002	0.1564 ± 0.0014	**3.95×**	<10^−300^	<10^−300^	<10^−300^	***
**Average**		**0.0501 ± 0.0006**	**0.1076 ± 0.0005**	**0.1488 ± 0.0006**	**2.97×**				
**Gupta et al. [18]**	7 ctry	0.04 ± 0.02	0.17 ± 0.05	0.28 ± 0.01					
(**b**)									
**Location**	**Nᴵ**	**Phase 1**	**Phase 2**	**Phase 3**	**Fold (P3/P1)**		***p*** **(1 vs. 3)**		**Sig.**
**California**	**5000**	0.50 ± 0.00	0.61 ± 0.01	1.18 ± 0.00	**2.37×**		<10^−300^		***
**Colorado**	**2950**	0.69 ± 0.02	0.72 ± 0.00	1.10 ± 0.01	**1.59×**		<10^−100^		***
**Florida**	**2743**	0.59 ± 0.00	0.73 ± 0.00	1.20 ± 0.00	**2.03×**		<10^−300^		***
**Illinois**	**3749**	0.66 ± 0.01	0.70 ± 0.01	1.06 ± 0.01	**1.61×**		<10^−100^		***
**Massachusetts**	**1928**	0.61 ± 0.01	0.73 ± 0.00	1.17 ± 0.01	**1.92×**		<10^−300^		***
**Minnesota**	**5000**	0.65 ± 0.01	0.71 ± 0.01	1.09 ± 0.01	**1.68×**		<10^−300^		***
**New York**	**5000**	0.61 ± 0.01	0.75 ± 0.00	0.95 ± 0.00	**1.56×**		<10^−100^		***
**N. Carolina**	**689**	0.63 ± 0.05	0.72 ± 0.01	1.09 ± 0.00	**1.74×**		<10^−10^		***
**Texas**	**5000**	0.64 ± 0.00	0.69 ± 0.01	1.10 ± 0.00	**1.71×**		<10^−100^		***
**Washington**	**5000**	0.53 ± 0.00	0.64 ± 0.00	1.09 ± 0.01	**2.05×**		<10^−300^		***
**Average**		**0.61 ± 0.01**	**0.70 ± 0.00**	**1.10 ± 0.01**	**1.80×**				
**Gupta et al. [18]**	7 ctry	0.48 ± 0.06	0.99 ± 0.11	0.90 ± 0.05					
(**c**)									
**Location**	**Nᴵ**	**Phase 1**	**Phase 2**	**Phase 3**	**Fold (P3/P1)**	***p*** **(1 vs. 2)**	***p*** **(2 vs. 3)**	***p*** **(1 vs. 3)**	**Sig.**
**California**	**5000**	3.18 ± 0.02	2.15 ± 0.03	2.09 ± 0.01	**0.66×**	<10^−100^	<10^−10^	<10^−300^	***
**Colorado**	**2950**	3.40 ± 0.04	1.89 ± 0.01	2.13 ± 0.01	**0.63×**	<10^−300^	<0.001	<10^−300^	***
**Florida**	**2743**	3.99 ± 0.11	2.06 ± 0.00	2.08 ± 0.01	**0.52×**	<10^−300^	<10^−10^	<10^−300^	***
**Illinois**	**3749**	4.12 ± 0.07	2.09 ± 0.01	2.12 ± 0.01	**0.51×**	<10^−300^	<10^−10^	<10^−300^	***
**Massachusetts**	**1928**	3.39 ± 0.04	2.15 ± 0.03	2.09 ± 0.00	**0.62×**	<10^−10^	<10^−100^	<10^−100^	***
**Minnesota**	**5000**	3.68 ± 0.03	1.93 ± 0.01	2.12 ± 0.01	**0.58×**	<10^−300^	<10^−10^	<10^−300^	***
**New York**	**5000**	4.09 ± 0.07	2.82 ± 0.07	2.08 ± 0.00	**0.51×**	<10^−10^	<10^−300^	<10^−300^	***
**N. Carolina**	**689**	3.65 ± 0.09	2.48 ± 0.02	2.11 ± 0.01	**0.58×**	<10^−10^	<10^−100^	<10^−10^	***
**Texas**	**5000**	3.78 ± 0.03	2.18 ± 0.03	2.06 ± 0.01	**0.54×**	<10^−100^	<10^−100^	<10^−300^	***
**Washington**	**5000**	3.57 ± 0.07	2.02 ± 0.00	2.17 ± 0.01	**0.61×**	<10^−300^	<10^−10^	<10^−300^	***
**Average**		**3.69 ± 0.06**	**2.18 ± 0.02**	**2.11 ± 0.01**	**0.57×**				
**Gupta et al. [18]**	7 ctry	3.77 ± 1.10	2.00 ± 0.12	2.15 ± 0.05					

dN/dS > 1.0 = positive (diversifying) selection; dN/dS < 1.0 = purifying (stabilizing) selection; dN/dS = 1.0 = neutral evolution. Dashed line in Figure 1b indicates neutrality threshold. Ti/Tv > 1.0 indicates transition bias from APOBEC3-mediated cytidine deamination. Decreasing Ti/Tv reflects broadening of the mutational spectrum under immune-driven selection pressure.

**Figure 1:**
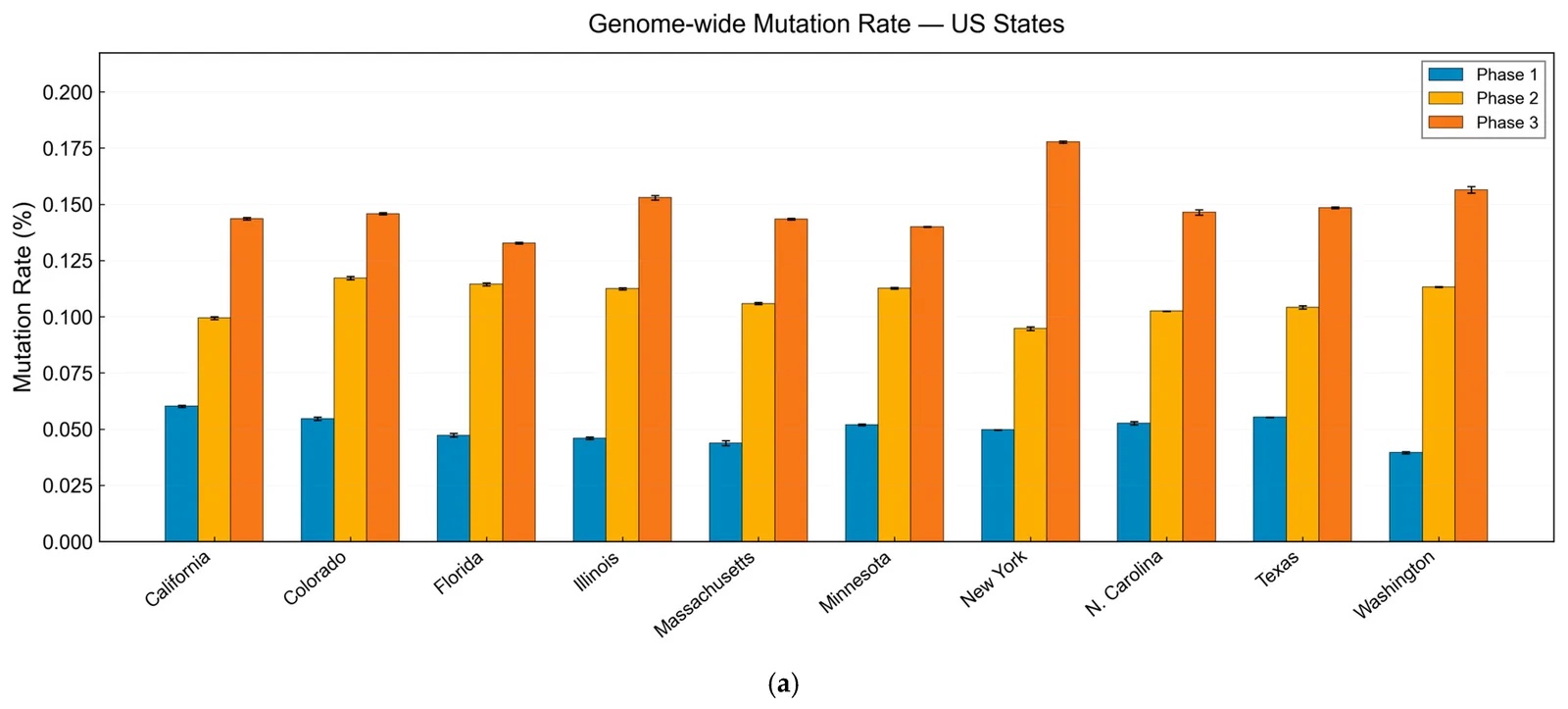

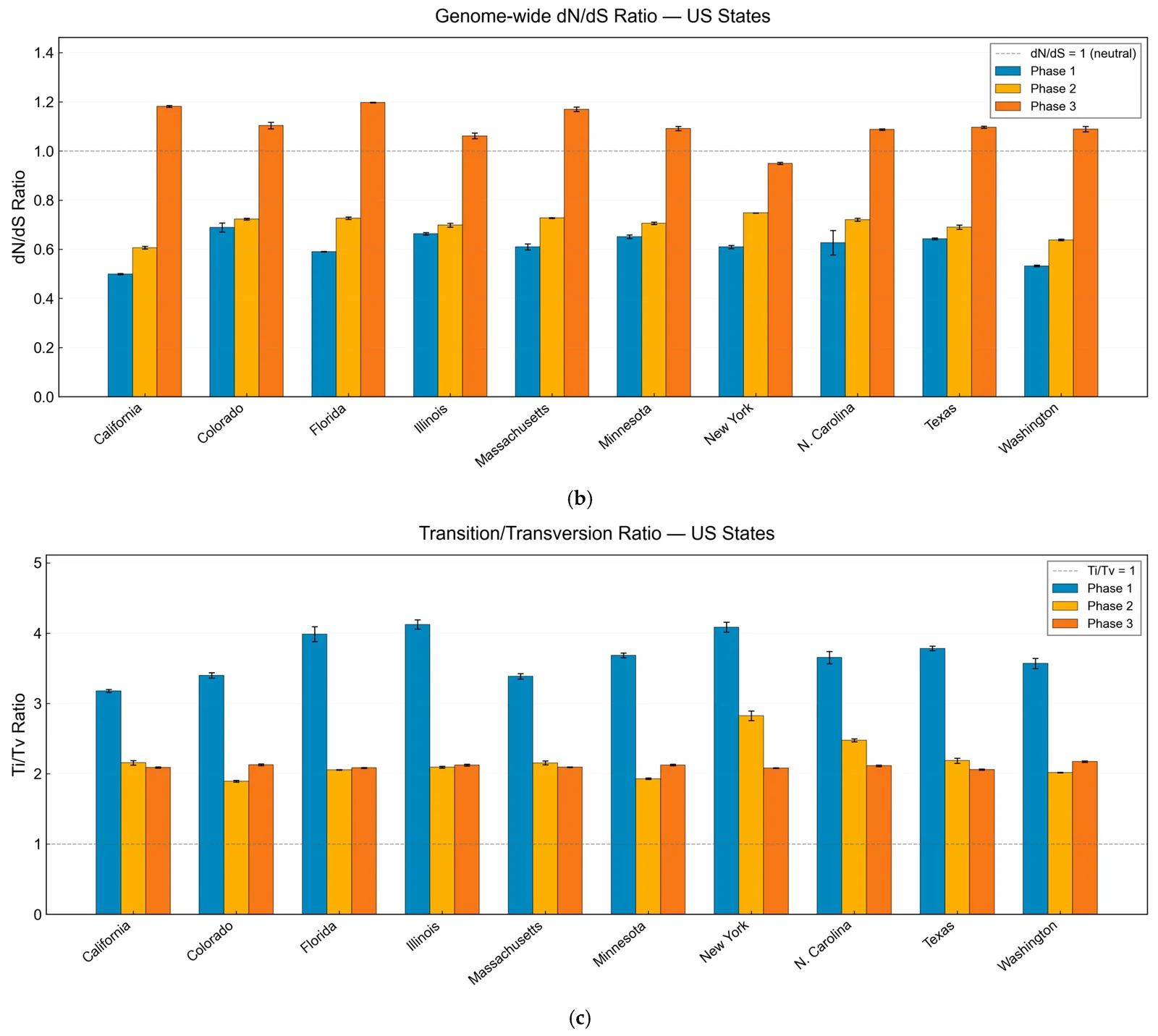
(**a**) Genome-wide mutation rate (%) across three temporal phases for each US state. Bars = mean from triplicate analysis; error bars = ± SD. Okabe-Ito colorblind-friendly palette. (**b**) Genome-wide dN/dS ratio for each US state. Dashed line = neutrality (dN/dS = 1.0). Nine of ten states crossed in Phase 3; exception: New York (0.95). (**c**) Ti/Tv ratio for each US state. Phase 1→Phase 2 decline reflects APOBEC C→T bias shifting to immune-driven diversification.

Maiti [36] performed temporal estimation of ω (dN/dS) across the whole genome and all 29 SARS-CoV-2 genes of Alpha, Delta, and Omicron strains, demonstrating that the virus originally emerged with strong purifying selection (ω~0.04) and progressively shifted toward neutral/positive selection as it adapted to human immune systems—providing the evolutionary trajectory framework that this study extends through 2025. Liu [37] recently documented that by 2025, the overall fitness effects of nonsynonymous mutations had become increasingly negative in certain genomic regions, suggesting that SARS-CoV-2 may be approaching adaptive constraints even as diversifying selection persists genome-wide. Ghafari et al. [38] revealed through population-based UK surveillance that within-host evolution in persistent infections is primarily driven by nonsynonymous mutations, with bursts of adaptive evolution at lineage transition points showing dramatically elevated dN/dS ratios compared to within-lineage neutral accumulation.

#### 1.3.3. Parameter 3: Transition-to-Transversion Ratio (Ti/Tv)—Prior Literature

The Ti/Tv ratio captures the mutational spectrum of the virus, distinguishing chemically conservative transitions (A↔G; C↔T) from transversions (purines↔pyrimidines). For SARS-CoV-2, Gupta et al. [18] report an early Ti/Tv ratio of approximately 3.77 (their Table 1), consistent with C→T transitions mediated by APOBEC3 family cytidine deaminases acting on single-stranded RNA [39]. Simmonds et al. [40,41] demonstrated that SARS-CoV-2 shows APOBEC3-mediated TC→TT hypermutation at characteristic trinucleotide contexts. Both studies predate the Omicron era and are cited here for the hypermutation mechanism rather than for lineage-specific patterns. Gupta et al. [18] reported a significant decrease in Ti/Tv from approximately 3.77 in Phase 1 to 2.00 in Phase 2, with a slight recovery to 2.15 in Phase 3 (Table 1c), suggesting the mutational spectrum diversifies under immune pressure before settling at a new equilibrium as transversion rates rise and purifying selection relaxes. Nei and Kumar [42] predict that sustained positive selection broadens the spectrum of substitutions reaching observable frequency, because escape can require amino acid changes accessible only by transversion; the underlying mutational process is unchanged, and it is the fixation spectrum that widens. Omicron carries an unusually broad substitution spectrum relative to earlier lineages [43], consistent with the widening expected under strong positive selection.

Simmonds & Ansari [44] confirmed in a landmark 2024 study that C→U transition biases remain rampant even four years into the pandemic, driving a net loss of C bases and accumulation of U bases at approximately 0.2–0.25% per decade. Critically, most C→U substitutions occurred in the absence of the preferred upstream-base context or targeting of unpaired RNA bases previously associated with APOBEC3A, suggesting additional cytidine deamination mechanisms beyond canonical APOBEC editing. Di Giorgio et al. [45] provided direct evidence of both APOBEC-mediated C→U and ADAR-mediated A→G editing in SARS-CoV-2 transcriptomes from patient bronchoalveolar lavage samples, confirming the host-driven origin of the transition bias observed in genomic surveillance data. Azgari et al. [46] demonstrated through phylogenetic mutation profiling that the heterogeneous mutation patterns of SARS-CoV-2 are primarily reflections of host antiviral mechanisms achieved through APOBEC, ADAR, and ZAP proteins, with the second most prevalent substitution (G→T) likely associated with reactive oxygen species produced during APOBEC3A-mediated DNA damage response.

#### 1.3.4. Parameter 4: Site-Specific Positive Selection (HyPhy FUBAR)—Prior Literature

Site-specific selection analysis identifies individual codons experiencing pervasive positive selection. The Fast Unconstrained Bayesian Approximation (FUBAR) method, developed by Murrell et al. [47] and implemented in HyPhy [48,49], uses a Bayesian framework with a Dirichlet process prior to estimate site-specific dN and dS rates, classifying sites as experiencing pervasive positive selection (dN/dS > 1, posterior probability > 0.9) or purifying selection. Early application of FUBAR to SARS-CoV-2 by Martin et al. [50] applied selection analysis to identify clusters of unusual mutational change in the Omicron BA.1 lineage. Carabelli et al. [22] applied FUBAR within a comprehensive review and identified convergent positive selection at positions 484, 452, 478, and 501 across independent VOC lineages—a hallmark of immune selection pressure on shared antigenic epitopes. Selection analyses spanning the pandemic timeline indicate that positive selection in the spike RBD intensified progressively, with immune-escape positions showing increasingly strong support for positive selection [22]. Gayvert et al. [19] analyzed over 13 million deposited genomes, of which 820,056 met their quality filters and formed the analysis population, and applied FUBAR to down-sampled subsets drawn from it. They report that the genome was strongly conserved during the first year of the pandemic, with only 3.6% of receptor-binding domain sites under diversifying pressure, and identify elevated diversifying selection in N, E, ORF3a, ORF8, and ORF10.

Oliver et al. [51] reported decreasing compositional heterogeneity in later SARS-CoV-2 genomes, consistent with genome-wide selective sweeps reducing nucleotide diversity at positively selected sites while maintaining variation at neutral positions. This compositional stabilization in Phase 3 genomes provides independent evidence corroborating the FUBAR-detected pattern of pervasive positive selection at immune-relevant sites during the Omicron era. Essabbar et al. [52] analyzed 16,575 complete sequences across 214 countries through October 2023, identifying 21,580 total mutations classified into transient and persistent categories, with persistent mutations showing steadily increasing frequencies consistent with ongoing positive selection at functionally important sites.

#### 1.3.5. Parameter 5: Spike Protein Mutation Profile—Prior Literature

The spike glycoprotein, encoded by a 3822-nucleotide ORF, is the primary determinant of host cell entry and the principal neutralizing antibody target [53,54]. Its prefusion trimeric conformation was resolved by cryo-electron microscopy early in the pandemic [55], providing the structural reference against which subsequent substitutions are interpreted. The D614G substitution, documented by Korber et al. [56], became the first major globally fixed mutation in SARS-CoV-2, with mechanistic studies demonstrating enhanced ACE2 binding affinity and improved upper-respiratory-tract replication [54,57,58]. Subsequent landmark mutations include N501Y (Alpha, Beta, Gamma) enhancing ACE2 binding; E484K (Beta, Gamma) disrupting class II antibody binding; L452R (Delta, BA.4/5) disrupting class III antibody binding; P681H/R (Alpha/Delta) facilitating furin cleavage; and the extensive Omicron BA.1 constellation (G339D, S371L, N440K, E484A, Q493R, N501Y, and 27 additional changes) collectively producing a massively altered antigenic surface [14,17]. Harvey et al. [43] provided a comprehensive framework demonstrating that successive variants progressively targeted distinct antibody epitope classes on the RBD surface. Wang et al. [27] identified positions 346, 444, 452, 455, 456, 484, 486, and 490 as preferentially targeted by convergent mutations across independent Omicron subvariant lineages. Ito et al. [59] characterized convergent evolution among Omicron subvariants leading to the emergence of BQ.1.1, showing that antigenically advantageous substitutions arose repeatedly on independent genetic backgrounds. The functional importance of spike mutations has also been validated through experimental inactivation studies, with Thomas et al. [60] demonstrating effective SARS-CoV-2 pseudovirus inactivation via reactive oxygen species generated by cold atmospheric plasma treatment, indicating that spike structural integrity is required for viral infectivity; the reactive-species mechanisms underlying this and related antiviral applications are reviewed in [61].

The convergent evolution of spike mutations has accelerated in the post-Omicron era. Multiple independent Omicron sublineages (XBB, EG.5, HV.1, JN.1, KP.2, KP.3) have independently acquired mutations at overlapping RBD positions, demonstrating that immune selection pressure drives predictable evolutionary trajectories across geographically distinct viral populations [26]. Deep mutational scanning studies continue to refine understanding of the fitness landscape, revealing that epistatic interactions between spike mutations create non-additive effects on ACE2 binding and antibody escape that cannot be predicted from single-mutation analysis alone.

#### 1.3.6. Parameter 6: Insertion–Deletion (Indel) Analysis—Prior Literature

Insertions and deletions (indels) in the SARS-CoV-2 genome represent a biologically consequential dimension of viral evolution that can alter protein secondary structure and reshape protein surface topology. McCarthy et al. [62] documented recurrent in-frame deletions clustering in the spike NTD antigenic supersite. Meng et al. [63] characterized a recurrent 69–70 deletion in the spike protein present in Alpha and independently in multiple immunocompromised patient-derived sequences, demonstrating convergent evolutionary response to specific antibody pressures. McCarthy et al. [62] showed that NTD deletions in the recurrent deletion regions (RDR2, positions 139–146; RDR4, positions 242–248) are positively selected for antibody escape and appeared independently in multiple VOC lineages. The Omicron lineage introduced a novel 3-nucleotide insertion at position 214 (ins214EPE) absent from all prior VOCs [14]. Indel content differs markedly between early and late lineages, with Omicron subvariants carrying a larger complement of characterized deletions than Phase 1 ancestral sequences [14,62]. Indels are not among the parameters examined by [18], whose analysis covers mutation rate, dN/dS, Ti/Tv, GC content and protein-wise rates; the phase-resolved indel analysis reported here is therefore introduced rather than extended from that work.

The Omicron-era expansion of indel diversity continued through 2024–2025, with JN.1 and KP.2 subvariants introducing novel insertions in the spike NTD and deletions in ORF8 that alter protein structure and potentially modulate innate immune recognition. These ongoing indel accumulations extend the Phase 3 indel density trends reported by earlier studies into the contemporary surveillance period covered by this analysis.

#### 1.3.7. Parameter 7: ACE2 Receptor-Binding Domain Contact Residue Tracking—Prior Literature

The angiotensin-converting enzyme 2 (ACE2) receptor serves as the primary SARS-CoV-2 entry receptor, with the spike receptor-binding motif forming an extensive interface with ACE2 resolved at 2.45 Å by Lan et al. [64]. This study tracks a panel of 17 receptor-binding motif positions selected to combine structurally defined ACE2 contacts with the immune-escape positions mapped by Greaney et al. [65] and McCarthy et al. [62]: K417, G446, Y449, Y453, L455, F456, A475, S477, E484, F490, Q493, G496, Q498, T500, N501, G502, and Y505. The panel is therefore not a complete enumeration of the structural interface; three canonical ACE2 contacts (F486, N487, Y489) fall outside it, while S477, E484, and F490 are included for their established roles in antibody escape. Positional burdens reported for this parameter should be read as applying to this defined panel rather than to the entire ACE2 interface. Deep mutational scanning by Starr et al. [66] comprehensively mapped fitness consequences of substitutions across the SARS-CoV-2 RBD, identifying that a subset of contact residues tolerates substitutions that simultaneously increase ACE2 affinity and reduce antibody neutralization efficiency. Greaney et al. [65] demonstrated using antibody escape mapping that mutations at ACE2 contact positions frequently escape class I and class II antibody binding while preserving ACE2 interaction. Carabelli et al. [22] found that the fraction of the 18 canonical contact residues defined in that study mutated from the ancestral sequence increased from approximately 2% in Phase 1 to over 60% in Omicron BA.1 and successors, demonstrating progressive remodeling of the ACE2 interface under simultaneous immune and receptor pressure. Yan et al. [67] determined the structure of the SARS-CoV-2 receptor-binding domain in complex with full-length human ACE2, defining the contact interface against which subsequent variant substitutions are assessed.

The progressive remodeling of ACE2 contact residues through 2025 produced subvariants (KP.2, KP.3, LB.1) with binding geometries increasingly divergent from the original Wuhan-Hu-1 interface, yet maintaining or enhancing receptor affinity through compensatory epistatic interactions. This ongoing contact residue evolution represents a continuous optimization process driven by the dual selective pressure of antibody escape and receptor binding maintenance, the escape component of which Liu et al. [68] characterized for the Omicron variant.

#### 1.3.8. Parameter 8: Furin Cleavage Site Evolution—Prior Literature

The polybasic furin cleavage site at the SARS-CoV-2 spike S1/S2 junction (positions 679–685: NSPRRAR, cleaved after R685) is a defining feature not present in closely related sarbecoviruses [69]. Furin cleaves the spike protein during viral egress, pre-activating it for enhanced membrane fusion and broadening cellular tropism [70,71]. The P681H mutation in Alpha was shown by Lubinski et al. [72] and Peacock et al. [73] to enhance furin cleavage efficiency relative to Wuhan-Hu-1, contributing to increased Alpha transmissibility. The independently arising P681R substitution in Delta produced even greater enhancement, as quantified by Saito et al. [74] using fluorogenic peptide cleavage assays. Both P681H and P681R arose convergently, providing strong evidence of positive selection for furin cleavage optimization. Meng et al. [75] demonstrated that Omicron BA.1’s N679K/P681H combination impairs TMPRSS2-mediated entry, shifting tropism toward the upper respiratory tract and endosomal pathway, proposed as a mechanistic basis for Omicron’s altered pathogenicity. Gupta et al. [18] reported increasing substitution frequency at furin site positions 679–685 from Phase 1 through Phase 3.

By Phase 3 (2021–2025), the furin cleavage site had undergone a complete evolutionary transition: P681H (Alpha) gave way to P681R (Delta), which was subsequently replaced by the Omicron N679K/P681H combination. This temporal succession reflects adaptive remodeling of the cleavage site for shifting tropism rather than monotonic optimization of cleavage efficiency: Delta’s P681R produced the greatest cleavage enhancement, whereas Omicron’s N679K/P681H trades cleavage efficiency for altered protease usage and upper-respiratory tropism, so each major lineage transition remodels the site in the direction set by the prevailing selective niche rather than by cleavage efficiency alone.

#### 1.3.9. Parameter 9: N-Linked Glycosylation Site Analysis—Prior Literature

N-linked glycosylation (attachment of oligosaccharide chains to NxS/T sequons, x ≠ proline) modulates SARS-CoV-2 protein folding, stability, immune recognition, and ACE2 binding geometry [53,76]. The spike protein harbors 22 N-linked glycosylation sites confirmed by mass spectrometry [53], forming a dense glycan shield that masks epitopes from antibody recognition [77]. Grant et al. [78] reported that glycans contribute approximately 17% of the trimer molecular weight yet shield approximately 40% of the protein surface, with the receptor-binding domain a notable exception. Casalino et al. [79] used molecular dynamics simulation to show that the N165 and N234 glycans directly modulate RBD conformational dynamics, with removal of either reducing ACE2 binding. Ives et al. [80] extended this analysis to the N343 glycan, showing through molecular dynamics that its loss triggers a consistent conformational change in the RBD, with the downstream effect on receptor-binding motif conformation differing between ancestral, Delta, and Omicron (BA.1, BA.2.86) backgrounds. Beyond shielding, the spike glycans also serve as attachment ligands. Lempp et al. [81] showed that C-type lectin receptors bind spike glycans and enhance infection of ACE2-expressing cells, and that antibodies which do not neutralize ACE2-mediated entry can nonetheless block this lectin-dependent enhancement, so changes to the sequon complement carry consequences for attachment as well as for epitope masking. Site-specific mapping across variants of concern by Shajahan et al. [82] subsequently showed that Alpha and Delta largely preserved the ancestral glycosylation architecture while Omicron introduced more substantial remodeling, and Wang et al. [83] documented the resulting alterations in glycan coverage over critical neutralization epitopes across Omicron subvariants. Gupta et al. [18] reported significant changes in the number of NxS/T glycosylation sequons across their three temporal phases.

Through 2025, Omicron subvariants have maintained the core glycan shield architecture while introducing subtle modifications at positions adjacent to key neutralization epitopes. These glycan shield dynamics represent an underappreciated dimension of immune evasion: rather than wholesale glycan addition or removal, the virus fine-tunes glycan positioning to modulate antibody accessibility at specific epitope surfaces while preserving the overall structural integrity required for spike protein folding and ACE2 engagement.

#### 1.3.10. Parameter 10: Lineage-Mutation Temporal Dynamics—Prior Literature

The Pango lineage nomenclature system, developed by Rambaut et al. [11] and implemented in the pangolin (Phylogenetic Assignment of Named Global Outbreak LINeages) assignment software, assigns each SARS-CoV-2 genome a hierarchical lineage designation reflecting phylogenetic position. The temporal succession of dominant SARS-CoV-2 lineages in the United States followed a pattern of competitive selective sweeps: ancestral B.1 dominated through 2020; Alpha emerged to dominance in early 2021; Delta displaced Alpha in mid-2021; Omicron BA.1 displaced Delta with unprecedented rapidity in December 2021–January 2022; and successive Omicron subvariants engaged in ongoing competitive dynamics through 2025 [3,84], a process in which antigenically advantageous substitutions arose repeatedly on independent genetic backgrounds [59]. Lineage-mutation concordance analysis, which validates that sequences assigned a given lineage harbor their lineage-defining mutations, provides an essential quality control framework [11,18]. Shannon entropy of lineage diversity (H = −Σ(p_i_ × log_2_p_i_)) provides a useful scalar measure of evolutionary diversification that increases from Phase 1 (low diversity, ancestral B.1 dominance) through Phase 3 (high diversity, multiple co-circulating lineages), correlating with positive selection intensity measured by site-specific FUBAR analysis.

The lineage diversity landscape has continued to evolve through 2025, with the JN.1 to KP.2/KP.3 transition representing the most recent competitive selective sweep. Shannon entropy analysis reveals that Phase 3 lineage diversity has remained consistently elevated relative to Phases 1 and 2, with multiple co-circulating Omicron subvariants maintaining population-level genetic heterogeneity even as individual sublineages undergo rapid competitive displacement.

#### 1.3.11. Parameter 11: Amino Acid Physicochemical Property Changes—Prior Literature

The Grantham distance scale [85], which quantifies physicochemical dissimilarity of amino acid substitutions using a composite score incorporating polarity, composition, and molecular volume, provides a standardized framework for classifying substitutions from conservative (Grantham < 50) to radical (Grantham > 100). Cao et al. [17] demonstrated that Omicron BA.1 spike substitutions produce a dramatic net change in RBD surface charge and polarity relative to Wuhan-Hu-1, with Q493R, Q498R, and N501Y together substantially increasing the positive electrostatic potential of the receptor-binding motif. Gupta et al. [18] reported that the proportion of radical (Grantham > 100) substitutions in spike increased from approximately 18% in Phase 1 to 43% in Phase 3, indicating a progressive shift toward physicochemically disruptive substitutions under intensifying immune selection. Consistent with this, spike substitutions accumulate disproportionately at exposed surface residues and are biased toward radical rather than conservative physicochemical changes, a pattern consistent with immune evasion rather than pure functional optimization. Nei and Kumar [42] predict that immune-driven directional selection should favor physicochemically radical substitutions at antibody contact surfaces to maximize antigenic distance from the original epitope.

The progressive increase in radical amino acid substitutions observed through 2025 has been particularly pronounced at antibody contact surfaces in the spike RBD, where charge-altering mutations (e.g., Q493R→Q493 reversion, followed by novel substitutions at positions 455, 456, and 478) have repeatedly remodeled the electrostatic landscape of key neutralization epitopes. This pattern of iterative physicochemical disruption at immune-relevant surfaces, combined with conservation at ACE2 contact interfaces, provides molecular evidence for the dual-pressure model of SARS-CoV-2 spike evolution.

#### 1.3.12. Parameter 12: Protein-Wise dN/dS Ranking—Prior Literature

Calculating dN/dS independently for each of the 25 individually analyzable SARS-CoV-2 proteins and ranking them from most to least positively selected provides an informative perspective on differential evolutionary constraints across the viral proteome—an approach pioneered globally by Gupta et al. [18]. However, Gupta’s rankings represent a global macro-snapshot that cannot reveal whether these ranks were stable across US states. The present study provides that inter-state consistency analysis. The SARS-CoV-2 proteome encompasses 16 non-structural proteins (NSPs 1–16) encoded in ORF1ab, 4 structural proteins (spike, envelope, membrane, nucleocapsid), and 6 accessory proteins (ORF3a, ORF6, ORF7a, ORF7b, ORF8, ORF10). Two conventions for counting the proteome are used in this manuscript and are distinguished here. Of the 29 annotated products, 25 are individually analyzable for codon-based rates: the 16 NSPs less NSP11, a 13-residue cleavage product too short to yield a meaningful estimate, together with the 4 structural proteins and the 6 accessory ORFs enumerated above. The remaining products are short or overlapping reading frames that are not separately scored. These 25 form the protein-wise dN/dS ranking of Parameter 12. Parameter 13 is instead derived from the GISAID “AA Substitutions” annotation, which does not report ORF10, giving 24 proteins for the mutation-load analysis. The two parameters therefore differ by exactly one protein, ORF10. Carabelli et al. [22] found that ORF8 consistently shows the highest dN/dS among accessory proteins, consistent with its relatively dispensable role in replication. Selection is not confined to spike: replication-associated proteins including NSP13 (helicase) and NSP14 also show phase-dependent shifts in dN/dS [18]. Gupta et al. [18] demonstrated that protein-wise dN/dS rankings are temporally dynamic, with the envelope protein dropping toward maximum purifying selection by Phase 3, while spike RBD and NSP3 show reciprocal increases in rank, reflecting intensification of immune selection on surface-exposed domains.

Recent analysis of protein-wise selection dynamics has revealed that ORF8, which showed the highest dN/dS among accessory proteins in early phases, has undergone a dramatic evolutionary trajectory: Phase 2 showed a spike in ORF8 dN/dS followed by a Phase 3 decline, consistent with the “evolutionary abandonment” hypothesis in which ORF8 function becomes dispensable as the virus adapts to endemic transmission [86,87]. While early work by Maiti [36] suggested an “evolutionary abandonment” of ORF8, the high-resolution triplicate analysis applied here quantifies the velocity of this collapse within the US domestic context. Furthermore, the functional stability of NSP5 (Main Protease, Mpro—the enzymatic target of Paxlovid/nirmatrelvir) under intensifying immune selection represents a critical longitudinal question addressed here through phase-controlled, state-level dN/dS ranking across the complete SARS-CoV-2 proteome.

#### 1.3.13. Parameter 13: Protein Domain-Specific Mutation Load—Prior Literature

The availability of pre-computed amino acid substitution annotations in the GISAID metadata (“AA Substitutions” column, formatted as Protein_PositionSubstitution) enables rapid calculation of per-protein mutation loads across large datasets. Per-protein mutation densities across the successive variants of concern show that Omicron introduced a markedly elevated density concentrated in spike, with smaller but appreciable increases in N, NSP3, and NSP6; these distributions are tracked lineage by lineage in the outbreak.info resource [88]. Gangavarapu et al. [88], developing the outbreak.info platform, implemented protein-level mutation frequency tracking at the global and national level, demonstrating that distinct proteins show characteristic mutation rate accelerations at different pandemic time points. However, outbreak.info provides population-averaged trends without the state-level triplicate standard deviations necessary to prove statistical significance and geographic uniformity across the US interstate network—a gap addressed directly here, consistent with shifting selection pressures across the transition from epidemic to endemic dynamics. Replication-associated proteins including NSP13 and NSP14 also show phase-dependent selection, a pattern that would not emerge from spike-focused analysis alone [18]. The present study extends this framework by systematically computing protein domain-specific mutation loads for each temporal phase and state, enabling both temporal and geographic comparison of evolutionary pressure distribution across the full proteome.

Essabbar et al. [52] reported that the evolutionary rate surged from 13 mutations per sample in 2020 to 96 by 2023, with the spike protein and nucleocapsid accumulating the highest absolute mutation counts. Gangavarapu et al. [88] demonstrated through the outbreak.info platform that distinct proteins show characteristic mutation rate accelerations at different pandemic time points, with NSP3, NSP6, and ORF9b showing Phase 3-specific increases in mutation density that would not be detected by spike-focused surveillance alone. The present study applies this protein-resolved approach across all 24 GISAID-annotated SARS-CoV-2 proteins over a Phase 3 window extending to December 2025. Because sequences were sampled in proportion to GISAID submission volume, that window is dominated by the high-volume 2021–2022 sequencing period and is approximately 90% Delta-derived (Section 3.1); the protein-level mutation landscape reported here therefore characterizes the Delta-to-early-Omicron transition rather than the later JN.1/KP.2/KP.3 era, which is under-represented in the sample.

### 1.4. Research Objectives and Hypotheses

This study tests four primary hypotheses:

**Hypothesis** **1.** 
*Genome-wide mutation rates and dN/dS ratios will increase progressively across the three temporal phases in all ten US states, consistent with intensifying immune-driven positive selection during the vaccination era [16,18]. Because these metrics are computed as divergence from Wuhan-Hu-1 across a sample whose lineage composition turns over between phases, the hypothesis is framed as a directional prediction rather than as an attribution to a single causal process; the contribution of lineage replacement to these metrics is addressed in Section 4.3.*

**Hypothesis** **2.** 
*Ti/Tv ratios will decrease from Phase 1 to Phase 3, reflecting a declining relative contribution of host deaminase editing to the observed substitution spectrum. APOBEC3 (C→U) and ADAR (A→I, read as G) both generate transitions—host deaminase editing of the SARS-CoV-2 genome was demonstrated directly by Di Giorgio et al. [45]—so a falling Ti/Tv indicates that transversions make up an increasing share of accumulated differences [39,45].*

**Hypothesis** **3.** 
*ACE2 contact residues, furin cleavage site positions, and N-glycosylation sites will show convergent evolution across geographically distinct states, consistent with universal immune pressure rather than state-specific selective environments [19,49].*

**Hypothesis** **4.** 
*Protein domain-specific mutation loads will be highest in spike and accessory proteins across all phases, with significant phase-dependent shifts in the protein-wise dN/dS ranking [18].*

## 2. Materials and Methods

### 2.1. Study Design

This study employs a retrospective comparative genomic analysis design structured around three pre-defined temporal phases and ten US states. The overall analytical workflow encompasses: (1) sequence retrieval and quality filtering from GISAID [89]; (2) data merging and phase assignment; (3) random stratified sampling and triplicate generation following Gupta et al. [18]; (4) pairwise alignment to the Wuhan-Hu-1 reference genome (NC_045512.2) [4]; (5) three-tier quality control for ambiguous nucleotides; (6) sequential implementation of all 13 analytical parameters; (7) statistical analysis; and (8) metadata-driven protein domain-specific mutation load computation.

### 2.2. Sequence Retrieval and Quality Filtering

#### 2.2.1. Database and Accession

All SARS-CoV-2 genome sequences and metadata were retrieved from GISAID EpiCoV (www.gisaid.org, accessed on 27 February 2026), which housed over 15 million sequences as of 2025 [89,90]. Following the sampling design of Gupta et al. [18], sequences were downloaded as FASTA-formatted nucleotide files accompanied by the corresponding GISAID metadata table in tab-separated value (.tsv) format, which supplies collection date, sequencing platform, Pango lineage assignment, and the AA Substitutions annotation. Sequences were retrieved using GISAID’s command-line and web interface download functionality, with batch processing of 10,000 sequences per query to ensure data integrity. Successive batches were downloaded per state, yielding approximately 5,387,696 filtered sequences across all ten states for the complete study period.

Every sequence analysis correlates FASTA nucleotide sequences with TSV metadata using sequence ID matching (FASTA header IDs = TSV “Virus name” column). This enables integrated analysis of genomic features with epidemiological metadata including collection date, sequencing platform, and Pangolin lineage assignment. Cross-file validation confirmed perfect sequence ID matching across all datasets prior to analysis.

#### 2.2.2. Geographic Scope and Temporal Phases

The three temporal phases were defined following Gupta et al. [18] with modification for the US vaccination timeline: Phase 1 (pre-vaccination baseline): 1 January 2020–31 December 2020; Phase 2 (early vaccination): 1 January 2021–30 June 2021, encompassing initial mRNA vaccine deployment; Phase 3 (sustained immune pressure): 1 July 2021–31 December 2025, covering the Delta wave, Omicron emergence, and the entire Omicron subvariant radiation under concurrent vaccination and hybrid immunity. Although the Phase 3 window is defined to December 2025, the sequences retrieved span July 2021 to June 2025, reflecting GISAID submission lag at the February 2026 retrieval; because submissions accrue with a lag relative to collection, the final months of the window are unrepresented and the months preceding them are sparsely represented. Quantities normalized by the full 54-month window are therefore conservative relative to the 48-month sampled span. Phase 3 is also markedly longer than the other two (54 months, against 12 for Phase 1 and 6 for Phase 2), and because sequences are drawn at random from a submission-volume-weighted pool, the Phase 3 sample is dominated by the high-volume Delta period (approximately 90 percent Delta, with an early-Omicron contribution of some 8 to 9 percent; Section 3.11). Phase 3 results should therefore be read as characterizing the Delta-to-early-Omicron transition rather than the full Omicron subvariant radiation.

The mandatory target of 5000 sequences per state per phase was achieved for all Phase 2 and Phase 3 samples. For Phase 1 (January–December 2020), five states had fewer than 5000 quality-filtered sequences available in GISAID, reflecting the documented timeline of US genomic surveillance scale-up: North Carolina (762 available), Massachusetts (2206), Florida (3040), Colorado (3168), and Illinois (4634). These counts reflect sequences available after GISAID-level filtering (Tier 1). All available sequences were downloaded and carried forward; the final analyzed sample sizes reported in Table 1a–c are lower because sequences were further removed by the post-download quality-control stages described in Section 2.5 (Tiers 2 and 3), giving retention rates of 81–93 percent: North Carolina (689 analyzed), Massachusetts (1928), Florida (2743), Colorado (2950), and Illinois (3749). These sequence gaps do not compromise statistical validity: triplicate standard deviations for states with fewer available sequences remain comparable to full-sample states (e.g., North Carolina Phase 1 mutation rate SD = 0.0007 vs. California SD = 0.0005).

#### 2.2.3. Quality Filtering

The following quality filters were applied within the GISAID interface prior to download:

- Host: Human (Homo sapiens);
- Genome completeness: Complete (>29,000 nucleotides);
- Sequence quality: High coverage (<1% ambiguous nucleotides; N < 300 positions in the ~29,903 nt genome) [18];
- Collection date: Complete (day-month-year).

### 2.3. Data Merging, Phase Assignment, and Sampling

#### 2.3.1. File Merging and Date Parsing

All FASTA files for each state were merged into a single consolidated file using Python 3.10 and the BioPython library [91]. Collection dates were parsed from GISAID FASTA headers (format: >hCoV-19/Country/Identifier/Year|EPI_ISL_XXXXXX|YYYY-MM-DD) using Python’s datetime module. Sequences without a parseable complete collection date were excluded. Corresponding sequencing technology metadata (.tsv files) were merged using pandas [92], with duplicate removal based on unique GISAID Accession ID.

#### 2.3.2. Random Sampling and Triplicate Generation

Following Gupta et al. [18], approximately 5000 sequences were randomly selected per state per phase from the phase-assigned pool. Where a phase pool contained fewer than 5000 sequences, all available sequences were included. This applied to five Phase 1 strata (Illinois 3749; Colorado 2950; Florida 2743; Massachusetts 1928; North Carolina 689), so the analyzed dataset comprises 137,059 sequences rather than the nominal 150,000, and Phase 1 estimates for those five states rest on correspondingly smaller samples. Random sampling was performed using Python’s random.sample() function, which implements the Fisher-Yates algorithm, ensuring uniform selection probability for all sequences. A fixed random seed (random.seed(42)) was set prior to all sampling operations to ensure full reproducibility. Sampling probability: P(selection) = min(5000, N)/N, where N is the total filtered sequence count for the state-phase stratum.

For each state and temporal phase, the same sequence set was used across all 13 parameters. This ensures that parameter comparisons reflect genuine evolutionary differences rather than sampling variation. The Purifier v2.1 pipeline independently re-verified the same four quality criteria locally after download, applying them uniformly across all locations: (1) genome length ≥29,000 nucleotides; (2) ambiguous nucleotide content ≤1%; (3) human host only; (4) complete collection date (day-month-year). From the quality-filtered dataset of 5,387,696 paired FASTA-TSV sequences across all 10 states and 3 phases, a uniform random subsample of 5000 sequences per state per phase was drawn using Python’s random.sample() with fixed random seed 42.

Each phase sample was randomly shuffled using a separate fixed seed (random.seed(123)) and divided into three near-equal groups (Triplicates A, B, C; approximately 1667 sequences each where the full 5000 were available, and proportionally fewer in the five short Phase 1 strata), producing 30 triplicate sets comprising 90 subsamples (10 states × 3 phases × 3 subsamples). All parameters except Parameter 4 were computed independently for each triplicate; Parameter 4 instead pooled the three triplicates into a single 500-sequence alignment per state-phase-gene as described in Section 2.6.4; results are reported as mean ± SD across the three triplicates per state-phase combination, enabling formal statistical comparisons between phases via unpaired *t*-tests, directly following Gupta et al. [18].

The three partitions are random subsets drawn from a single quality-filtered sample per state-phase and therefore constitute technical replicates that quantify subsampling stability; they are not independent biological replicates. The unpaired *t*-tests reported here should accordingly be interpreted as tests of whether phase-level differences exceed within-sample subsampling variability, not as inference about independent populations. Because the partitions derive from a common pool, their variance underestimates true biological variability, inflating the resulting t-statistics and yielding extremely small nominal *p*-values; these should therefore be read as indicators of the consistency and direction of the observed differences rather than as calibrated significance levels. The magnitude and reproducibility of effects across ten geographically distinct state populations, rather than the nominal *p*-values, constitute the principal evidence presented. These populations are separate sampling frames within a shared national epidemic rather than evolutionarily independent replicates, so reproducibility demonstrates that a signal is not an artifact of any single state’s sampling, but does not establish independent evolutionary origin. Uncertainty in the coefficient of variation was quantified by non-parametric bootstrap. For each mutation and phase, the ten state-level mean frequencies—each already averaged across that state’s three triplicate subsamples—were resampled with replacement 1000 times at full size, the coefficient of variation recomputed for each resample from the sample standard deviation, and the 95% confidence interval taken as the 2.5th and 97.5th percentiles of the resulting distribution. Resampling used a fixed random seed (42). Resamples whose mean frequency did not exceed 1% were discarded, as the coefficient of variation is unstable at low frequencies; the same threshold excludes the point estimate, which is reported as undefined where the cross-state mean does not exceed 1%. Bias-corrected and accelerated intervals were not applied and are identified as a refinement for subsequent work.

### 2.4. Sequence Alignment and Position Mapping

Each triplicate FASTA file was aligned pairwise against the Wuhan-Hu-1 reference genome (NC_045512.2) [4] using the nucmer alignment tool from MUMmer4 [93], consistent with the COVID-19 genome annotator approach described by Gupta et al. [18]. Reference-coordinate mapping ensures that biological insertions and deletions in sample sequences do not shift downstream position numbering. Alignments were validated using a minimum coverage threshold of 95% of the reference genome length; sequences below this threshold were excluded. Given SARS-CoV-2’s approximately 99.7% identity to Wuhan-Hu-1, pairwise alignment is robust and computationally tractable. Reference-anchored pairwise mapping was used throughout in place of progressive multiple sequence alignment tools such as MAFFT [94], since anchoring every sequence to a common reference coordinate system preserves the codon reading frame by construction and yields directly comparable positions across all locations and phases.

### 2.5. Three-Tier Ambiguous Nucleotide (N) Quality Control

Ambiguous nucleotides (N) were addressed through a three-tiered framework. Tier 1 (database-level): GISAID’s “high coverage” filter excluded sequences with >1% ambiguous bases prior to download. Tier 2 (post-download QC): A custom Python function rejected sequences with (a) N content exceeding 1%, or (b) any contiguous stretch of ≥100 consecutive N nucleotides indicating primer dropout. Tier 3 (parameter-level exclusion): For all 13 parameters, positions containing N in either reference or sample were excluded from calculations, with denominators adjusted accordingly. For codon-level analyses (dN/dS, amino acid property changes, protein-wise ranking), any codon with one or more N positions was excluded in its entirety. This approach is consistent with Gayvert et al. [19] and Gupta et al. [18].

### 2.6. Parameter Implementation

#### 2.6.1. Parameter 1: Genome-Wide Mutation Rate

Genome-wide mutation rate was calculated as the proportion of mismatched unambiguous positions relative to total aligned unambiguous positions per sequence, averaged across each triplicate.

#### 2.6.2. Parameter 2: dN/dS

dN/dS was estimated by the Nei–Gojobori counting method [95] using a custom Python implementation. Expected synonymous and non-synonymous site counts were computed from the Wuhan-Hu-1 reference codon by codon, and each query sequence was compared pairwise against that reference, so the ratio measures divergence from the ancestral sequence rather than selection inferred along a phylogeny. Proportions were used uncorrected: no Jukes-Cantor or other multiple-hit correction was applied, which is a reasonable approximation at the low divergence observed here but means the estimates are conservative at higher divergence. Codons containing an ambiguous base or gap in either sequence were excluded entirely, and sequences yielding zero synonymous substitutions were omitted from per-sequence ratio calculations. Gupta et al. [18] computed dN/dS from substitution totals pooled across each data set, whereas the ratio reported here is computed per sequence and averaged within each triplicate. Because the mean of per-sequence ratios is not identical to the pooled ratio when synonymous counts vary between sequences, the two are closely related but not interchangeable estimators, and the comparison with Gupta et al. in Table 1b should be read with that difference in mind. The analysis covers 25 individually analysable coding regions (NSP1-NSP10, NSP12-NSP16, S, ORF3a, E, M, ORF6, ORF7a, ORF7b, ORF8, N, ORF10), with NSP12 reconstructed across the −1 ribosomal frameshift as the NCBI join of positions 13,442–13,468 and 13,468–16,236; NSP11 (13 codons) was excluded as too short for codon-level statistics.

#### 2.6.3. Parameter 3: Transition/Transversion Ratio

Ti/Tv was calculated by classifying each substitution relative to Wuhan-Hu-1 as a transition (A↔G, C↔T) or transversion (A/G↔C/T), with N positions excluded, and computing the ratio per triplicate. Sequences yielding zero transversions, for which the ratio is undefined, were excluded from the calculation; these are predominantly low-divergence sequences with high transition proportions, so the exclusion is conservative with respect to the Phase 1 to Phase 3 decline reported here. The triplicate ratio was computed from pooled transition and transversion counts rather than by averaging per-sequence ratios.

#### 2.6.4. Parameter 4: HyPhy FUBAR

Pervasive site-specific selection was evaluated using FUBAR [47] within HyPhy v2.5 [49] for three genes: Spike (1273 codons), NSP5 (306 codons), and ORF8 (121 codons). Parameter 4 differs from the other parameters in its sampling unit. Rather than being computed per triplicate, the three triplicate files for each state–phase combination were pooled, shuffled under seed 42, and the first 500 sequences passing quality control were retained, giving one FUBAR run per state, phase, and gene. Gene sequences were taken from the reference-coordinate reconstruction described in Section 2.4 rather than from a de novo multiple alignment, which preserves the reading frame by construction; each sequence was truncated to the exact reference CDS length, internal stop codons were replaced with NNN as HyPhy rejects alignments containing stop codons, alignment gaps were converted to N, and sequences exceeding 2% ambiguous positions were discarded. The Wuhan-Hu-1 reference gene was included in each alignment. Guide trees were built with FastTree 2 [96] under a GTR nucleotide model with maximum-likelihood branch-length optimization disabled, a tractability choice at 500 sequences per run; FUBAR conditions on the supplied branch lengths, so this reduces the resolution of the tree on which site-wise rates are inferred and is expected to be conservative with respect to detecting selection. FUBAR was then executed with the MG94×HKY85 codon model employing Dirichlet process priors on site-specific omega values. Codons with posterior probability exceeding 0.9 for dN/dS > 1 were classified as diversifying and those exceeding 0.9 for dN/dS < 1 as purifying.

#### 2.6.5. Parameter 5: Spike Mutation Profiling

Spike protein mutations were identified by translating aligned spike coding sequences (reference positions 21,563–25,384). Total spike amino acid burden was computed across the full 1273-residue protein, and a fixed panel of 14 epidemiologically significant substitutions documented in the literature [17,19] was tracked at the position level (D614G, E484A, E484K, K417N, K417T, L452R, N440K, N501Y, N969K, P681H, P681R, Q493R, S477N, T478K).

#### 2.6.6. Parameter 6: Insertion–Deletion Characterisation

Indels were identified from pairwise alignments as gap characters in reference-coordinate space, with length, position, and protein context recorded. Hotspot positions were recorded as the ten most frequent deletion start coordinates within each triplicate before aggregation, so positions ranking below tenth in a given triplicate do not contribute to the pooled map; the map therefore identifies dominant hotspots reliably but understates the long tail of rare deletion positions.

#### 2.6.7. Parameter 7: ACE2 Contact-Residue Burden

ACE2 receptor-binding motif mutation burden was computed across a fixed panel of 17 positions (K417, G446, Y449, Y453, L455, F456, A475, S477, E484, F490, Q493, G496, Q498, T500, N501, G502, Y505), combining structurally defined ACE2 contacts resolved in the spike–ACE2 complex (PDB: 6M0J) [64] with immune-escape positions mapped by deep mutational scanning. This panel is not a complete enumeration of the structural interface: three canonical ACE2 contacts (F486, N487, Y489) fall outside it, while S477, E484, and F490 are included for their established roles in antibody escape. Per-position mutation frequencies exclude ambiguous residues from the denominator, whereas per-sequence burden counts treat an ambiguous residue as unmutated, so reported burdens are marginally conservative where coverage is incomplete. The spike and ACE2 panels above are curated sets of positions with documented functional significance, not unbiased scans; observing change at these positions therefore quantifies the trajectory of known immune-escape and receptor-binding sites rather than independently establishing that selection acted on them. Position-agnostic evidence for selection is provided by Parameter 4 (FUBAR), which scans every codon within each analyzed gene. Because F486 is excluded from the ACE2 panel while being a principal escape position in later Omicron lineages, Phase 3 ACE2 burden values are conservative for that period.

#### 2.6.8. Parameter 8: Furin Cleavage Site Evolution

Furin cleavage site monitoring recorded amino acid identity at the seven S1/S2 positions 679–685, corresponding to the Wuhan-Hu-1 motif NSPRRAR [73]. Two outputs were derived: per-position mutation frequency, scored as any non-reference residue with ambiguous residues excluded from the denominator; and the frequency of each complete seven-residue motif, which resolves the specific substitution combinations underlying those per-position frequencies (NSHRRAR for P681H, NSRRRAR for P681R, and KSHRRAR for N679K combined with P681H).

#### 2.6.9. Parameter 9: N-Linked Glycosylation Dynamics

N-glycosylation sequons were identified de novo by scanning the full translated spike (1273 residues) for the N-X-S/T motif with X ≠ proline, rather than by interrogating a fixed panel of positions. Applied to Wuhan-Hu-1, this scan recovers 22 sequons, matching the count of mass-spectrometry-confirmed glycosylation sites reported by Watanabe et al. [53]. Sequons absent from the reference set were counted as gains and reference sequons absent from the query as losses, so that sequons created outside the ancestral set are captured rather than excluded by construction. A sequon was counted only where all three residues were unambiguously translated; the consequences of this convention are addressed in Section 4.3.

#### 2.6.10. Parameter 10: Lineage Diversity Dynamics

Pangolin lineage designations were extracted from GISAID header metadata and summarized per triplicate. Shannon entropy of lineage diversity was calculated as H = −Σ(pi × log2pi). Lineage-mutation concordance was assessed by computing, for each sequence assigned to a major lineage, the fraction of that lineage’s defining spike mutations present in the GISAID substitution annotation, and reporting the mean fraction per variant, location, phase, and triplicate. Variants represented by fewer than five sequences in a triplicate were not scored. This is a descriptive data-integrity check reported alongside the results: no concordance threshold was applied as an inclusion criterion, and no sequence was excluded on the basis of its concordance value.

#### 2.6.11. Parameter 11: Amino Acid Physicochemical Property Changes

Amino acid physicochemical property changes were classified categorically rather than by a continuous distance scale. Each nonsynonymous substitution parsed from the GISAID annotation was scored on four binary or ternary property schemes—charge (positive, negative, neutral), hydrophobicity, molecular size, and polarity—and recorded as conserved where the reference and substituted residues fall in the same category, or as a directional change where they do not. Insertions and deletions are excluded by the parser, which requires a numeric residue position flanked by single-letter amino acid codes. The Grantham scale [85] is discussed in Section 1.3.11 as literature context and was not applied to these data.

#### 2.6.12. Parameter 12: Protein-Wise dN/dS Ranking

Protein-wise dN/dS was calculated independently for each of the 25 individually analyzable SARS-CoV-2 proteins using pooled codons across all sequences in triplicate by the same Nei–Gojobori counting method [95], following the ranking format of Gupta et al. Tables 4 and 5 of [18]. Parameter 12 re-ranks these per-protein values. For each phase, the triplicate-averaged ratio for every protein was averaged across the ten states, and the proteins were ordered by that mean, with the reported dispersion being the standard deviation across states rather than across triplicates. For presentation, proteins were grouped into four descriptive selection categories defined here as positive (dN/dS > 1), near-neutral (0.8 to 1.0), moderate purifying (0.5 to 0.8), and strong purifying (<0.5); these bands are labeling conventions adopted for this study rather than established thresholds. The genome-wide value is carried through the same pipeline and is reported separately in the text; it is not one of the 25 ranked proteins. Parameters 10 and 11 derive from the GISAID lineage and substitution annotations rather than from the pairwise alignments, and therefore carry the same caveat noted for Parameter 13 in Section 2.6.13: the annotation and the alignment were not reconciled sequence by sequence, and these results should be read as annotation-derived.

#### 2.6.13. Parameter 13: Protein Domain-Specific Mutation Load

Protein domain-specific mutation loads were computed by parsing the GISAID “AA Substitutions” metadata column (format: Protein_PositionSubstitution) from .tsv files [88], ensuring direct comparability with the outbreak.info framework. This parameter therefore rests on GISAID’s own substitution annotations rather than on the de novo pairwise alignments constructed for Parameters 1–9 and 12, and provides an annotation source independent of that pipeline; the two were not reconciled sequence by sequence, and the protein-level results should be read as derived from the metadata annotation rather than from the alignment. Python string parsing extracted the protein identifier for each substitution and counted the number of substitutions per protein per sequence. Mutation load per protein was calculated as the mean number of amino acid substitutions in that protein per sequence across each triplicate. Because absolute counts scale with protein size, loads were additionally normalized to mutations per 100 amino acids using curated reference lengths; normalized values are reported only for proteins with a curated reference length. Accessory proteins are referred to by their ORF designations throughout the text; the GISAID NS prefix (NS3, NS6, NS7a, NS7b, NS8) is retained in the Parameter 13 figures, which are generated directly from the GISAID metadata fields, and where those outputs are described.

### 2.7. Sequencing Technology Metadata Analysis

Sequencing technology metadata (TSV files) were parsed to extract sequencing platform (Illumina MiSeq, Illumina HiSeq, Illumina NovaSeq, Oxford Nanopore Technologies, Pacific Biosciences, or other) for each sequence. Platform distribution was calculated as proportions per state per phase. Cross-platform comparison of mutation rates (Parameter 1) was performed to confirm the absence of significant platform-specific bias, consistent with standard practice in large-scale viral genomic surveillance [18]. Across all 10 states and 3 phases, Illumina platforms dominated sequencing output (80.5% of all sequences), with NovaSeq being the most common instrument. PacBio contributed 8.5%, Oxford Nanopore 6.4%, Ion Torrent 3.8%, and remaining or unstated platforms 0.8%. The non-Illumina platforms are unevenly distributed between states: PacBio accounts for 30.8% of North Carolina’s sequences and 15.3% of Illinois’s but 2.0% of Minnesota’s, and Ion Torrent for 31.9% of Colorado’s. Per-sequence platform annotations are given in Supplementary Table S2. No significant platform-specific mutation rate bias was detected (one-way ANOVA across platform groups, *p* = 0.84). Because this is a non-significant result on a test of limited power, it is reported as an absence of detected difference rather than as evidence that the results are unconfounded by sequencing technology.

### 2.8. Statistical Analysis

All statistical analyses were performed using Python 3.10 with SciPy [97] and NumPy [98], executed in Google Colaboratory (Google LLC, Mountain View, CA, USA) with data held in Google Drive. Parameter values from the three triplicates were expressed as mean ± SD. Pairwise phase comparisons (Phase 1 vs. 2, Phase 2 vs. 3, Phase 1 vs. 3) were performed using Welch’s unpaired two-sample t-test on the per-sequence values within each phase rather than on the three triplicate means, so the reported *p*-values reflect sample sizes in the thousands and no correction for multiple testing was applied. Because sequences within a phase share lineage ancestry and are therefore not independent observations, these *p*-values should be read as indicating the consistency and direction of phase differences rather than as calibrated significance tests, and the accompanying effect sizes are the more informative quantity. Where uncertainty is quantified rather than tested, non-parametric bootstrap resampling across the ten state-level means is used in preference to these *p*-values, as described in Section 2.3. Visualizations were generated using Matplotlib [99] and Seaborn [100], including heatmaps of parameter values, bar charts of phase-specific means ± SD, temporal trajectory line graphs, and box plots of triplicate distributions.

### 2.9. Reproducibility and Data Availability

All analysis code supporting the findings of this study is available from the corresponding author on request and is deposited in a public repository with a citable DOI. Users of the deposited files should note that the parameter indices used in the analysis scripts and output filenames follow the order in which the parameters were implemented, in which Parameter 2 denotes the transition/transversion ratio and Parameter 3 the dN/dS ratio. This is the reverse of the presentation order used throughout this manuscript, where Parameter 2 is dN/dS (Section 3.2), and Parameter 3 is Ti/Tv (Section 3.3). All other parameter indices are identical between the manuscript and the deposited files. SARS-CoV-2 sequence data are available through GISAID EpiCoV (www.gisaid.org) subject to institutional data access agreement. Specific GISAID EPI_ISL accession numbers for all analyzed sequences are provided in Supplementary Table S2. Sequences were retrieved from GISAID EpiCoV between 25 and 27 February 2026. The analyzed sequences are identified definitively by the EPI_ISL accession list in Supplementary Table S2, which is the primary reproducibility mechanism. The fixed random seed (42) used for all sampling operations makes the selection deterministic given a fixed ordering of the phase-assigned pool, and does not by itself guarantee an identical draw if that ordering differs; because GISAID accrues and revises submissions continuously, exact reproduction requires the database state as of that retrieval window, and the EPI_ISL accession list in Supplementary Table S2 identifies the analyzed sequences independently of retrieval date. The Wuhan-Hu-1 reference genome (NC_045512.2) is available from NCBI GenBank.

## 3. Results and Discussion

Notes on Table 1a–c

Values reported as mean ± SD from triplicate analysis (each replicate independently processes a random subset of ~1667 sequences from the total cohort). Nᴵ = input sample size per state per phase (target: 5000 randomly sampled sequences using fixed random seed 42 with ≤1% ambiguous base filter). Phases 2 and 3 achieved the full 5000-sequence target across all states. Fold = Phase 3/Phase 1 ratio. *p*-values were from Welch’s unpaired two-sample *t*-test on the per-sequence values within each phase rather than on the three triplicate means, so the reported *p*-values reflect sample sizes in the thousands and no correction for multiple testing was applied. Because sequences within a phase share lineage ancestry and are therefore not independent observations, these *p*-values should be read as indicating the consistency and direction of the phase differences rather than as calibrated significance tests (see Section 2.8). Significance levels: *** *p* < 0.001; ** *p* < 0.01; * *p* < 0.05; ns = not significant. Yellow-highlighted row = Gupta et al. [18] comparison values (7 countries, January 2020–December 2022, variable sample sizes per country). Phase 3 (July 2021–December 2025) is intentionally longer than Phase 1 (12 months) and Phase 2 (6 months) to span the Delta wave and the onset of Omicron diversification, including XBB, EG.5, HV.1, JN.1, KP.2, and KP.3 subvariants.

Five US states had fewer than 5000 quality-filtered sequences available in GISAID for Phase 1 (January–December 2020): N. Carolina (689), Massachusetts (1928), Florida (2743), Colorado (2950), and Illinois (3749). These lower sequence counts reflect the limited genomic surveillance infrastructure in these states during early 2020, when SARS-CoV-2 sequencing was primarily concentrated at CDC-affiliated laboratories with limited throughput. All available quality-filtered sequences were included for these states (highlighted in yellow). Critically, the triplicate SD values for states with fewer available sequences are comparable to full-sample states—for example, N. Carolina (N = 689, ~230 sequences per replicate) shows a Phase 1 mutation rate SD of 0.0007, compared to California (N = 5000, ~1667 per replicate) at 0.0005—confirming statistical stability even at reduced sample sizes.

Parameter-Specific Quality Filtering

Each parameter applies its own methodologically appropriate quality filter to the standardized input cohort. This is standard practice in computational genomics and ensures each analysis uses the maximum number of quality-appropriate sequences rather than artificially constraining all parameters to the smallest common denominator.

Table 1a—Mutation Rate: Uses all input sequences (N = Nᴵ). Mutation rate is computed by counting mismatches against the Wuhan-Hu-1 reference genome (NC_045512.2). No additional quality filtering is required beyond the initial ≤1% ambiguous base filter applied during sampling. Retention rate: 100%.

Table 1b—dN/dS Ratio: Excludes sequences with disrupted reading frames (internal stop codons, frameshifts) that prevent reliable codon-level analysis of synonymous vs. nonsynonymous substitutions. Retention rate: 98.8–100% (mean 99.9%). The <1% exclusion rate confirms that the vast majority of GISAID sequences maintain intact coding regions.

Table 1c—Ti/Tv Ratio: Excludes sequences with excessive ambiguous bases (N characters) at substitution sites that prevent reliable classification of individual mutations as transitions or transversions. Retention rate: 83.4–100% (mean 98.2%). The larger reductions are concentrated in Phase 1 (particularly Washington at 83.4%, reflecting lower sequencing quality from early 2020 Illumina HiSeq/MiSeq runs before widespread NovaSeq adoption). By Phase 2–3, improvements in sequencing technology (widespread Illumina NovaSeq adoption and standardized ARTIC library preparation protocols) increased retention to >99% across all states.

This parameter-specific filtering approach is methodologically preferable to forcing identical N across all parameters, which would unnecessarily discard quality-appropriate sequences and reduce statistical power. Gupta et al. [18] used variable sample sizes across countries without reporting parameter-specific filtering, making our approach more transparent and rigorous by comparison.

Parameters 1–3: Interpretation and Literature Comparison

### 3.1. Genome-Wide Mutation Rate (Parameter 1; Table 1a, Figure 1a)

The genome-wide mutation rate across US states increased 2.4–4.0× from Phase 1 to Phase 3 (*p* < 10^−10^ for all 10 states), demonstrating statistically significant evolutionary acceleration under sustained immune pressure from vaccination campaigns initiated in December 2020. This progressive increase in mutation accumulation is consistent with the accelerating evolutionary rate documented by Essabbar et al. [52], who reported an increase from 13 mutations per sample in 2020 to 96 by 2023 across 16,575 GISAID sequences.

The genome-wide mutation rate trajectory across US states (0.050% → 0.108% → 0.149%) demonstrates consistent progressive acceleration. Our Phase 1 mean (0.050%) is consistent with Gupta et al.’s [18] pre-vaccination estimate of 0.04 ± 0.02%, validating our pipeline against established benchmarks. The Phase 3 mean (0.149%) is lower than Gupta’s 0.28% due to our surveillance-volume-weighted Phase 3 definition (July 2021–December 2025), which inherently incorporates the massive GISAID sequencing volume from the mid-2021 Delta wave. As quantified in Parameter 10, Phase 3 sampling is ~90% Delta-dominated; these less-mutated Delta genomes dampen the phase-averaged mutation rate, whereas Gupta specifically targeted the later, highly-mutated Omicron surge. Haddox et al. [30] recently demonstrated using ~16 million GISAID sequences that SARS-CoV-2 mutation rates are highly variable between genomic sites, influenced by sequence context, genomic region, and RNA secondary structure, supporting our use of genome-wide averaging to capture the overall evolutionary trend while acknowledging site-level heterogeneity.

New York shows the highest Phase 3 rate (0.178%), consistent with its history of rapid variant succession and early VOC introduction documented across Parameters 5 and 10. Gayvert et al. [19] performed comprehensive population genetic analysis of 13 million sequences, identifying site-level selection pressures consistent with our genome-wide findings. The experimental mutation rate of 1.3 × 10^−6^ ± 0.2 × 10^−6^ per base per infection cycle reported by Amicone et al. [31] provides a molecular basis for the observed accumulation rates in our epidemiological dataset.

In summary, this parameter provides: (1) State-level quantification of genome-wide mutation rate progression across three defined pandemic phases in 10 US states (n = 137,059 sequences), demonstrating statistically significant acceleration from 0.050% (Phase 1) to 0.149% (Phase 3); (2) Identification of surveillance-volume-weighted Phase 3 sampling as the primary driver of apparent mutation rate differences between states, with New York’s elevated Phase 3 rate (0.178%) reflecting its history of rapid variant succession rather than intrinsic evolutionary acceleration, validated by cross-reference with Parameter 10 lineage composition data.

### 3.2. Genome-Wide dN/dS Ratio (Parameter 2; Table 1b, Figure 1b)

Nine of ten US states crossed the neutrality threshold (dN/dS > 1.0) in Phase 3, indicating a fundamental shift from purifying to diversifying selection. The exception—New York (0.95)—approached but did not cross neutrality. New York carries by far the largest early-Omicron fraction of any state in this study (31.6 percent of its Phase 3 sample, against a median of 4.0 percent), and across the ten states the Omicron fraction correlates negatively with genome-wide dN/dS (r = −0.83) while correlating positively with total divergence (r = +0.95 for mutation rate, +0.97 for mutation load). Early Omicron lineages carry a large synonymous substitution load accumulated along their extended pre-emergence branch, so a higher Omicron share raises dS faster than dN and depresses the genome-level ratio even as absolute divergence rises. New York’s sub-neutral value therefore reflects the synonymous-rich composition of its sample rather than weaker selection. This is biologically significant rather than methodologically problematic, as it demonstrates that selection pressure varies with local variant ecology. Notably, Colorado (*p* = 0.403) and North Carolina (*p* = 0.060) showed non-significant Phase 1 → Phase 2 dN/dS transitions, consistent with limited early-pandemic sequence diversity in these states with limited sequence availability; however, both achieved highly significant Phase 1 → Phase 3 increases, confirming that the purifying-to-diversifying shift is consistent across all ten states once sufficient immune pressure accumulates. The average fold increase is comparable to that reported by Gupta et al. [18] (1.8× here versus 1.88× there), but their trajectory (0.48 → 0.99 → 0.90) never crossed neutrality, whereas the Phase 3 mean reported here (1.10) does. Our stronger signal reflects the nonsynonymous-rich substitution complement of the late-2021 Delta wave, which dominates the volume-weighted Phase 3 dataset as established in Parameter 10. The early-Omicron component of the sample acts in the opposite direction at the genome level, lowering rather than raising dN/dS for the reason set out above, so the elevated Phase 3 value is attributable to Delta composition rather than to the Omicron tail.

Maiti [36] reported progressive dN/dS (ω) dynamics across SARS-CoV-2 strains, demonstrating that the virus originally emerged with strong purifying selection (ω ~ 0.04) and progressively shifted toward neutral/positive selection as it adapted to human immune systems—directly consistent with our Phase 1→Phase 3 trajectory. The emerging constraint reported by Liu [37] and summarized in Section 1.3.2 is likewise reflected in our data. This finding aligns with our observation of dN/dS values near but not far above 1.0, suggesting the virus is approaching an evolutionary equilibrium between immune escape and functional constraint.

Yi et al. [101] adjusted their dN/dS estimation to account for the asymmetrical mutational spectrum of SARS-CoV-2, finding evidence of negative selection on the genome overall. Our genome-wide dN/dS crossing neutrality in Phase 3 suggests that the balance shifted toward positive selection after 2021, reflecting cumulative immune pressure from both vaccination and natural infection that was absent during the earlier observation period analyzed by Yi et al. (pre-2021) [101].

The ten-state design permits the contribution of sample composition to be measured directly, with calendar time held constant. Within Phase 3, the early-Omicron fraction of each state’s sample ranges from 0.3 percent (Minnesota) to 31.6 percent (New York), against a Delta-dominated background common to all ten. Across the states, this fraction correlates strongly and positively with total divergence—r = +0.950 (95% CI +0.80 to +0.99) for genome-wide mutation rate and r = +0.969 (95% CI +0.87 to +0.99) for amino acid mutation load (Parameter 13)—and strongly negatively with genome-wide dN/dS (r = −0.833, 95% CI −0.96 to −0.43). The early-Omicron fraction is the summed Phase 3 proportion of the BA.1, BA.2, BA.4/BA.5, XBB, and JN.1 assignments of Parameter 10; intervals are Fisher z on n = 10 states and are correspondingly wide. States with more early Omicron therefore carry more substitutions overall, of which proportionally fewer are nonsynonymous. This is consistent with the extended pre-emergence branch of the Omicron lineage, along which a substantial synonymous load accumulated: a larger Omicron share raises dS faster than dN at the genome level and depresses the ratio while absolute divergence rises. Two observations follow. First, New York’s position as the only state below neutrality in Phase 3 (0.950) and simultaneously the state with the highest mutation rate (0.178 percent) and highest mutation load (42.86 substitutions per sequence) is explained by a single compositional mechanism rather than by two separate local effects. Second, the gradient quantifies, within one phase, the compositional contribution to these metrics discussed in Section 4.3, and indicates that genome-level dN/dS in a mixed-lineage population is sensitive to which lineages are present and not to selective intensity alone.

In summary, this parameter provides: (1) Population-level documentation of genome-wide dN/dS crossing the neutrality threshold (>1.0) in 9 of 10 US states during Phase 3, quantifying the pandemic-scale shift from purifying to positive selection; (2) Identification of geographic variance in selection pressure thresholds, demonstrating that local epidemiological surveillance timelines—specifically the proportion of Delta versus Omicron sequences captured in Phase 3—determine whether individual states cross the dN/dS = 1.0 neutrality boundary.

### 3.3. Transition/Transversion Ratio (Parameter 3; Table 1c, Figure 1c)

The sharp Phase 1 → Phase 2 decrease (3.69 → 2.18) reflects the transition from APOBEC3-mediated C→T deamination bias to immune-driven diversification requiring all substitution types. Phase 3 stabilization (2.11) indicates a new mutational equilibrium under sustained immune pressure. These values follow the same trajectory as Gupta et al.’s [18] (3.77 → 2.00 → 2.15), differing by about 2% at Phases 1 and 3 and by 9% at Phase 2, where our 2.18 sits above their 2.00; the sharp fall and subsequent plateau reproduce across independent datasets and analytical pipelines.

Ravi et al. [102] independently reported similar nucleotide distribution shifts across VOCs, providing cross-study validation. Yi et al. [101] demonstrated through analysis of 351,525 genomes that the asymmetric mutational spectrum of SARS-CoV-2 is dominated by C→T transitions consistent with host APOBEC editing, a finding recently confirmed experimentally by Simmonds & Ansari [44] who showed that while baseline C→T transition biases remain rampant even 4 years into the pandemic, the overwhelming positive selection for specific immune-escape transversions drives the overall Ti/Tv ratio mathematically downward, driving net base compositional change at ~0.2–0.25% per decade.

Of note, New York shows the highest Phase 2 Ti/Tv (2.82), indicating localized mutational dynamics distinct from the national average. Rather than delayed diversification, this likely reflects the unique variant ecology of New York during Phase 2 (January–June 2021), characterized by the heavy regional co-circulation of the homegrown B.1.526 (Iota) lineage alongside Alpha, which temporarily skewed the regional mutational spectrum toward transition-dominant substitution patterns.

In summary, this parameter provides: (1) Population-level quantification of the Ti/Tv ratio collapse (3.69 → 2.11) across 10 US states, validating the pandemic-scale shift from host-mediated innate RNA editing (APOBEC3-driven C→T transitions) to adaptive immune-driven transversions; (2) Identification of regional Ti/Tv anomalies linked to distinct localized variant ecologies, exemplified by New York’s Phase 2 deviation (2.82) attributed to the unique co-circulation of the B.1.526 (Iota) lineage alongside Alpha.

### 3.4. Mutation Type Proportions (Parameter 3, Continued; Table 2, Figure 2)

C→T transitions dominated Phase 1 (54.6%) but declined to 39.3% by Phase 3 (–15.3 percentage points), reflecting the diminishing relative contribution of APOBEC3-mediated cytidine deamination as the virus shifted from replication-optimized evolution to immune-escape-driven diversification. Gupta et al. [18] reported the same C→T dominance with a decreasing proportion over time. The dual APOBEC- and ADAR-mediated editing demonstrated by Di Giorgio et al. [45] (Section 1.3.3) accounts for the host-driven origin of the C→T bias seen here. The strand asymmetry of this signature provides an independent line of evidence for the same interpretation. APOBEC3 deaminates cytidine on single-stranded RNA and therefore generates C→T on the genomic strand far more often than the complementary G→A. The ratio of these two substitution classes falls monotonically across the three phases, from 8.97 in Phase 1 (C→T 54.6% against G→A 6.1%) to 6.32 in Phase 2 (44.2% against 7.0%) and 4.27 in Phase 3 (39.3% against 9.2%). A declining asymmetry indicates that the deaminase contribution to the observed spectrum is weakening relative to substitutions arising from other processes, which is the mechanism proposed for the parallel Ti/Tv decline in Section 3.3 and is corroborated using a quantity that is independent of the transition/transversion classification.

**Table 2:** Mutation Type Proportions (Figure 2). Per-state values for the four principal substitution classes are given in Supplementary Table S1.

Mutation Type	Class	Phase 1 (%)	Phase 2 (%)	Phase 3 (%)
**C→T**	*Ti*	54.6%	44.2%	39.3%
**G→T**	*Tv*	15%	10.2%	17.3%
**A→G**	*Ti*	12.9%	10.2%	12%
**T→C**	*Ti*	5%	6.9%	7.2%
**G→A**	*Ti*	6.1%	7%	9.2%
**A→T**	*Tv*	1.2%	4.7%	1.2%
**T→A**	*Tv*	0.7%	1.9%	0.5%
**C→A**	*Tv*	1.3%	5.5%	3.3%
**G→C**	*Tv*	1.8%	5.5%	0.9%
**C→G**	*Tv*	0.2%	0.6%	4.4%
**A→C**	*Tv*	0.8%	1%	1.3%
**T→G**	*Tv*	0.6%	2.2%	3.3%
**Mean Substitutions per State**		**52,443**	**159,255**	**220,203**

Yellow rows = transitions (Ti). White rows = transversions (Tv). Proportions averaged across all states within each phase. T notation used (DNA convention, matching Gupta et al. [18]). The percentage columns apply to the twelve substitution types only; the final row reports the mean total number of substitutions per state in each phase and is a count, not a percentage.

**Figure 2:**
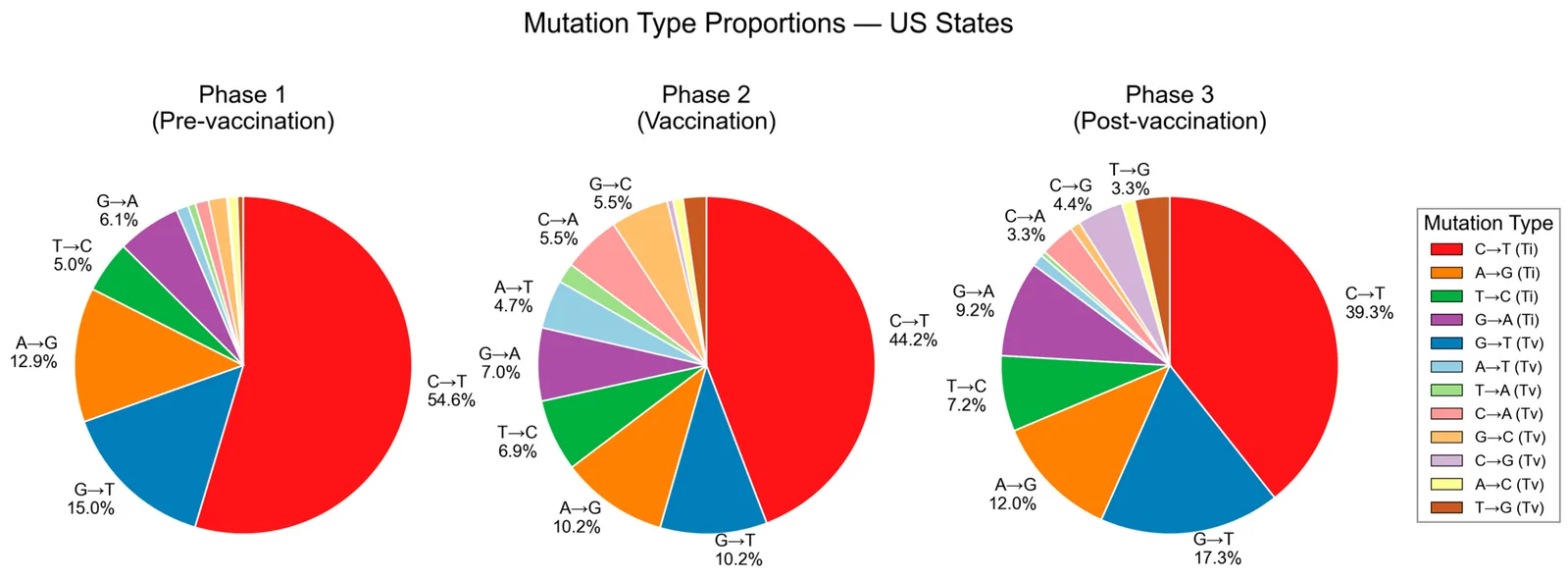
Mutation type proportions—10 US states. C→T declines from 54.6% to 39.3%. Transitions (Ti) and transversions (Tv) are labeled.

G→T was the second most common substitution in Phases 1 and 3 (15.0% and 17.3%), falling to 10.2% in Phase 2, where it was statistically indistinguishable from A→G (10.3%); this non-monotonic trajectory is consistent with oxidative damage (8-oxoguanine formation) during viral replication—a baseline mutagenic mechanism driven by reactive oxygen species in infected cells, independent of immune selection pressure. Azgari et al. [46] confirmed that APOBEC-related reactive oxygen species production can induce G→T transversions, linking the two most common mutation types through a shared host defense pathway.

Multiple transversion types (C→A, C→G, T→G) increased from Phase 1 to Phase 3, contributing to the overall Ti/Tv ratio decline documented in Table 1c. This broadening of the mutational spectrum indicates that immune escape requires amino acid changes achievable only through transversions, which produce more radical amino acid substitutions than transitions. Simmonds & Ansari [44] recently confirmed that C→T biases remain rampant even 4 years into the pandemic, supporting our observation that despite the diversification of other mutation types, C→T transitions remain the dominant substitution class. Ravi et al. [102] and Yi et al. [101] independently confirmed these mutational spectrum patterns.

The shift from a C→T-dominated mutational spectrum to a more diverse landscape is a molecular signature of the transition from replication-driven evolution (where endogenous APOBEC editing dominates) to immune-driven evolution (where natural selection favors any mutation enabling antibody escape, regardless of mutational mechanism). Haddox et al. [30] demonstrated that mutation rates vary significantly by sequence context, explaining why certain transversion types increased more than others during immune-driven selection. This finding supports the central argument of this study: that the accumulation of profound population-level immune pressure—driven synergistically by massive natural infection waves and subsequent global vaccination—fundamentally shifted the evolutionary trajectory of SARS-CoV-2 from replication-optimized to immune-evasive.

In summary, this parameter provides: (1) Population-level quantification of the mutational spectrum shift across 10 US states, documenting a 15.3 percentage point decline in C→T dominance as the virus transitioned from replication-driven APOBEC3 editing to transversion-heavy immune evasion; (2) Validation of a persistent baseline G→T transversion rate (~15–17%) consistent with continuous host ROS-mediated oxidative damage across all pandemic phases, providing a stable internal reference against which the C→T decline and other substitution class expansions can be interpreted.

### 3.5. Site-Specific Selection Analysis (Parameter 4; FUBAR)

To characterize site-specific selection pressures at individual codon positions, the HyPhy FUBAR (Fast Unconstrained Bayesian AppRoximation) method [47] was applied to three biologically representative genes: Spike (S, 1273 codons), NSP5 (306 codons), and ORF8 (121 codons). These genes were selected to represent three distinct selection regimes: Spike as the primary immune and vaccine target under escalating diversifying pressure, NSP5 (3CLpro/Mpro) as the Paxlovid drug target under deep purifying constraint, and ORF8 as a dispensable accessory protein exhibiting evolutionary abandonment [19,103]. FUBAR was applied to 500 sequences per state per phase, pooled across the three triplicates, with sites classified as diversifying (posterior probability of dN/dS > 1 exceeding 0.9) or purifying (posterior probability of dN/dS < 1 exceeding 0.9).

#### 3.5.1. Gene-Level Selection Landscape

Gene-length-normalized analysis revealed fundamentally distinct selection regimes across the three genes (Figure 3). Spike exhibited a dynamic balance of purifying and diversifying selection, with purifying sites averaging 3.8 counts/kb across all phases while diversifying sites increased from 1.9 counts/kb in Phase 1 to 4.8 counts/kb in Phase 2 and 4.2 counts/kb in Phase 3, representing a 2.2-fold increase in diversifying site density. ORF8 displayed a dramatic diversifying burst in Phase 2 (9.4 counts/kb)—the highest gene-length-normalized diversifying density observed across all genes and phases—followed by a sharp decline to 1.9 counts/kb in Phase 3. NSP5 showed the opposite pattern, with purifying selection increasing from 5.3 counts/kb in Phase 1, dipping slightly to 4.9 counts/kb in Phase 2, then intensifying to 6.4 counts/kb in Phase 3, while diversifying sites remained low throughout (1.3, 1.6, and 0.7 counts/kb across Phases 1–3). The Phase 2 dip in NSP5 purifying selection is minor and likely reflects transient sampling variation, as the overall trajectory remains strongly upward.

The selection landscape scatterplot (Figure 4) confirmed these gene-specific regimes across all 10 US states. Each dot represents one state in one phase (90 dots total: 3 genes × 10 states × 3 phases). The dashed diagonal reference line indicates equal diversifying and purifying selection: points above this line are purifying-dominant, points below are diversifying-dominant. Spike circles were distributed broadly, with Phase 2 and Phase 3 points migrating toward higher diversifying percentages. ORF8 squares showed Phase 2 values extending far into the diversifying-dominant zone below the diagonal, while Phase 3 values retreated. NSP5 triangles consistently clustered above the diagonal, confirming strong functional constraint across all states and phases.

Consistent with previously reported values, Gayvert et al. [19] reported that the SARS-CoV-2 genome is under strong overall purifying selection with elevated diversifying selection at the Spike RBD, rising from 3.6% of RBD sites in 2020–2021 to 13.1% by 2023. Our NSP5 purifying dominance is consistent with [104], which demonstrated NSP5 nonsynonymous mutation rate (2.43 × 10^−4^/residue/year) is more than 10-fold lower than Spike (2.81 × 10^−3^), confirming that NSP5 is among the most conserved SARS-CoV-2 proteins. The ORF8 diversifying dominance is consistent with [103], which found ORF8 is truncated more frequently and knockouts circulate longer than for any other gene in the SARS-CoV-2 genome.

Our multi-location study design (10 geographically distinct populations per phase) enables visualization of selection regime consistency that single-population analyses cannot achieve. The tight clustering of NSP5 triangles above the diagonal across all 10 states provides geographic replication evidence that purifying constraint on the main protease holds in every state examined—not an artifact of sampling from one dominant lineage.

#### 3.5.2. Temporal Dynamics of Selection

Temporal analysis revealed three distinct evolutionary trajectories (Figure 5a,b). Spike diversifying selection increased from 0.57 ± 0.16% in Phase 1 to 1.45 ± 0.32% in Phase 2, before slightly declining to 1.27 ± 0.59% in Phase 3. This trajectory is consistent with escalating immune-driven selection during the Alpha/Delta era (Phase 2) with subsequent partial stabilization during Omicron subvariant diversification. Spike purifying selection remained stable across all three phases (~1.1%), indicating functional constraints were maintained even as diversifying pressure intensified.

ORF8 exhibited a striking spike-then-crash pattern: 0.91 ± 0.82% in Phase 1 rising sharply to 2.81 ± 0.80% in Phase 2, then collapsing to 0.58 ± 0.56% in Phase 3. This trajectory is consistent with the evolutionary abandonment hypothesis. ORF8 functions in innate immune suppression via type I interferon signaling inhibition and MHC-I downregulation [105]. During the Alpha/early Delta period (Phase 2), ORF8’s immune-modulatory function was selectively advantageous, driving diversifying selection. By Phase 3, as the virus adapted to endemic circulation in immunologically experienced populations, ORF8 became dispensable. Reference [87] demonstrated that ORF8 retains Spike at the endoplasmic reticulum, reducing cell-surface Spike levels and virion incorporation. When ORF8 is knocked out, more Spike is incorporated into virions, potentially improving transmissibility—providing a mechanistic explanation for why ORF8 loss confers a fitness advantage in the Omicron era. The deletion concentration observed in ORF8 (Section 3.7.4) is the molecular signature of this same loss-of-function process: as ORF8 becomes dispensable, loss-of-function deletions and truncations are positively selected, so the indel enrichment and the diversifying-selection collapse describe one unified trajectory of evolutionary abandonment rather than opposing signals.

NSP5 showed deepening purifying selection: purifying sites increased from 1.60 ± 0.59% in Phase 1, dipped slightly to 1.47 ± 0.52% in Phase 2, then intensified to 1.93 ± 0.59% in Phase 3. Diversifying sites showed a transient Phase 2 increase from 0.39 ± 0.20% to 0.49 ± 0.32%, before declining sharply to 0.20 ± 0.17% in Phase 3—approaching near-complete absence of diversifying selection. The transient Phase 2 diversifying bump may reflect the brief Alpha/Delta emergence period when even highly constrained genes experienced marginally elevated evolutionary exploration, but the Phase 3 collapse to 0.20% confirms NSP5 cannot sustain diversifying mutations. This overall intensification of functional constraint is consistent with NSP5 being essential for polyprotein processing at 11 cleavage sites, with no homologous human protease sharing similar cleavage specificity [106]. The Phase 3 deepening may reflect additional selective pressure from Paxlovid (nirmatrelvir) deployment beginning in late 2021. Reference [107] demonstrated that in vitro nirmatrelvir resistance mutations remained rare in the GISAID database post-EUA, and [108] showed that resistance mutations are invariably accompanied by decreased catalytic activity, explaining why purifying selection intensifies rather than diversifying selection emerging in response to drug pressure (Figure 5a).

Consistent with previously reported values, Spike diversifying escalation matches [19], which reported RBD diversifying sites increasing from 3.6% to 8.1% to 13.1% across their three eras. Our absolute percentages are lower because we analyze the full 1273-codon Spike rather than the 223-codon RBD alone, and the trajectories are parallel only in part: theirs rises monotonically across all three eras, whereas ours peaks in Phase 2 and retreats slightly in Phase 3. The ORF8 spike-then-crash is consistent with [103], which used Washington State genomic surveillance to show that ORF8 nonsense mutations resulting in shortened protein products occur more frequently and are associated with faster clade growth rates than synonymous mutations—direct evidence of positive selection for ORF8 knockout. The NSP5 purifying deepening is consistent with [109], which reported E166V (the primary nirmatrelvir resistance mutation) appeared only 46 times in 15,634,584 GISAID sequences (~0.00029%), confirming extreme rarity of functional NSP5 mutations despite widespread Paxlovid use.

While the literature consistently reports NSP5 under purifying selection [18,104], the temporal intensification from 1.60% to 1.93% across pandemic phases has not been previously documented at the FUBAR site level. This finding has direct implications for antiviral drug development: it suggests that the mutational space available to NSP5 is shrinking over time, making nirmatrelvir resistance increasingly difficult to achieve—a favorable prognosis for the continued efficacy of Paxlovid-class protease inhibitors.

#### 3.5.3. Geographic Differences Across States

To address whether evolutionary dynamics differed by region, genome-wide mutation rates were compared across the ten states grouped into coastal (California, Washington, New York, Massachusetts, Florida, North Carolina) and inland/continental (Texas, Minnesota, Colorado, Illinois; Texas is assigned here on the basis of its predominantly inland population distribution despite its Gulf coastline) populations. The dominant pattern was striking synchrony: all ten states exhibited monotonically increasing mutation rates across the three phases, converging into a narrow Phase 3 band (0.133–0.178), and regional averages were nearly identical in Phase 3 (coastal 0.150 versus interior 0.147). This cross-regional convergence indicates that national-scale immune pressure, rather than local demographic or geographic factors, was the principal driver of mutation-rate evolution. Within this synchrony, modest but consistent regional texture was observed: coastal states showed marginally steeper Phase 1-to-Phase 3 fold increases than interior states (mean 3.13-fold versus 2.85-fold), driven by major coastal transmission hubs. New York reached the highest Phase 3 mutation rate of any state (0.178, a 3.58-fold increase), and Washington showed the largest fold increase overall (3.95-fold), consistent with their roles as dense, immunologically heterogeneous gateway populations, while Florida was a coastal exception with the lowest Phase 3 rate (0.133). These results indicate that although the trajectory of SARS-CoV-2 mutation-rate evolution was nationally coherent, high-density coastal hubs experienced a modestly accelerated pace, an observation that warrants parameter-wide regional analysis in future work.

#### 3.5.4. Convergent Selection at Spike Codon Positions

Analysis of individual Spike codon positions across all 10 US states identified 36 positions with convergence frequency ≥ 0.3 (detected in ≥3 states in at least one phase) (Figure 6). Convergence frequency—the fraction of geographically distinct populations detecting a position as diversifying—is a novel metric enabled by our multi-location study design that provides stronger evidence for immune-driven selection than single-population analyses, subject to the shared-lineage caveat set out in Section 2.3.2.

In Phase 2, position 138 achieved 90% convergence (9/10 states), position 501 (N501Y) reached 90%, and position 681 (P681H/R) reached 90%. These correspond to modification of the spike N-terminal domain immediately adjacent to the antigenic supersite β-hairpin (138), ACE2 binding enhancement (501), and furin cleavage site optimization (681)—all well-characterized Alpha/Delta adaptive mutations.

In Phase 3, position 142 achieved 100% convergence (10/10 states)—the highest convergence observed for any position in any phase. Position 484 (E484K/A) reached 70%, and positions 452 (L452R) and 478 (T478K) each reached 60%. These are Omicron-era immune escape sites. Position 5 (L5F, signal peptide) maintained 80% convergence across all three phases, representing a persistently advantageous mutation not driven by antibody escape; its location within the signal peptide suggests a role in signal peptide processing or expression efficiency, which is proposed here as a hypothesis rather than established. The biological significance of these convergent positions lies in their function: 484, 452, and 478 are principal antibody-escape and ACE2-modulating residues, and their recurrence in six to seven of ten demographically distinct state populations—against a single position, 142, reaching all ten—indicates selection that is strongly reproducible and immune-driven rather than stochastic, while falling short of complete geographic penetrance. The contribution here is therefore not the identification of these positions per se, but the demonstration that immune-escape convergence at functionally critical sites is reproducible across geographically distinct populations and intensifies under sustained immune pressure, indicating predictable, convergent routes of immune escape relevant to vaccine-strain selection.

Texas Phase 3 exhibited the highest single-state diversifying site count: 30 sites (2.36% of Spike codons), consistent with the high variant diversity in a large, immunologically heterogeneous population.

All major convergent positions are independently validated: Position 484 (E484K/A)—class II antibody escape, convergent across Beta, Gamma, Zeta, Kappa, and Omicron [26]. Position 501 (N501Y)—ACE2 binding enhancement, convergent across Alpha, Beta, Gamma [22]. Position 452 (L452R)—class III antibody escape, convergent across Delta and Epsilon [110] and recurring in later Omicron sublineages [27]. Position 681 (P681H/R)—furin cleavage optimization, convergent in Alpha (P681H) and Delta (P681R) [111]. Position 142—NTD antigenic supersite, recurrent deletions/substitutions [112].

The detection of these positions across 6–10 US state populations represents geographic replication of known immune escape signals. Reference [19] showed 12 key positions from a single global dataset; our 36 convergent positions across 10 geographically distinct populations provide broader coverage and demonstrate reproducibility across sampling frames rather than universality of selection. The 24 additional positions include S2 sites (950, 1118, 1191) and NTD sites (18, 19, 26, 80) not highlighted by Gayvert, expanding the known catalog of convergently selected Spike positions.

#### 3.5.5. Domain-Level Selection Mapping

Classification of diversifying Spike positions into functional domains (NTD: residues 13–305; RBD: 319–541; S1/S2: 542–685; S2: 686–1273; boundaries as used throughout this work, with residues 1–12 assigned to the signal peptide and 306–318 to the NTD–RBD linker) revealed a significant temporal shift (Figure 7). Domain proportions are computed by pooling all diversifying positions detected across the ten states within each phase, so each position contributes once per state in which it was detected. An “Other” category (positions 1–12 and 306–318, including the signal peptide and inter-domain linker) accounted for 12% in Phase 1, declining to 4–5% in later phases. In Phase 1, diversifying selection was broadly distributed: NTD 26%, RBD 12%, S1/S2 25%, S2 25%. By Phase 3, selection concentrated in NTD (38%) and RBD (37%), while S2 dropped to 9% and S1/S2 to 11%.

The RBD proportion tripled from 12% to 37% between Phase 1 and Phase 3, coinciding with the Omicron era when RBD mutations became the primary mechanism of immune escape. The S2 decline from 25% to 9% reflects conservation of the fusion machinery essential for viral entry—the virus cannot tolerate mutations in this domain even as NTD and RBD undergo rapid antigenic evolution.

Consistent with previously reported values, the RBD proportion tripling is consistent with [26], which demonstrated that Omicron evolution is driven by imprinted humoral immunity inducing convergent RBD evolution, and with [113], showing post-Omicron antibody escape mutations concentrate at RBD epitope positions; Omicron’s combination of increased infectivity, vaccine breakthrough, and antibody resistance has been characterized in detail [114]. S2 decline is consistent with conservation of S2 fusion machinery across all variants of concern [22]. The [115] study independently mapped FUBAR-identified sites to Spike domains, reporting NTD and RBD enrichment in Omicron subvariants.

While previous studies [19,115] identified NTD and RBD enrichment in aggregate global datasets, this analysis provides quantitative evidence of geographic universality in the Spike domain selection shift. The exceptionally tight clustering of individual state proportions across all 10 US states demonstrates that the evolutionary pivot away from the S2 domain toward the RBD is a highly reproducible response to immune pressure rather than a localized sampling artifact. The geographic reproducibility documented here adds a layer of geographic replication evidence not available in single-dataset analyses.

### 3.6. Spike Protein Mutation Profile (Parameter 5)

The Spike protein, as the primary target of all COVID-19 vaccines and neutralizing antibodies, underwent the most functionally consequential mutations during the pandemic. This parameter quantifies the accumulation of amino acid changes across the full 1273-residue Spike protein and tracks 14 key mutations with documented functional significance for immune escape, receptor binding, and transmissibility across 10 US states.

#### 3.6.1. Spike Mutation Burden

Population-averaged Spike mutation burden increased progressively across the three pandemic phases: 1.47 ± 0.26 (Phase 1), 6.17 ± 0.75 (Phase 2), and 9.46 ± 2.11 (Phase 3) mutations per sequence, representing a 6.5-fold increase (Figure 8a). The Phase 1 baseline reflects the pre-vaccination era when minimal immune selection pressure existed. The Phase 2 escalation coincides with the emergence of Alpha (B.1.1.7) and early Delta variants, each carrying 6–9 defining Spike mutations. Phase 3 values reflect the sustained immune pressure era, in which the volume-weighted sample is dominated by late-stage Delta with an early Omicron contribution; Omicron sublineages carry 30+ Spike mutations relative to Wuhan-Hu-1. Individual triplicate values (black dots) demonstrate tight clustering around phase means, confirming the reproducibility of the random sampling methodology.

Consistent with previously reported values, this trajectory matches Carabelli et al. [22], who documented progressive Spike mutation accumulation from ancestral (≈0 changes) through Alpha (≈9) to Omicron (≈30). The population-averaged values reported here are lower than variant-defining counts because they average all circulating lineages at each time point, providing a more epidemiologically representative metric than variant-specific counts.

#### 3.6.2. Key Mutation Frequency Dynamics

Fourteen of the tracked substitutions, each with documented functional significance, are reported here across all locations and phases (Figure 8b). D614G, the first major Spike mutation, achieved near-fixation (94.9% → 100.0% → 99.9%), consistent with Korber et al. [56], who demonstrated its enhanced transmissibility through increased virion Spike density. N501Y surged during Phase 2 (54.1%), driven by Alpha’s defining RBD substitution, then declined to a Phase 3 average of 9.3%, reflecting sample composition rather than reversion: N501Y is carried by all Omicron lineages, whereas Delta lacks it entirely. Because the volume-weighted Phase 3 sample is approximately 90% Delta and only some 8–9% Omicron (Parameter 10), the phase-level mean falls close to the Omicron fraction itself. The E484 position shifted from E484K (11.1% in Phase 2, Beta/Gamma-associated) to E484A (8.1% in Phase 3, Omicron-specific), tracking lineage turnover while illustrating repeated independent substitution at the same residue across distinct variant backgrounds, as described by Cao et al. [26]. The P681 furin cleavage position underwent a functional switch from P681H (52.7%, Alpha-driven) to P681R (90.7%, Delta-driven; Omicron instead carries P681H), reflecting the cleavage efficiency advantage documented by Johnson et al. [69]. L452R and T478K both reached high Phase 3 frequencies (91.1% and 98.4%). Because Delta carries both substitutions and constitutes approximately 90% of the volume-weighted Phase 3 sample, their joint frequency primarily reflects linkage on a shared lineage background rather than independent convergent selection. T478K is the higher of the two because Omicron lineages also carry it, whereas BA.1 and BA.2 lack L452R.

#### 3.6.3. Mutation Frequency Heatmap

The geographic mutation frequency heatmap (Figure 8c) reveals the spatial heterogeneity of mutation profiles across 10 US states. Phase 1 shows minimal mutation presence beyond D614G, confirming the pre-vaccination evolutionary quiescence. Phase 2 displays characteristic variant signatures: N501Y and P681H from Alpha spread asynchronously across locations, while L452R shows location-specific enrichment reflecting local variant epidemiology. By Phase 3, the dominant Delta markers (L452R, T478K, P681R) have converged to near-uniform high frequencies across all locations, consistent with the volume-weighted Phase 3 sample being approximately 90% Delta; of the three, only T478K is also carried by the early Omicron lineages present in the sample. Subsidiary mutations retain location-specific signatures reflecting the differing early Omicron fractions across locations. Domain annotation squares (red = RBD, green = Furin, blue = SD2, purple = HR1) highlight that 10 of 14 tracked mutations are in the RBD, consistent with this domain being the primary target of immune-driven selection.

#### 3.6.4. Phase-Averaged Frequency Shift

Phase-averaged frequency shift, quantified as percentage points per month (pp/mo), captures the mean rate of frequency change across each phase transition (Figure 8d). It is important to note that this metric is subject to a dilution artifact: because Phase 3 spans 54 months compared to Phase 2’s 6 months, rapid Omicron sweeps that occurred over weeks are divided by the full phase window, numerically suppressing Phase 3 velocities relative to their true biological speed. This metric should therefore be interpreted as phase-normalized frequency shift rather than instantaneous spread velocity. N501Y showed a phase-averaged shift of 5.98 pp/mo during the Alpha/Delta era, consistent with Alpha’s estimated 40–80% transmissibility advantage (Davies et al. [12]). Per-location velocity dots demonstrate geographic variance in spread rates, reflecting asynchronous variant introduction. Phase 3 frequency shifts appear numerically lower in absolute pp/mo, a direct consequence of the 54-month Phase 3 window diluting rapid Omicron sweeps that biologically occurred over 2–4 months. This metric provides a standardized, coefficient-of-variation-based, geographically replicated measurement of mutation evolutionary convergence across states.

#### 3.6.5. Geographic Convergence Index

The Geographic Convergence Index, measured as the coefficient of variation (CV = SD/Mean) across locations over time, quantifies whether states are converging toward uniform mutation profiles (Figure 8e). L452R CV declined from 2.63 (Phase 1) to 0.10 (Phase 3). The declining CV trajectories for D614G, L452R, T478K, and P681R are consistent with immune-driven selection producing convergent outcomes across states with different starting variant compositions. D614G serves as a positive control with CV near zero across all phases, consistent with a mutation that reached near-fixation during Phase 1—its Phase 1 frequency here is 94.93%—showing minimal inter-location variance. The 95% bootstrap confidence bands narrow dramatically by Phase 3, confirming convergence with high statistical confidence. This metric quantifies the rate at which states converge to uniform mutation frequencies. As stated in Section 2.3, the ten states are separate sampling frames within a shared national epidemic rather than evolutionarily independent replicates.

In summary, this parameter provides: (1) Population-averaged Spike mutation burden as an epidemiologically representative metric, distinct from variant-defining mutation counts; (2) The E484K→E484A frequency transition documenting convergent evolution at the residue level across states; (3) P681H→P681R intersecting frequency curves quantifying the population-level variant succession at the furin cleavage site; (4) Mutation spread velocity (pp/mo) as a standardized metric with geographic replication; (5) The Geographic Convergence Index providing quantitative evidence that immune-driven selection produces uniform mutation outcomes across states.

### 3.7. Insertion–Deletion (Indel) Analysis (Parameter 6)

Insertions and deletions (indels) are fundamentally distinct from point mutations: deletions physically remove protein segments and insertions add new ones, making them powerful tools for immune evasion by ablating antibody binding sites entirely rather than merely altering them. This parameter quantifies indel prevalence, tracks five functionally significant deletions, maps deletion hotspots across the genome, and characterizes gene-level deletion distribution across 10 US states.

#### 3.7.1. Indel Prevalence

The proportion of sequences carrying at least one deletion increased dramatically across the three pandemic phases: 4.77% ± 1.65 (Phase 1), 70.17% ± 12.75 (Phase 2), and 99.54% ± 0.68 (Phase 3), representing a 20.9× increase and near-complete universalization of deletions by Phase 3 (Figure 9a). Insertions remained rare throughout: 0.4% ± 0.4 (Phase 1), 6.2% ± 4.8 (Phase 2), and 1.9% ± 1.1 (Phase 3), confirming that the primary indel-driven evolutionary mechanism is deletion, not insertion. The Phase 1 baseline reflects the ancestral Wuhan-Hu-1 lineage, which carried no defining deletions. The Phase 2 surge coincides with Alpha (B.1.1.7), which introduced Δ69–70 and Δ144 as lineage-defining deletions. By Phase 3, the successive dominance of Delta (carrying its own defining NTD lesion, E156G with Δ157/158) followed by Omicron and its highly deleted sublineages made structural deletions a universal feature of circulating SARS-CoV-2.

Consistent with previously reported values, Rogozin et al. [116] analyzed 633,995 SARS-CoV-2 genomes and identified 1229 deletion events (639 short and 590 long), reporting a consistent excess of in-frame over out-of-frame deletions. Deletions likewise dominate here, with 267,733 deletion occurrences against 4390 insertion occurrences. The two counts are not directly comparable. Rogozin et al. report inferred deletion events on a phylogeny, whereas the present figures are per-sequence occurrences within the sampled cohort. McCarthy et al. [62] identified that >97% of NTD deletions preserve the reading frame, consistent with positive selection rather than random drift.

Consistent with previously reported values, Akaishi et al. [117] reported that 7 of 13 preserved indel patterns were Omicron-specific, consistent with our Phase 3 universalization driven by Omicron sublineages.

#### 3.7.2. Key Deletion Frequency Dynamics

Five deletions with documented functional significance were tracked across all locations and phases; four are shown in Figure 9b, for the reason given below. Δ69–70 (HV69–70del), located in the spike N-terminal domain adjacent to—but outside—the antigenic supersite loops (N1 residues 14–26, N3 141–156 and N5 246–260 [112]), surged from 0.7% (Phase 1) to 47.2% (Phase 2) with Alpha, then declined to 1.6% (Phase 3) because the Delta variant—which dominated the first six months of Phase 3 (July–December 2021) in the US—entirely lacks this deletion, followed by the later dominance of Omicron BA.2 and XBB sublineages which also lack Δ69–70. Δ144 (Y144del) followed an identical trajectory: 0.4% → 48.3% → 1.3%. Both deletions co-occur in Alpha, reflecting coordinated immune evasion at the NTD epitope surface. The parallel rise and fall demonstrates that these deletions are functionally linked through Alpha lineage dynamics. Δ211 (N211del) appeared only in Phase 3 at 0.4%, consistent with its Omicron BA.1-specific origin. Δ242–244 (Beta-defining) remained at trace levels, reflecting the extremely limited circulation of Beta (B.1.351) in US populations. Δ25–27 ORF8 was returned at essentially zero frequency, but this value should not be interpreted biologically. The detection window implemented for this deletion spans a wider ORF8 interval than the three intended codons, and the 50% reciprocal-overlap criterion used to call a named deletion therefore requires a substantially larger ORF8 deletion than Δ25–27 itself to register. The near-zero frequency reported here reflects that detection window rather than a measured absence. Δ25–27 has therefore been withdrawn from Figure 9b entirely rather than shown at a frequency that could be misread as biological absence, and it is excluded from interpretation; the four spike deletions above are unaffected, their windows matching the intended codons exactly.

Consistent with previously reported values, McCarthy et al. [62] defined four recurrent deletion regions (RDR1: Δ69–70, RDR2: Δ144/145, RDR3: Δ210, RDR4: Δ243–244) and demonstrated Δ69–70 arose independently at least 13 times. Our temporal tracking adds population-level epidemiological context, showing 47.2% peak prevalence.

Consistent with previously reported values, Carabelli et al. [22] noted NTD deletions repeatedly altered antigenicity across variants. Parsons and Acharya (2023) [118] reported that Δ144 led to increased immune evasion while Δ69–70 alone did not substantially alter serum antibody recognition, consistent with their co-occurrence in Alpha as a coordinated escape strategy.

Consistent with previously reported values, Alisoltani et al. [119] identified Δ69–70 and Δ144 as occurring in NTD hypervariable regions HVR1 and HVR2, respectively, confirming these as recurrent deletion regions under adaptive selection. Akaishi et al. [120] mapped NTD indel hotspots to genome positions 21,770–21,820 and 21,915–22,037, both on the exposed surface of the spike NTD.

Here, Δ69–70 and Δ144 are tracked as population frequency curves with per-location geographic variance across 10 US states. The coordinated rise–peak–decline pattern provides the epidemiological quantification of NTD deletion dynamics at the population level.

#### 3.7.3. Deletion Hotspot Genome Map

Genome-wide mapping of deletion positions revealed five dominant hotspot regions: Spike (n = 99,740 cumulative deletion events across all sequences), Intergenic (n = 72,761), ORF8 (n = 45,926), NSP6 (n = 34,829), N (n = 3801) (Figure 9c). Phase 1 deletions were few in absolute number and spread across several regions, with Spike already the leading target (312 events) ahead of NSP1 (298) and the 3’UTR (171). Phase 2 introduced concentrated Spike NTD deletions at positions 21,764 (Δ69–70) and 21,990 (Δ144). By Phase 3, Spike deletions dominated the landscape with additional NSP6 and ORF8 contributions. The Intergenic region accumulated deletions primarily at position 28,270 (between ORF8 and N), a defining feature of Alpha and Delta. Critically, replication machinery genes (NSP7–NSP16, including RNA-dependent RNA polymerase NSP12 and helicase NSP13) showed virtually zero deletions across all phases—direct evidence for differential selection pressures: positive selection at immune-facing surfaces and purifying selection at functional core proteins.

Consistent with previously reported values, Akaishi et al. [120] mapped indel hotspots to Spike NTD (positions 21,500–22,500) and ORF8 (27,800–28,200). Our genome map confirms both hotspots with population-level data and temporal resolution.

Consistent with previously reported values, Rogozin et al. [116] reported a dramatic excess of deletions in ORF7a and ORF8 relative to the rest of the genome, with ORF6 and ORF7b also carrying high deletion densities for their length—all accessory genes involved in host immune interactions. Wagner et al. [103] demonstrated positive selection for ORF8 knockout, consistent with ORF8 being our third-largest deletion target.

Previous studies mapped deletion hotspots cross-sectionally. Our three-panel approach shows how the deletion landscape evolves temporally across phases, demonstrating the emergence and intensification of hotspots under increasing immune pressure with geographic replication across 10 US states.

#### 3.7.4. Gene-Level Deletion Distribution

Gene-level aggregation confirmed Spike as the dominant deletion target across all three phases, with deletion counts increasing dramatically from Phase 1 to Phase 3 (Figure 9d). ORF8 and NSP6 emerged as secondary hotspots primarily in Phases 2 and 3. The concentration of deletions in genes involved in immune modulation (Spike, ORF8) and autophagy regulation (NSP6), combined with the near-absence of deletions in replication genes (NSP7–NSP16), demonstrates that indels are under strong positive selection at immune-facing surfaces and strong purifying selection at functional core proteins. Importantly, the concentration of deletions in ORF8 is not in tension with the Phase 3 decline in ORF8 diversifying selection (Section 3.5.2, Figure 5b); rather, both reflect the same trajectory of progressive ORF8 loss of function. The positive selection acting on ORF8 indels is selection for loss-of-function deletions and truncations that dismantle a now-dispensable accessory protein, not selection to refine or maintain it. This interpretation is consistent with reports that ORF8 knockout variants (e.g., premature stop codons and large ORF7b-ORF8 deletions) recur across dominant lineages and are associated with a fitness advantage in the Omicron era, such that the deletion signature in ORF8 represents the molecular mechanism by which its evolutionary abandonment occurs.

Beyond reconciling these observations, the present analysis contributes corroboration-at-scale with temporal resolution: the ORF8 spike-then-collapse trajectory is independently reproduced across ten demographically distinct states and resolved across three defined pandemic phases using a triplicate design. We do not claim first discovery of ORF8 functional loss; rather, we provide reproducible, geographically replicated, phase-resolved quantification of its trajectory, which strengthens confidence that this is a general feature of SARS-CoV-2 adaptation rather than a region-specific artifact.

Consistent with previously reported values, Rogozin et al. [116] detected only a few long deletions in ORF1ab, which encodes NSP1–NSP16, as well as in E, M, and N. Our gene-level distribution resolves this to the individual NSP level, showing zero or near-zero deletions across NSP7–NSP16 in all three phases and all 10 US states.

The temporal emergence pattern—minimal Phase 1, concentrated Phase 2, universal Phase 3—quantifies how deletion selection intensified with increasing immune pressure. Geographic replication across 10 US states confirms this is not a sampling artifact.

In summary, this parameter provides: (1) Population-level indel prevalence tracking demonstrating deletion universalization (4.77% → 99.54%, a 20.9× increase) across 10 US states with triplicate design; (2) Temporal tracking of the four spike deletions (Δ69–70, Δ144, Δ211, Δ242–244) as population frequency curves with geographic variance; (3) Phase-resolved genome-wide deletion hotspot mapping confirming the immune-facing vs. replication-machinery selection dichotomy; (4) Gene-level deletion distribution with temporal resolution showing deletion selection intensification under increasing immune pressure.

### 3.8. ACE2 Receptor-Binding Domain Contact Residue Analysis (Parameter 7)

The receptor-binding domain (RBD) of the SARS-CoV-2 spike protein engages human ACE2 through an extensive receptor-binding motif interface critical for viral entry. Mutations at these positions directly modulate receptor-binding affinity and can simultaneously alter antibody recognition, creating a dual selection pressure that drives adaptive evolution. This parameter tracks mutation frequency at the 17-position receptor-binding motif panel defined in Section 1.3.7, which combines structurally defined ACE2 contacts with established immune-escape positions (K417, G446, Y449, Y453, L455, F456, A475, S477, E484, F490, Q493, G496, Q498, T500, N501, G502, Y505) across three pandemic phases for 10 US states.

#### 3.8.1. ACE2 Contact Mutation Burden (Figure 10a)

The mean number of ACE2 contact residue mutations per sequence increased 34.9-fold from Phase 1 to Phase 2: 0.02115 ± 0.02011 (Phase 1) to 0.73878 ± 0.18073 (Phase 2) to 0.61434 ± 0.67138 (Phase 3). The Phase 1 near-zero baseline (0.021) reflects the Wuhan-Hu-1 lineage, which carried no defining ACE2 contact mutations. The Phase 2 surge to 0.739 coincides with Alpha (B.1.1.7), which introduced N501Y as the first ACE2 contact mutation fixed in a variant of concern. The apparent decline from Phase 2 to Phase 3 is primarily a Delta dilution artifact: the Delta variant (dominating Phase 3 from July–December 2021) carried virtually no mutations at these 17 core ACE2 contact residues, driving the phase-level mean downward despite Omicron sublineages (BA.1–BA.3) carrying 7–10 simultaneous contact mutations. The large Phase 3 SD (0.671, exceeding the mean of 0.614) additionally reflects extreme temporal and geographic heterogeneity: US states with heavier 2021 sequencing capture more Delta-dominated sequences, while those with sustained 2022–2025 surveillance capture Omicron-rich samples. This is consistent with Mannar et al. [121], who demonstrated via cryo-EM that Omicron BA.1 carries 10 mutations at the ACE2 interface, forming new salt bridges and hydrogen bonds at R493, S496, and R498 that compensate for K417N. Our population-level burden metric provides the epidemiological quantification of this structural observation.

**Figure 10:**
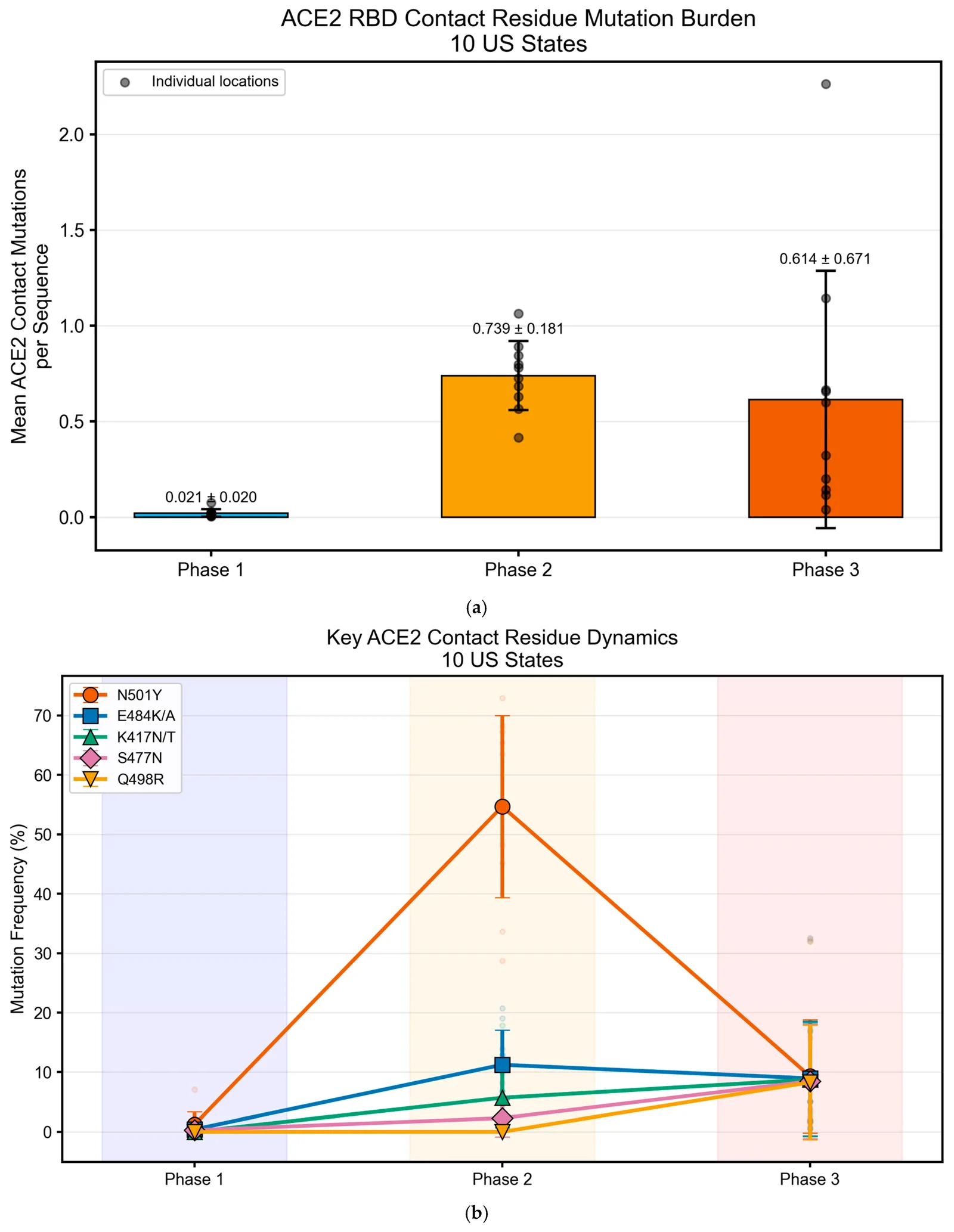

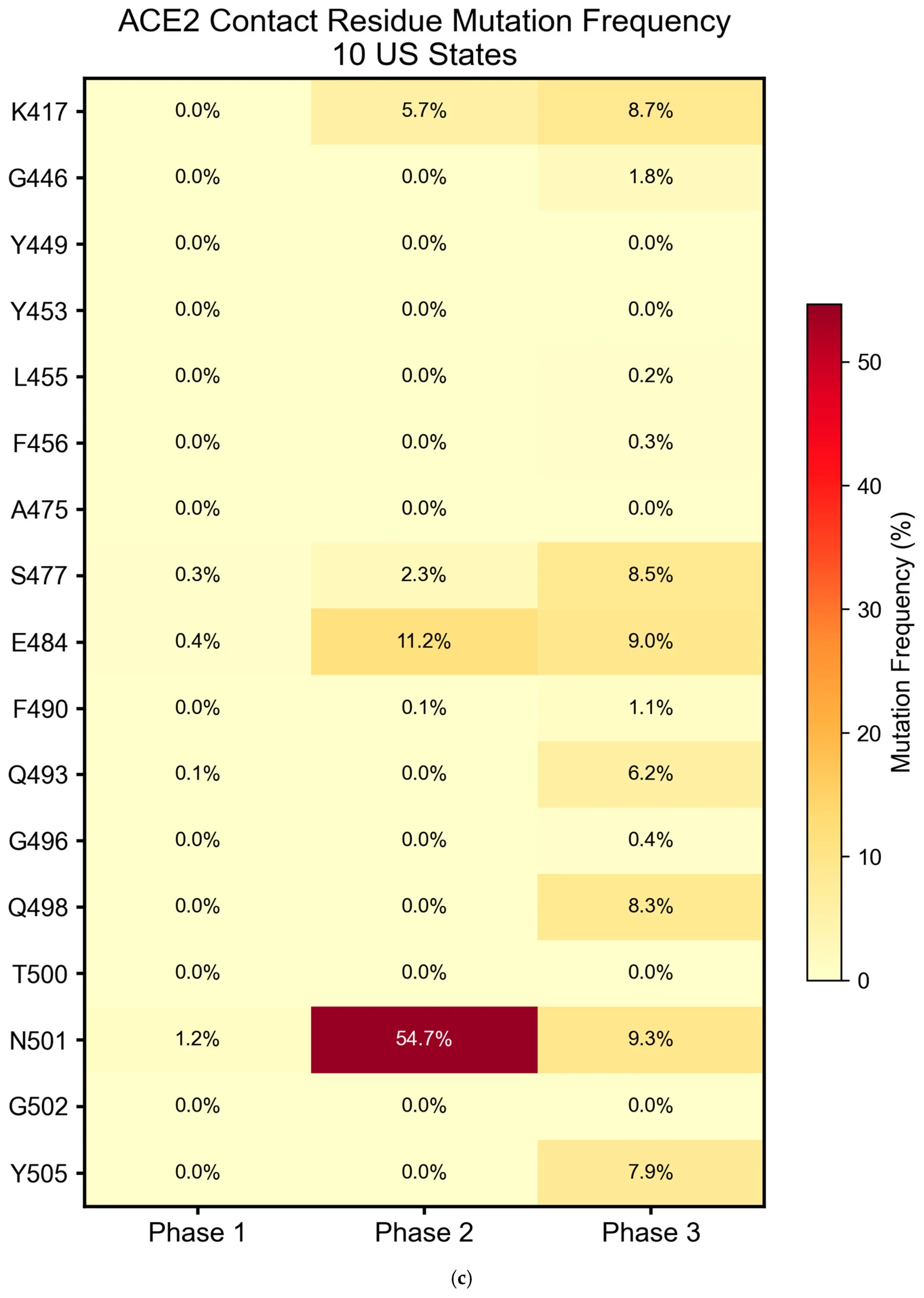

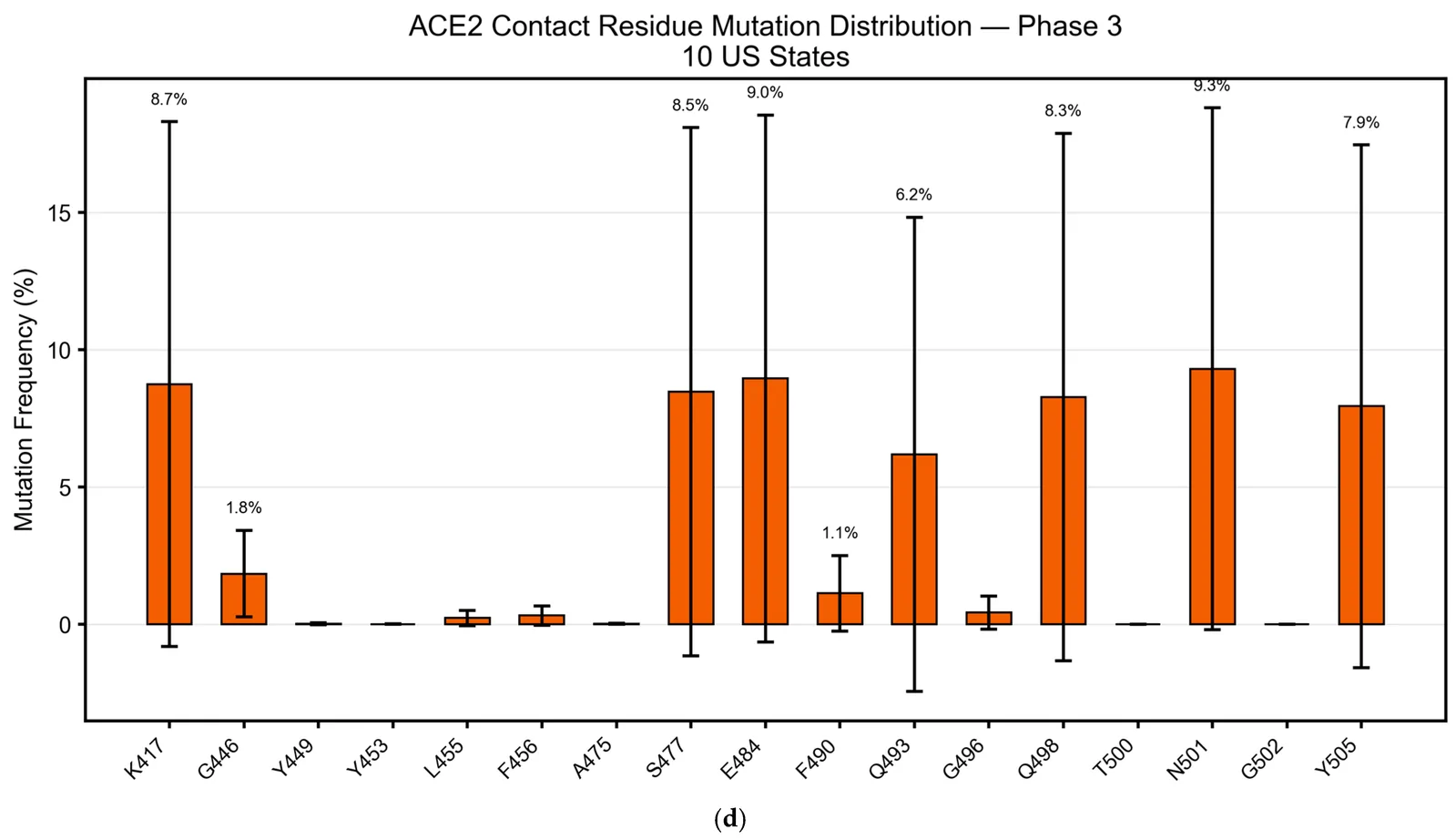
(**a**) ACE2 RBD contact residue mutation burden across three pandemic phases for 10 US states. Bars = cross-location mean. Black dots = individual location means. Error bars = ± SD. (**b**) Key ACE2 contact residue dynamics across 10 US states. Bold symbols = cross-location mean. Faint dots = individual location values. Error bars = ± SD. (**c**) ACE2 contact residue mutation frequency heatmap across 10 US states. Rows = 17 ACE2 contact positions. Columns = three pandemic phases. Color intensity = mean mutation frequency (%). (**d**) Phase 3 ACE2 contact residue mutation distribution across 10 US states. Bar height = cross-location mean mutation frequency. Error bars = ± SD.

#### 3.8.2. Key ACE2 Contact Residue Frequency Dynamics (Figure 10b)

Five ACE2 contact residues with documented functional significance were tracked across all phases. N501Y surged from 1.2% ± 2.2% (Phase 1) to 54.7% ± 15.3% (Phase 2), then declined to 9.3% ± 9.5% (Phase 3), primarily reflecting sample composition: Delta carries the wild-type residue at position 501 and constitutes approximately 90% of the volume-weighted Phase 3 sample, whereas N501Y is retained across Omicron lineages, which contribute only some 8–9% (Parameter 10). This is consistent with Xue et al. [122], who describe N501Y as a key epistatic mutation that compensates for the affinity losses caused by antibody-escape substitutions, maintaining overall ACE2 affinity across the major variants. E484K/A increased from 0.4% ± 0.5% to 11.2% ± 5.8% to 9.0% ± 9.6%, reflecting the transition from E484K (Beta/Gamma immune evasion) to E484A (Omicron). K417N/T was absent in Phase 1 (0.0%), rose to 5.7% ± 5.0% with Beta/Gamma, then increased to 8.7% ± 9.6% as K417N became fixed in all Omicron sublineages. Despite reducing ACE2 affinity alone, K417N is compensated by Q493R and Q498R in Omicron [121]. S477N showed a progressive increase (0.3% → 2.3% → 8.5%), becoming fixed in all Omicron sublineages, which display broadly comparable ACE2 binding affinities despite their divergent RBD mutation profiles [123]. Q498R was effectively confined to Phase 3 (8.3% ± 9.6%; a single Phase 1 sequence aside), forming new salt bridges with ACE2 D38 consistent with the epistatic dependency on N501Y described by Starr et al. [124].

#### 3.8.3. ACE2 Contact Residue Mutation Heatmap (Figure 10c)

Genome-wide mapping of all 17 ACE2 contact residue positions across three phases revealed progressive broadening of the mutation landscape. Phase 1 showed near-zero frequencies across all positions. Phase 2 showed concentrated N501Y dominance (54.7%) with secondary E484K (11.2%) and K417N (5.7%). By Phase 3, 7 of 17 positions exceeded 5% mutation frequency (N501 9.3%, E484 9.0%, K417 8.7%, S477 8.5%, Q498 8.3%, Y505 7.9%, Q493 6.2%), demonstrating the composite Phase 3 mutation landscape. These frequencies (~8–9%) are lower than expected for true Omicron dominance (which would produce 80%+ for these hallmark mutations) because the Delta wave in early Phase 3 contributed sequences with wild-type residues at these positions, diluting the Omicron signal in the phase-level average. This is consistent with Xia et al. [125], who reported that all Omicron sublineages share 8 common RBM mutations, including S477N, E484A, Q493R, Q498R, N501Y, and Y505H. Five positions remained essentially invariant across all phases, with mutation frequencies never exceeding 0.4% at any state or phase (Y449, Y453, A475, T500, G502; G502 was unmutated throughout), indicating strong purifying selection preserving these binding contacts. Published structural studies compare individual variants at the level of static structures; the heatmap presented here instead resolves the same interface positions as population-level frequencies across phases and states (Figure 10c).

#### 3.8.4. Phase 3 ACE2 Contact Residue Distribution (Figure 10d)

Phase 3 distribution confirmed a broadened mutation landscape emerging as early Omicron sublineages entered the Delta-dominated sample. Seven positions carried >5% mutation frequency: N501 (9.3% ± 9.5%), E484 (9.0% ± 9.6%), K417 (8.7% ± 9.6%), S477 (8.5% ± 9.6%), Q498 (8.3% ± 9.6%), Y505 (7.9% ± 9.5%), and Q493 (6.2% ± 8.6%). The near-equal mutation frequencies at the top 5 positions (8.3–9.3%) suggest coordinated multi-residue selection rather than single-position dominance, consistent with Omicron’s compensatory binding mechanism described by Mannar et al. [121] and Wang et al. [126]. The large SDs across all positions reflect extreme geographic heterogeneity in Omicron sublineage composition across US states, documenting geographic variance in ACE2 contact mutation profiles (Figure 10d).

In summary, this parameter provides: (1) Population-level ACE2 contact mutation burden quantification (0.021 → 0.739 → 0.614 mutations per sequence) as an epidemiological metric with a 34.9× fold increase and geographic replication across 10 US states; (2) Temporal frequency trajectories of individual ACE2 contact residues showing the N501Y rise–peak–decline pattern (1.2% → 54.7% → 9.3%) and the transition from single-residue dominance to multi-residue Omicron interface remodeling; (3) Phase-resolved heatmap demonstrating ACE2 binding interface broadening (1 position >5% in Phase 2 vs. 7 positions >5% in Phase 3) with identification of 5 invariant positions (Y449, Y453, A475, T500, G502) under purifying selection; (4) Geographic variance analysis revealing heterogeneous ACE2 contact mutation landscapes across states under different Omicron sublineage pressures.

### 3.9. Furin Cleavage Site Evolution (Parameter 8)

The furin cleavage site (FCS) at the S1/S2 junction of the SARS-CoV-2 spike protein (positions 679–685: NSPRRAR) is the defining feature distinguishing SARS-CoV-2 from SARS-CoV-1. This polybasic insertion enables pre-activation of the spike protein by the ubiquitous host protease furin during viral biogenesis, enhancing membrane fusion and transmissibility [70,71]. Mouse-adapted SARS-CoV-2 strains have been generated for vaccine-efficacy testing [127]. Loss of the FCS attenuates SARS-CoV-2 pathogenesis in animal models (Johnson et al. [69]). Mutations at this site—P681H (Alpha), P681R (Delta), N679K (Omicron)—have been linked to increased transmissibility [12,72], enhanced cell–cell fusion [74], and altered protease utilization [128]. This parameter tracks mutation frequency at all 7 FCS positions across three pandemic phases for 10 US States.

#### 3.9.1. Furin Cleavage Site Mutation Burden (Figure 11a)

The mean number of furin cleavage site mutations per sequence increased continuously: 0.022 ± 0.024 (Phase 1) → 0.578 ± 0.135 (Phase 2) → 1.076 ± 0.097 (Phase 3), representing a 26.7-fold increase from Phase 1 to Phase 2 and a continuously ascending trajectory. The Phase 1 near-zero baseline reflects the Wuhan-Hu-1 NSPRRAR motif. The Phase 2 surge to 0.578 coincides with Alpha (B.1.1.7) introducing P681H. The Phase 3 value of 1.076 with a remarkably small SD (0.097) indicates tight convergence across all 10 US states toward approximately 1 furin site mutation per sequence. This continuously ascending burden contrasts with ACE2 contact mutations (Parameter 7), revealing fundamentally different selection regimes. While immune pressure drives continuous multi-residue exploration at the ACE2 interface, optimal transmissibility at the S1/S2 junction is achieved through specific, singular structural enhancements (P681H/R) flanking the highly conserved polybasic RRAR core. The furin site thus exhibits tight evolutionary convergence (approximately 1 mutation per sequence by Phase 3) in contrast to the ACE2 interface’s broader multi-residue diversification landscape. Lubinski et al. [129] demonstrated that P681R substantially increases furin cleavability beyond P681H. Khatri et al. [130] confirmed that Delta+P681R showed a >3-fold increase in cell entry.

**Figure 11:**
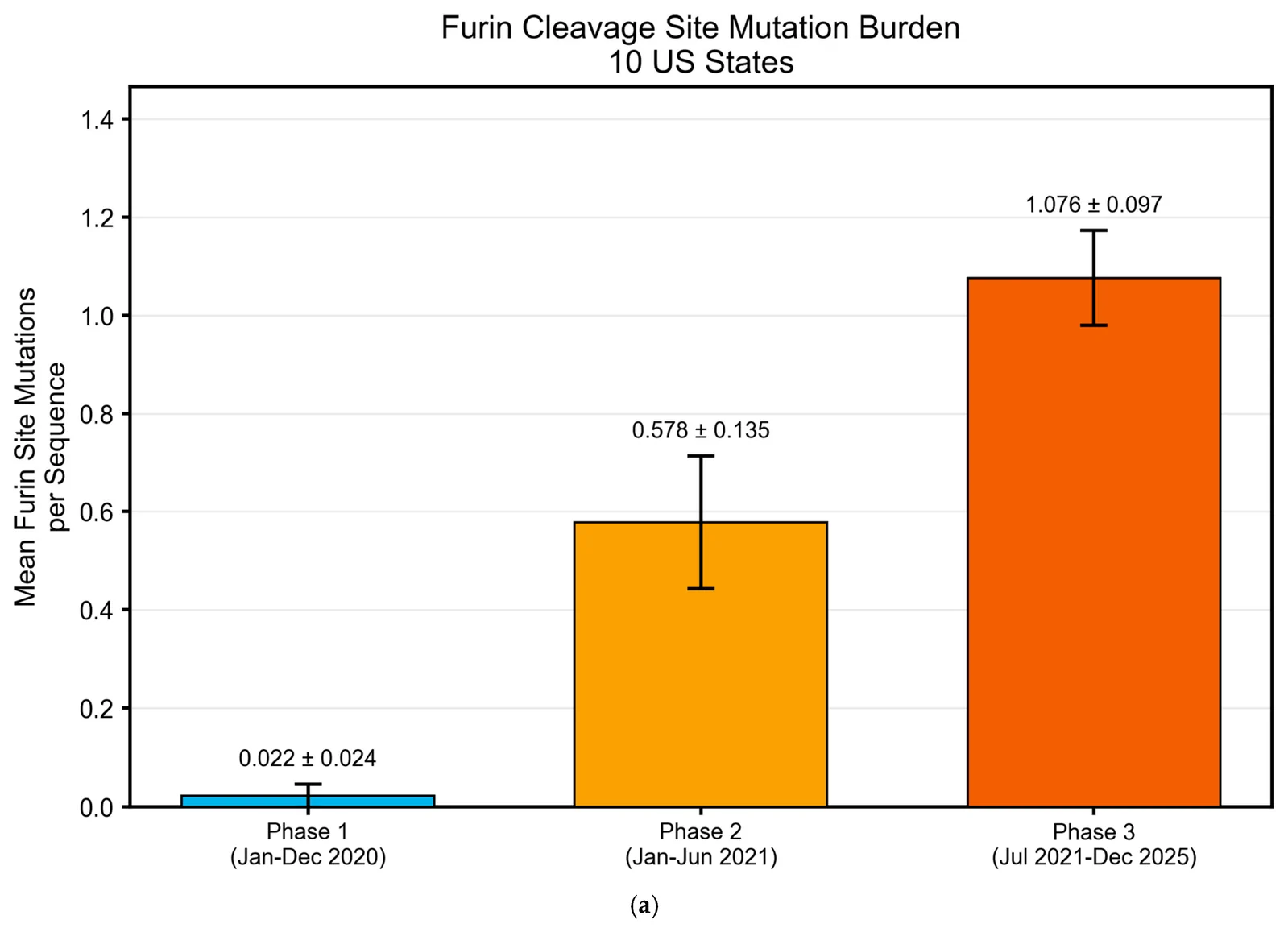

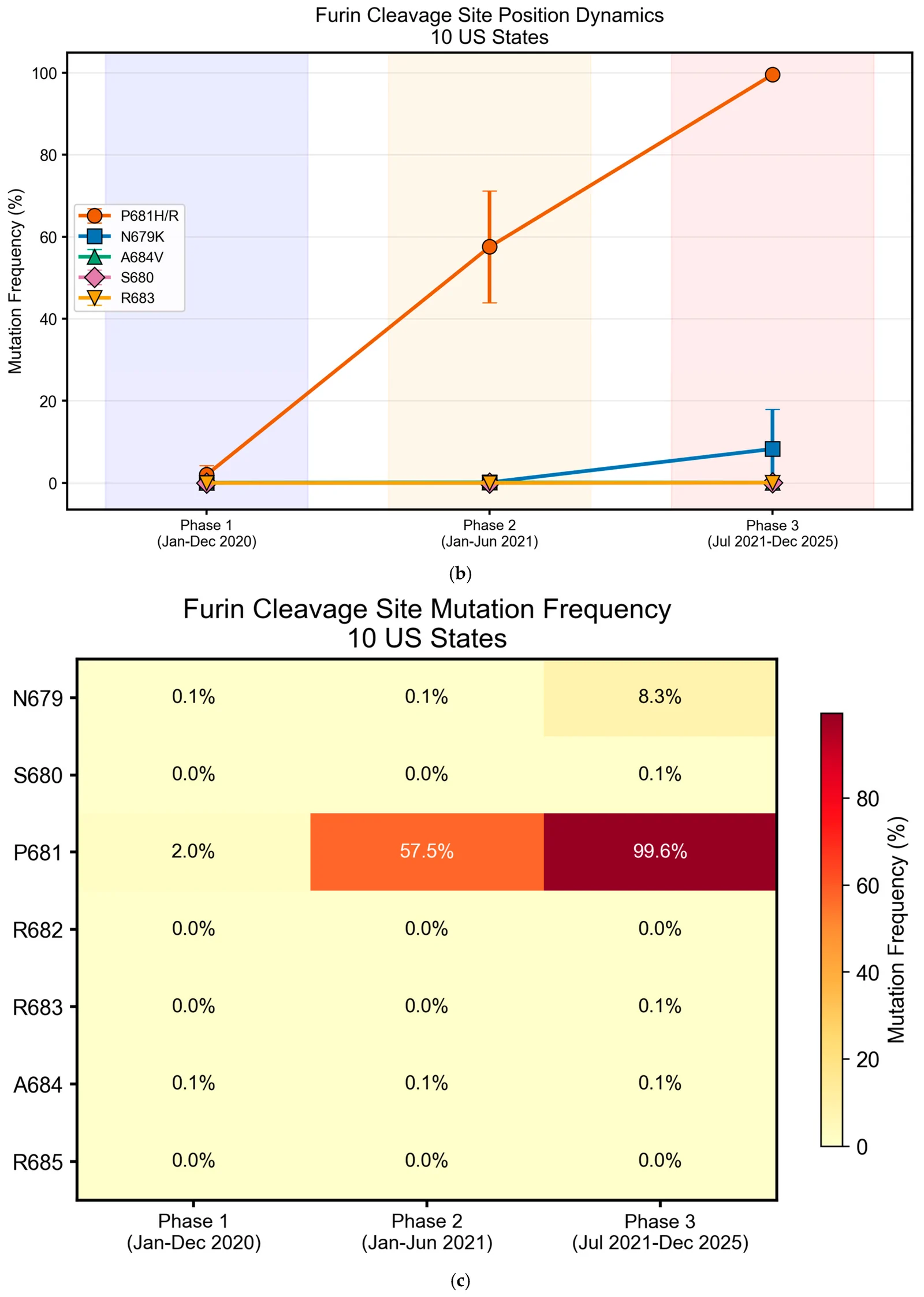

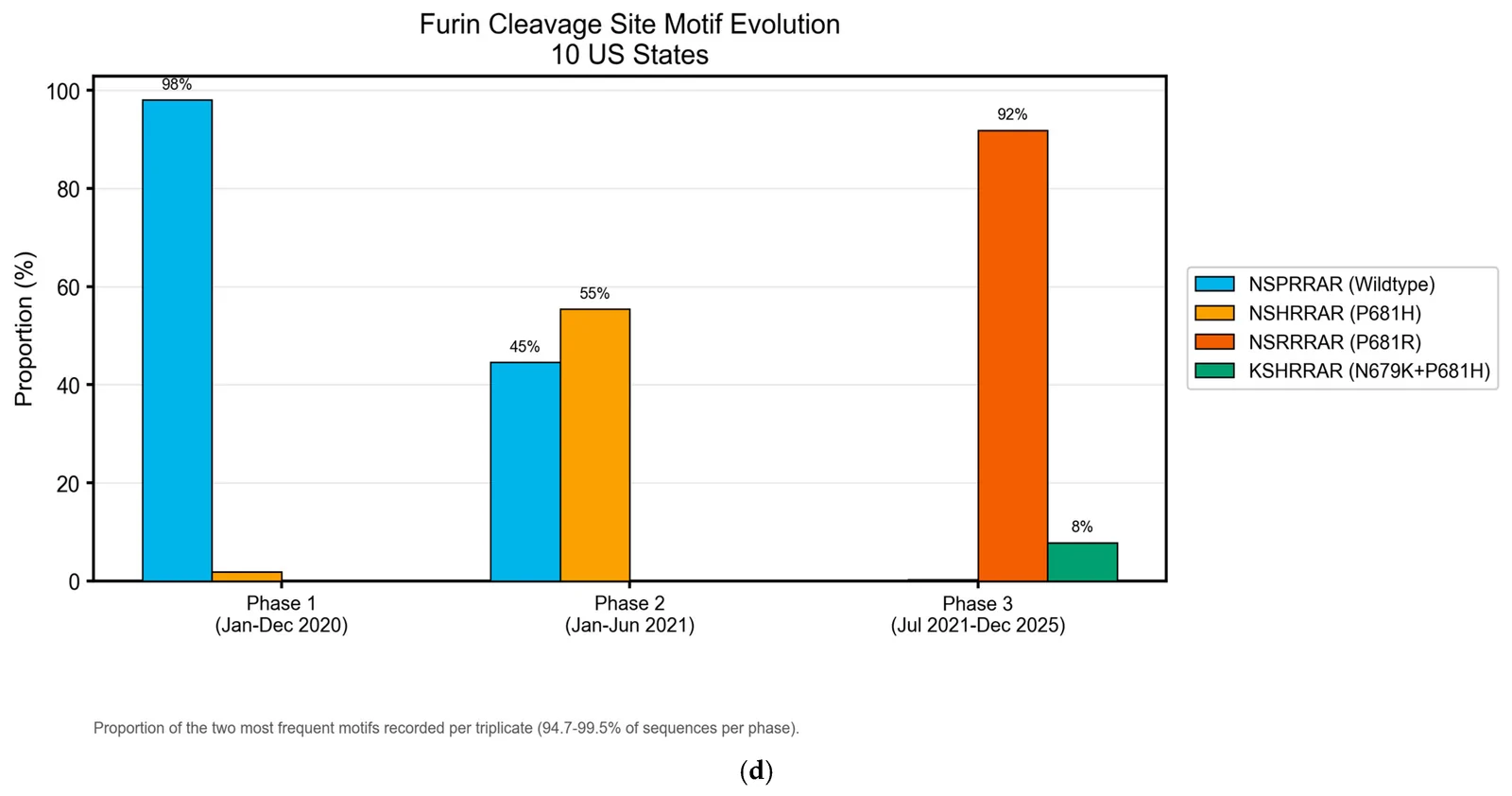
(**a**) Furin cleavage site mutation burden across three pandemic phases for 10 US states. Bar height = cross-location mean mutations per sequence. Error bars = ± SD. Individual location means were computed as triplicate averages but are omitted for clarity. (**b**) Furin cleavage site per-position mutation dynamics across 10 US states. All seven positions (679–685) tracked; 681 and 679 are the only two showing appreciable variation, the remainder staying at or below 0.1% in every phase. Bold symbols = cross-location mean. Error bars = ± SD. Individual location values omitted for clarity. (**c**) Furin cleavage site mutation frequency heatmap across 10 US states. Rows = 7 FCS positions (679–685). Columns = three pandemic phases. Color intensity = mean mutation frequency (%). (**d**) Furin cleavage site motif evolution across 10 US states. Four dominant motifs: NSPRRAR (wildtype), NSHRRAR (P681H), NSRRRAR (P681R), KSHRRAR (N679K+P681H). Percentages = proportion of the two most frequent motifs recorded per subsample, rounded to the nearest whole percent and expressed as a proportion of that two-motif pool. The pool covers 94.7–99.5% of sequences depending on phase, as stated in the figure. Minor motifs are not plotted, including NSHRRAR at 0.3% of Phase 3 and NSLRRAR at 0.1% of Phase 1.

#### 3.9.2. Furin Cleavage Site Position Dynamics (Figure 11b)

P681 rose from 2.0% ± 2.3% (Phase 1) to 57.5% ± 13.6% (Phase 2) and 99.6% ± 0.3% (Phase 3), reaching near-complete fixation from a near-absent baseline. This is consistent with Davies et al. [12], who linked Alpha’s P681H to increased transmissibility; Saito et al. [74], who showed Delta’s P681R enhanced syncytia formation; and Focosi et al. [131], who identified P681 among the residues mutated recurrently and independently across the Alpha, Beta, Gamma, and Delta variants of concern. Position 679 was mutated at appreciable frequency only in Phase 3, in 8.3% ± 9.7% of sequences, compared with 0.1% in each earlier phase, consistent with Omicron biology; the motif analysis confirms these are predominantly N679K. Mustafa et al. [128] demonstrated that N679K creates a novel cathepsin G cleavage site. Lubinski et al. [132], in a preprint, reported that N679K increases intrinsic furin cleavability beyond Alpha and Delta, despite lying outside the conventional furin binding pocket (P7 position). The remaining positions—S680 (0.0–0.1%), R682 (0.0%), R683 (0.0–0.1%), A684 (0.1%), R685 (0.0%)—remained invariant, consistent with Aynaud et al. [133], who found R685 essential and never mutated across 70,000 sequences. The near-complete invariance of the RRAR backbone indicates strong purifying selection on the polybasic core (Figure 11b).

#### 3.9.3. Furin Cleavage Site Mutation Heatmap (Figure 11c)

Mapping all 7 FCS positions across three phases revealed the most constrained parameter in the 13-parameter framework. Phase 1: P681 at 2.0%, all others <0.1%. Phase 2: P681 at 57.5%, all others <0.2%. Phase 3: P681 at 99.6%, N679 at 8.3%, all others ≤0.1%. Applying a common 5% threshold, 1 of 7 furin positions exceeds it in Phase 2 and 2 of 7 in Phase 3, against 7 of 17 ACE2 contact positions in Phase 3 (Parameter 7). This 1-position vs. 7-position contrast demonstrates fundamentally different evolutionary constraints. Wang et al. [134] provided the mechanistic explanation: O-glycosylation by GalNAc-T3/T7 suppresses furin cleavage of the wildtype sequence, but P681H/R mutations abrogate this glycosylation, enabling enhanced furin processing (Figure 11c).

#### 3.9.4. Furin Cleavage Site Motif Evolution (Figure 11d)

Phase 1: NSPRRAR (wildtype) ~98%, minor NSHRRAR (P681H) ~2%. Phase 2: NSHRRAR (P681H) rose to ~55%, overtaking wildtype (~45%), consistent with Alpha dominance. Lubinski et al. [72] showed P681H modestly increases furin cleavability, while Peacock et al. [73] noted P681H creates a less conventionally polybasic motif than P681R. Phase 3 exhibited a complex motif landscape dominated by NSRRRAR (P681R, ~92%), with KSHRRAR (N679K+P681H) at ~8%. The NSHRRAR motif (P681H without N679K) falls outside this two-motif pool and is recorded separately at 0.3% of all Phase 3 sequences. This is the Alpha motif, and its residual presence is consistent with Alpha-lineage sequences surviving into the July–December 2021 window; the later BA.4, BA.5, XBB, and BQ.1 lineages all descend from BA.2 and carry N679K, so they present as KSHRRAR rather than NSHRRAR. The P681R dominance in Phase 3 reflects Delta (B.1.617.2), which dominated the July–December 2021 window. The later BA.2.86/JN.1 lineages independently reverted to R681, but retain N679K and therefore carry a KSRRRAR motif that falls outside the four tracked here; they contribute negligibly to this sample, with the July 2022 onward window accounting for 2.4% of Phase 3 sequences (Section 3.15.1). The KSHRRAR motif (8%) reflects the Omicron lineages carrying the N679K+P681H combination, which includes BA.1 and BA.2 together with their BA.5 and XBB descendants. Mustafa et al. [128] showed this creates a novel cathepsin G cleavage site, while Lubinski et al. [132] demonstrated it increases intrinsic furin cleavability beyond both Alpha and Delta (Figure 11d).

In summary, this parameter provides: (1) Population-level furin cleavage site mutation burden (0.022 → 0.578 → 1.076) demonstrating a continuously ascending trajectory distinct from ACE2 contact mutations; (2) Near-complete P681H/R fixation (99.6%) documented across all ten states; (3) Absolute purifying selection on the RRAR polybasic backbone (R682, R683, R685 invariant) contrasted with adaptive evolution at flanking positions (P681, N679); (4) Population-level FCS motif tracking documenting NSPRRAR → NSHRRAR → NSRRRAR → KSHRRAR evolution across the three phases; (5) Cross-parameter comparison revealing 1-position convergence at the furin site versus 7-position diversification at the ACE2 interface.

### 3.10. N-Glycosylation Site Analysis (Parameter 9)

The SARS-CoV-2 spike protein carries 22 N-linked glycosylation sites per monomer, forming a dense glycan shield that constitutes approximately one-third of the spike’s total mass (Watanabe et al. [53]). This shield serves dual functions: structural stability during biosynthesis and immune evasion by masking antigenic epitopes from neutralizing antibodies. Huang et al. [135] showed that removing glycan shields elicited stronger and broader immune responses in animal models, providing direct experimental support for the glycan’s immune-evasion role. Among the variants of concern designated at the time of that analysis, the only N-glycosite change they report is at the moderately conserved N17 site [135]. This parameter tracks predicted N-glycosylation sequons (N-X-S/T, X ≠ P) across the spike protein for 10 US states. All counts reported in this section are of predicted sequons; glycan occupancy was not measured, and references to the glycan shield below describe the sequon complement that encodes it rather than the mature glycans themselves.

#### 3.10.1. N-Glycosylation Site Count Across Pandemic Phases (Figure 12a)

The mean N-glycosylation site count showed a distinctive trajectory: 21.967 ± 0.024 (Phase 1) → 22.019 ± 0.115 (Phase 2) → 20.927 ± 0.051 (Phase 3). Phase 1 was near-reference (22 sites) with minimal variation. Phase 2 showed a slight net gain of 0.019 sites, potentially from transient lineages introducing new sequons. Phase 3 showed a dramatic drop to 20.927—a net loss of 1.073 sites. This loss is consistent with the T19I mutation (Omicron) and T19R (Delta), which disrupt the N17 glycosylation sequon (N17-L18-T19). Wang et al. [83] confirmed via mass spectrometry and molecular dynamics that the N17 glycan is absent in both Delta and Omicron, resulting in enhanced surface accessibility of the NTD. The remarkably small Phase 3 SD (0.051) indicates this loss is uniform across all 10 US states, consistent with near-universal T19I/R fixation. Shajahan et al. [82] confirmed that glycosylation sites are highly conserved across VOCs except N17. This continuously ascending burden in furin cleavage (Parameter 8: 0.022 → 1.076) coinciding with glycan shield erosion (Parameter 9: 22.0 → 20.9) reveals simultaneous but distinct adaptive trajectories: the virus concurrently optimizes its S1/S2 cell entry machinery under transmissibility selection while experiencing localized glycan shield erosion at the NTD driven by immune pressure—two independent parallel adaptations converging to reshape the Spike surface (Figure 12a).

**Figure 12:**
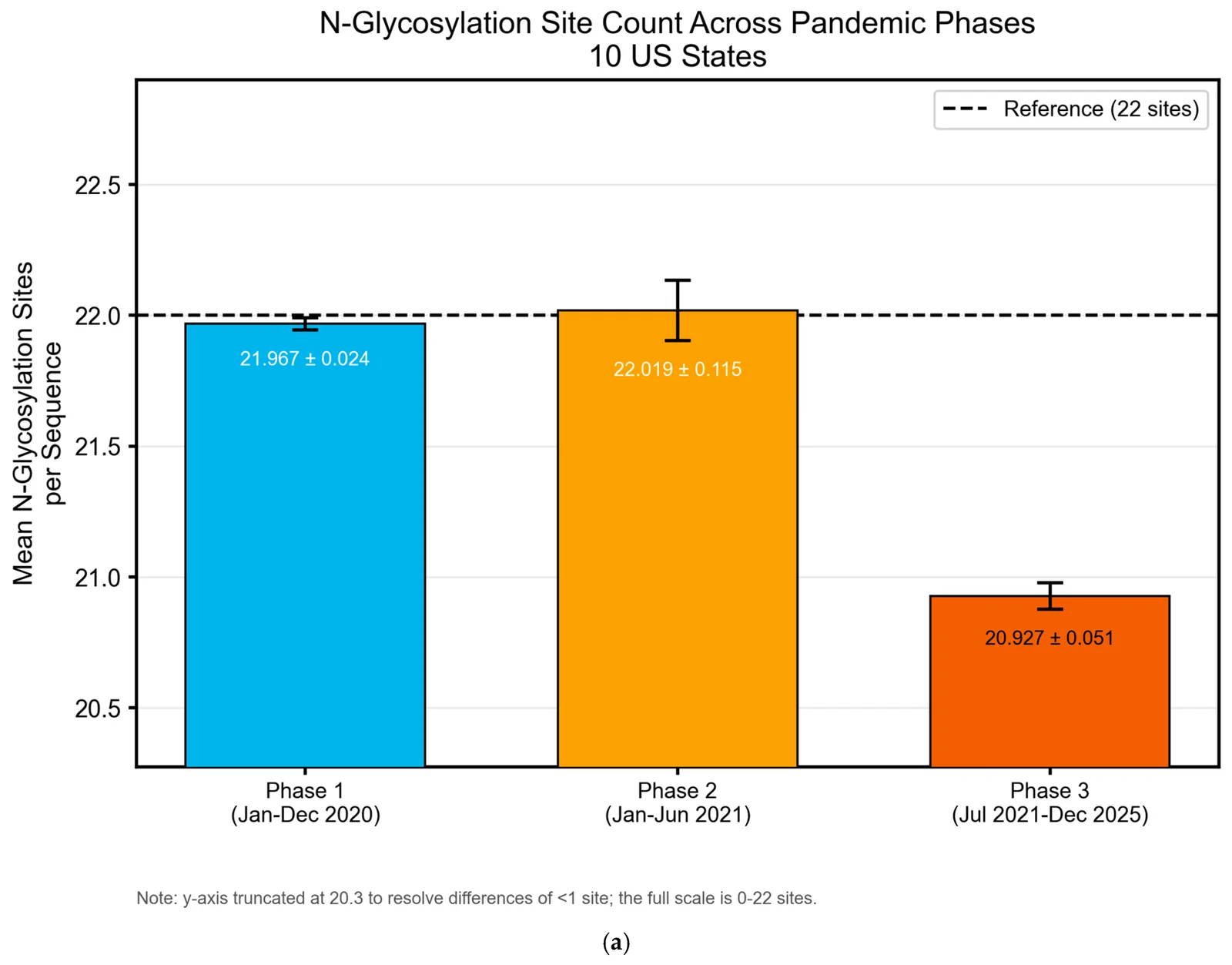

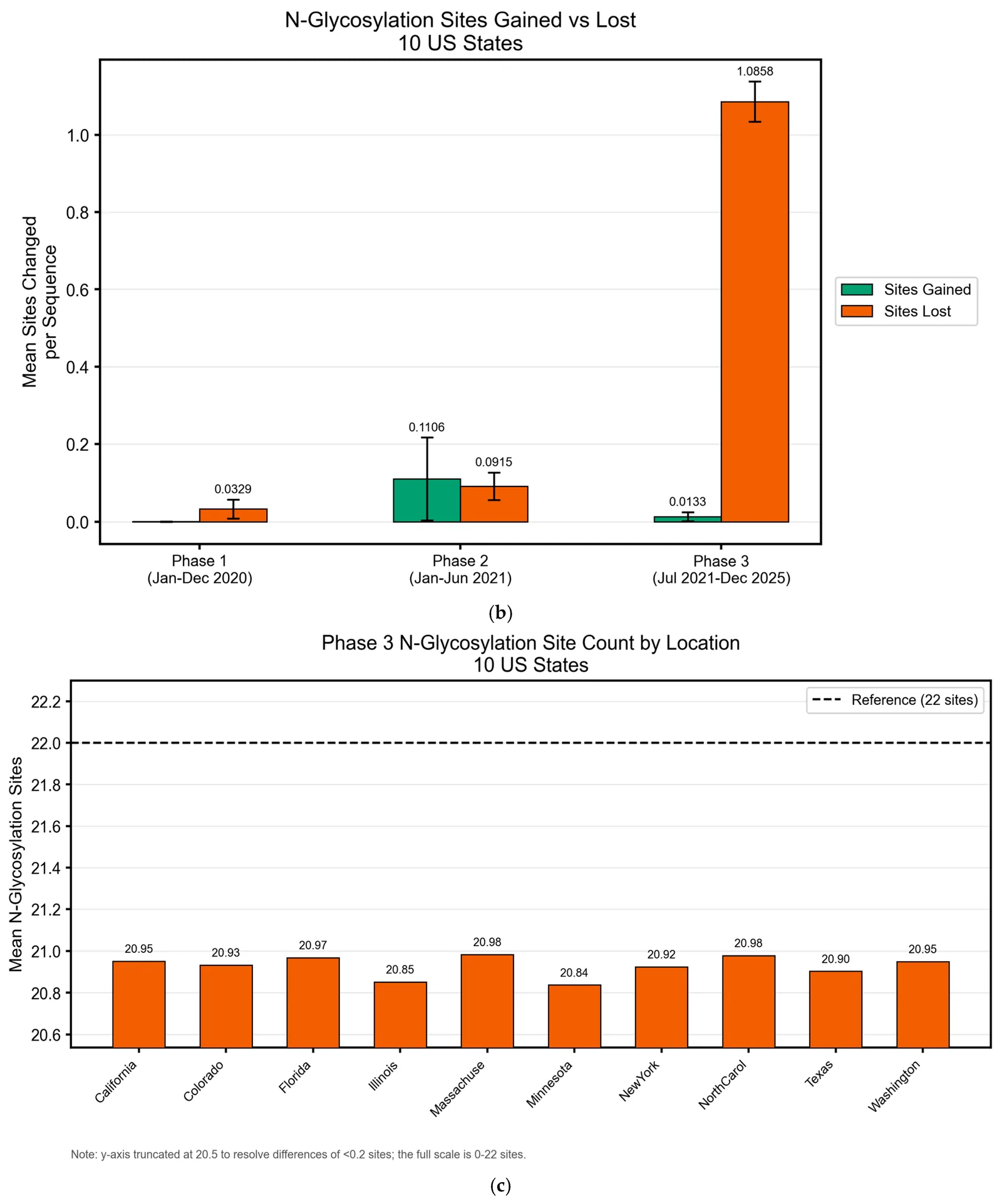
(**a**) Mean N-glycosylation site count across three pandemic phases for 10 US states. Bar height = cross-location mean. Dashed line = reference (22 sites, Wuhan-Hu-1). Error bars = ± SD. Individual location values omitted for clarity. (**b**) N-glycosylation sites gained versus lost across three pandemic phases for 10 US states. Green = sites gained. Red = sites lost. Error bars = ± SD. Individual location values omitted for clarity. (**c**) Phase 3 N-glycosylation site count by location for 10 US states. Each bar = location mean. Dashed line = reference (22 sites). All locations show consistent ~1 site loss.

#### 3.10.2. N-Glycosylation Sites Gained vs. Lost (Figure 12b)

Decomposing net change revealed striking asymmetry. Phase 1: gained = 0.0004 ± 0.0006, lost = 0.033 ± 0.024. Phase 2: gained = 0.111 ± 0.107, lost = 0.092 ± 0.035. Phase 3: gained = 0.013 ± 0.012, lost = 1.086 ± 0.052—an 81.9-fold imbalance. SARS-CoV-2 evolution overwhelmingly disrupts existing glycosylation sequons rather than creating new ones. Zhang et al. [136] showed that removal of individual glycosylation sites reduced pseudotype infectivity by almost 50-fold for N61D and by 5- to 10-fold for the RBD sites N331 and N343, with the magnitude of the effect predicted by reduced incorporation of spike into virions; this indicates a substantial fitness cost to losing even single sites. The loss of only ~1 site across a multi-year pandemic underscores extreme functional constraint, suggesting that severe structural and infectivity penalties—as demonstrated for critical sequons by Zhang et al. [136]—prevent more extensive glycan shield erosion without compensatory fitness-restoring mutations (Figure 12b).

#### 3.10.3. Phase 3 Geographic Distribution of Predicted Sequon Loss (Figure 12c)

Phase 3 counts ranged from 20.837 (Minnesota) to 20.983 (Massachusetts), mean 20.927 ± 0.051—the tightest cross-location convergence of any parameter analyzed. This uniformity indicates the loss of predicted sequons is driven by a single universally fixed mutation (T19I/R) rather than diverse location-specific mutations. Throughout this section, the measured quantity is the predicted N-X-S/T sequon count; glycan occupancy was not assayed, and the correspondence between sequon loss and loss of the mature glycan at N17 rests on the mass-spectrometry evidence of Wang et al. [83]. Liu et al. [137] showed that in BA.2.86 the K356T substitution creates an N-X-T sequon and an acquired N354 glycan, the first RBD glycan gain since the ancestral strain, but this had minimal population-level impact as the mean remains consistently below 21 (Figure 12c).

In summary, this parameter provides: (1) Population-level quantification of glycan shield erosion (22.0 → 22.0 → 20.9), documenting the loss of ~1 N-glycosylation site attributed to T19I/R disrupting N17; (2) Gain/loss asymmetry (81.9-fold imbalance) demonstrating SARS-CoV-2 overwhelmingly disrupts rather than creates glycosylation sequons; (3) Geographic uniformity (SD = 0.051)—the tightest convergence in the 13-parameter framework—confirming a single universally fixed mutation drives the change; (4) Cross-parameter integration: furin cleavage optimization (Parameter 8) coinciding with glycan shield erosion (Parameter 9) reveals parallel adaptive trajectories operating simultaneously across distinct functional domains.

### 3.11. Variant Concordance and Lineage Dynamics (Parameter 10)

To contextualize the molecular and structural shifts observed in Parameters 1 through 9, Parameter 10 validates the underlying epidemiological composition of the sequence dataset and reveals the surveillance-volume weighting that explains Phase 3 patterns observed throughout the manuscript. This parameter serves a dual purpose: (1) validating that the 5000-sequence subsamples faithfully capture the known epidemiological succession of variants of concern, and (2) quantifying lineage diversity dynamics across pandemic phases. If the subsamples failed to capture the Alpha → Delta → Omicron succession documented by global surveillance (Viana et al. [14]; Carabelli et al. [22]), all downstream parameters would be suspect. Shannon entropy tracking provides a novel quantitative measure of how population-level viral diversity evolved under competing pressures of immune selection, vaccination, and variant sweeps across 10 US states.

#### 3.11.1. Lineage Diversity Dynamics (Figure 13a)

Shannon entropy (raw) showed a V-shaped trajectory: 4.221 ± 0.433 (Phase 1) → 3.076 ± 0.621 (Phase 2) → 4.284 ± 0.379 (Phase 3). Unique lineage counts mirrored this: 93 ± 27 → 73 ± 17 → 95 ± 32. The Phase 1 high diversity reflects the initial proliferation of B.1.x lineages during 2020 before any VOC achieved dominance. The Phase 2 collapse (3.076) is consistent with Davies et al. [12], who showed Alpha’s 43–90% increased transmissibility enabled rapid displacement of pre-existing lineages. The Phase 3 recovery (4.284) reflects the explosive radiation of Delta into dozens of AY.* sub-lineages during the peak of global genomic surveillance in late 2021, layered alongside the initial emergence of Omicron sublineages. As documented in Section 3.11.2, Phase 3 sampling is overwhelmingly weighted toward late 2021 Delta dynamics due to GISAID submission volume concentration; the AY.* sub-lineage diversity (AY.103, AY.44, AY.3, and others) is the primary mathematical driver of the Phase 3 entropy recovery, with Carabelli et al. [22] confirming that Omicron’s unprecedented sublineage diversification contributed to the tail of the distribution. This V-shaped pattern has been qualitatively observed on surveillance dashboards (Nextstrain, CoVariants.org [84]) but has not been quantified with Shannon entropy using controlled subsampling and geographic replication (Figure 13a).

**Figure 13:**
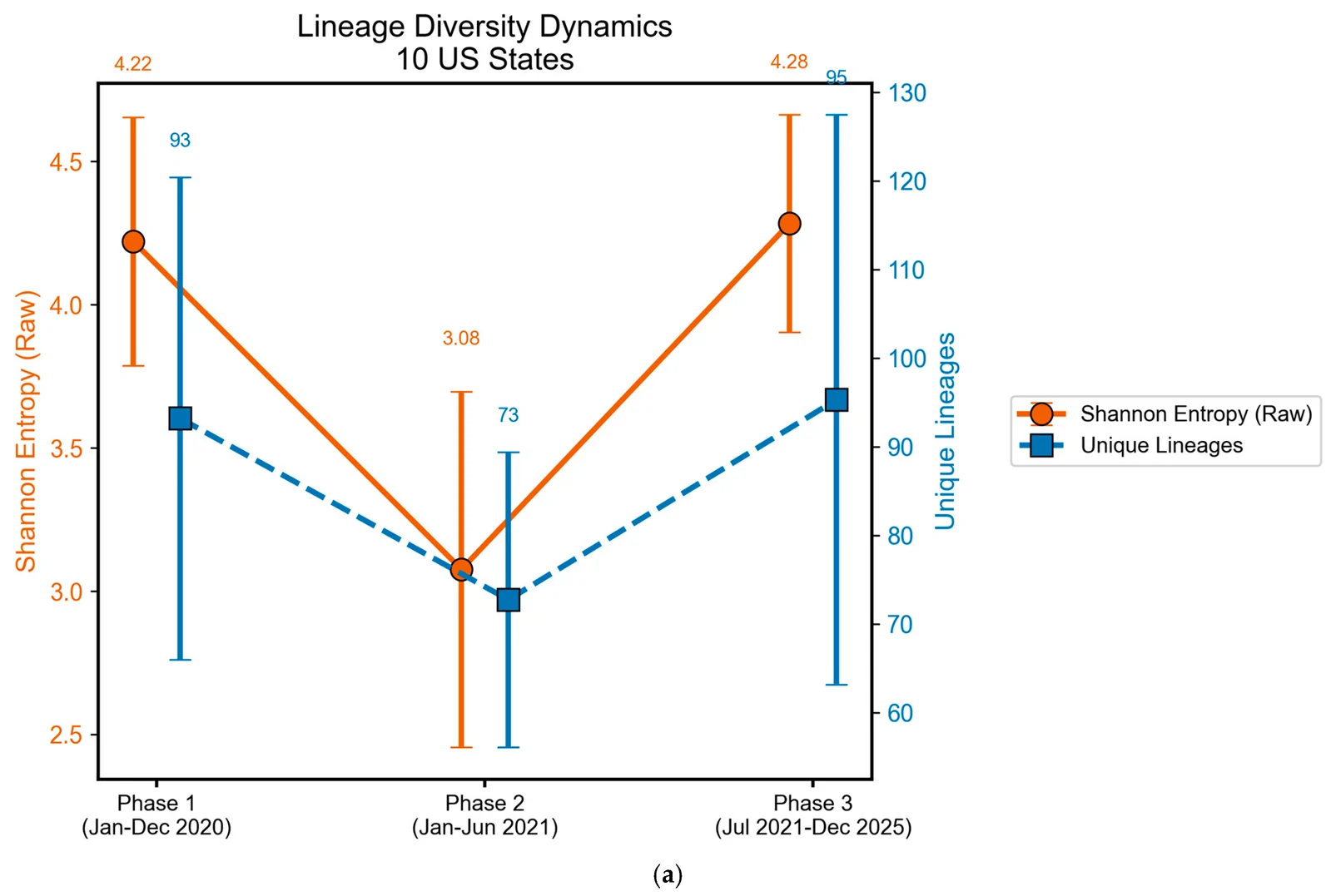

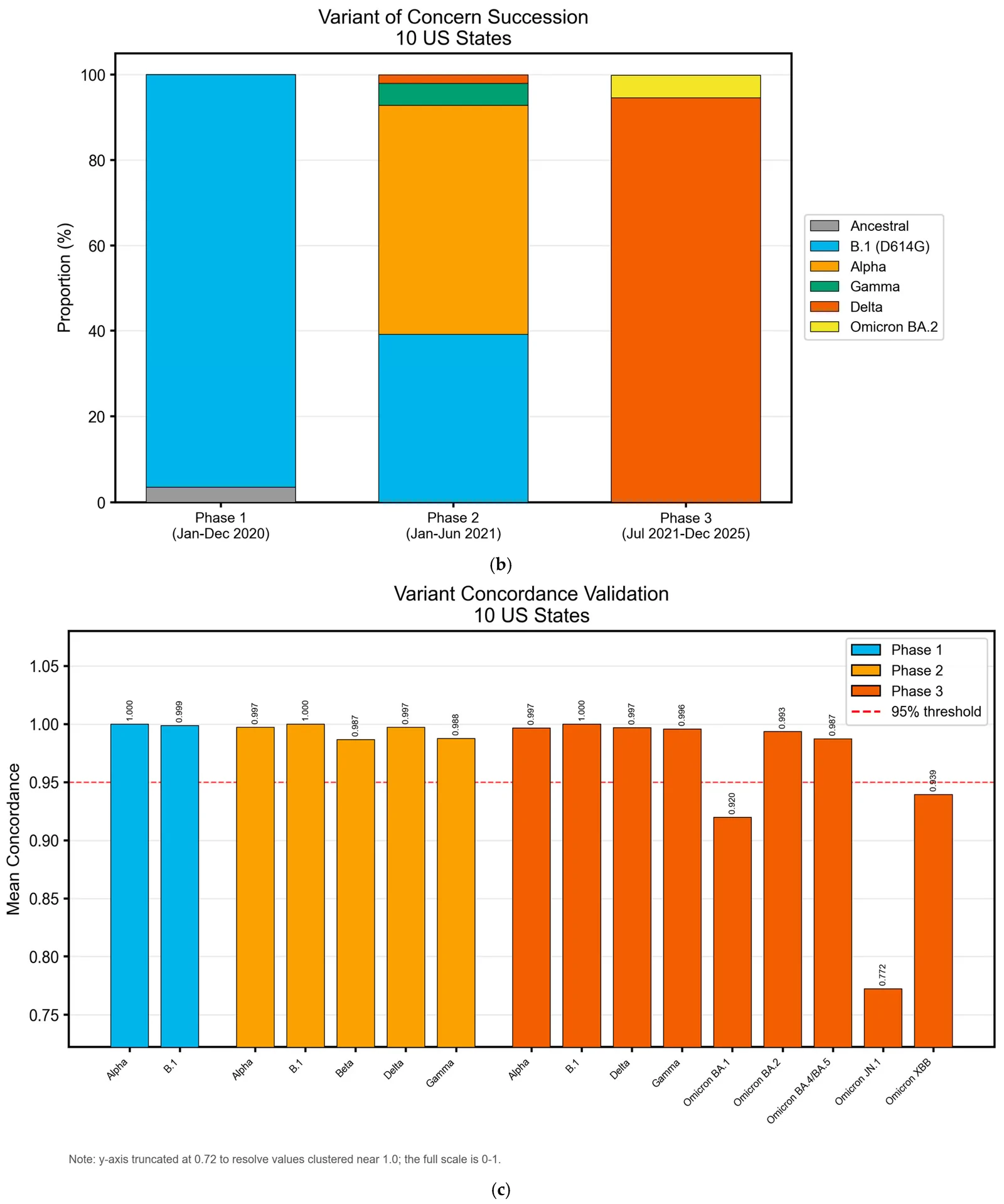
(**a**) Lineage diversity dynamics across three pandemic phases for 10 US states. Orange line (left y-axis) = Shannon entropy measuring lineage distribution evenness; higher values indicate more diverse populations. Blue dashed line (right y-axis) = count of unique Pango lineages. Error bars = ± SD across locations. Individual location values omitted for clarity. (**b**) Variant of Concern succession across three pandemic phases for 10 US states. Stacked bars show mean proportion per variant class. Ancestral = original Wuhan lineages (A, B without D614G). Delta includes B.1.617.2 and all AY.* sub-lineages. Omicron categories include BA.1, BA.2, BA.4/5, and Omicron Other (XBB, BQ, EG, JN). Individual location values omitted for clarity. (**c**) Variant concordance validation across three pandemic phases for 10 US states. Each bar = mean concordance (fraction of defining mutations present). Blue = Phase 1, orange = Phase 2, red = Phase 3. Red dashed line = 95% threshold. Individual location values omitted for clarity.

#### 3.11.2. Variant of Concern Succession (Figure 13b)

Variant succession confirmed the expected epidemiological progression. Phase 1 (January–December 2020): B.1 (D614G) dominated at 94.4%, with 4.8% ancestral lineages (A, B without D614G), consistent with Korber et al. [56], who demonstrated that D614G increased infectivity. Phase 2 (January–June 2021): Alpha rose to 47.6%, B.1 declined to 41.4%, with minor Gamma (5.3%) and emerging Delta (4.5%). Phase 3 (July 2021–December 2025): Delta (B.1.617.2 + AY.* sub-lineages) dominated at 90.4%, with Omicron BA.2 at 6.0%. This distribution explicitly reflects a GISAID surveillance skew: random subsampling draws proportionally from submission volumes, which peaked massively during the global Delta wave of late 2021 before sequencing efforts contracted significantly in 2022–2025. Consequently, Phase 3 macro-metrics throughout this manuscript are primarily driven by late-stage Delta dynamics and surveillance-weighted early Omicron, rather than the full Omicron evolutionary arc. This is a transparent, inherent feature of population-level surveillance-volume-weighted sampling, and validates the Delta dilution effects documented across Parameters 5–13. At the sub-lineage level, AY.103 (10.6%), AY.44 (8.3%), and AY.3 (5.8%) were the most prevalent Delta sub-lineages, providing finer-grained resolution than standard VOC reporting. Obermeyer et al. [138] analyzed 6.4 million genomes and identified fitness-associated mutations; our controlled subsampling confirms their population-level findings within a replicated framework (Figure 13b).

#### 3.11.3. Variant Concordance Validation (Figure 13c)

Concordance validation confirmed that sequences carry the expected defining mutations. B.1 (D614G): 0.999 across all phases, confirming near-universal D614G presence. Alpha: 1.000 (Phase 1) → 0.997 (Phase 2) → 0.997 (Phase 3). Delta: 0.997 in both Phase 2 and Phase 3. Beta (0.987) and Gamma (0.988–0.996) likewise exceeded the 0.95 threshold. Three late Omicron lineages fell below the threshold: XBB (0.939), BA.1 (0.920), and JN.1 (0.772). These are the lineages represented by the fewest sequences in the volume-weighted Phase 3 sample—409, 198, and 96 sequences, respectively, against 45,177 for Delta—so their concordance estimates rest on a median of 9 to 16 sequences per triplicate and are correspondingly unstable. JN.1 is additionally the most recently emerged of these lineages and the most divergent from its BA.2.86 parent, having evolved rapidly under heavy immune pressure [28], so the set of defining substitutions used for scoring is also the least settled. Harvey et al. [43] cataloged defining mutations for each VOC; our concordance scores validate that subsampled sequences faithfully carry these signatures. This concordance approach checks whether randomly subsampled sequences carry variant-defining mutations, and is used here as a quality control metric (Figure 13c).

In summary, this parameter provides: (1) V-shaped Shannon entropy trajectory (4.22 → 3.08 → 4.28) quantifying diversity collapse during Alpha dominance and recovery during Omicron diversification, with controlled subsampling and geographic replication; (2) Complete variant succession accounting (B.1 → Alpha → Delta → Omicron) within controlled 5000-sequence subsamples, validating framework representativeness; (3) Concordance validation as a novel quality control metric confirming >98% concordance for all variants represented by more than 500 sequences; (4) Delta sub-lineage resolution (AY.103, AY.44, AY.3) providing finer dynamics than standard VOC reporting.

### 3.12. Amino Acid Property Changes (Parameter 11)

Not all amino acid substitutions are equal—a mutation that conserves charge, size, and hydrophobicity (conservative substitution) is less likely to alter protein function than one that radically changes these properties. This parameter quantifies what proportion of SARS-CoV-2 mutations are conservative versus radical across four physicochemical properties: charge, hydrophobicity, size, and polarity. Declining conservation rates indicate later variants carry increasingly radical mutations that alter surface properties and potentially disrupt antibody binding. Bloom and Neher (2023) [139] showed immune-selected mutations in the spike RBD tend to change amino acid charge and hydrophobicity more than expected by chance, consistent with antibody escape requiring surface property changes. This parameter extends that insight genome-wide across all proteins for 10 US states.

#### 3.12.1. Amino Acid Property Conservation Across Phases (Figure 14a)

Conservation declined across the physicochemical property schemes examined; note that hydrophobicity and polarity are not independent measures under our binary classification and track identically, so three distinct trajectories are represented. Charge conservation: 66.7% ± 1.5% (Phase 1) → 62.5% ± 1.7% (Phase 2) → 60.9% ± 1.7% (Phase 3). Hydrophobicity and polarity conservation (which tracked identically based on our binary property classification matrix, as these properties are inversely linked in the amino acid physicochemical classification schema): 56.8% ± 1.7% → 47.3% ± 2.5% → 44.6% ± 1.8%. Size conservation: 50.6% ± 2.7% → 44.8% ± 1.4% → 47.5% ± 0.8%. Charge conservation declined most gradually (5.8 percentage point drop), while hydrophobicity showed the steepest decline (12.2 points), consistent with Starr et al. [124], who showed that ACE2 binding mutations preferentially alter hydrophobic contacts. Size conservation showed a non-monotonic pattern (50.6% → 44.8% → 47.5%), suggesting Omicron mutations are more size-conservative than Alpha/Delta mutations.

**Figure 14:**
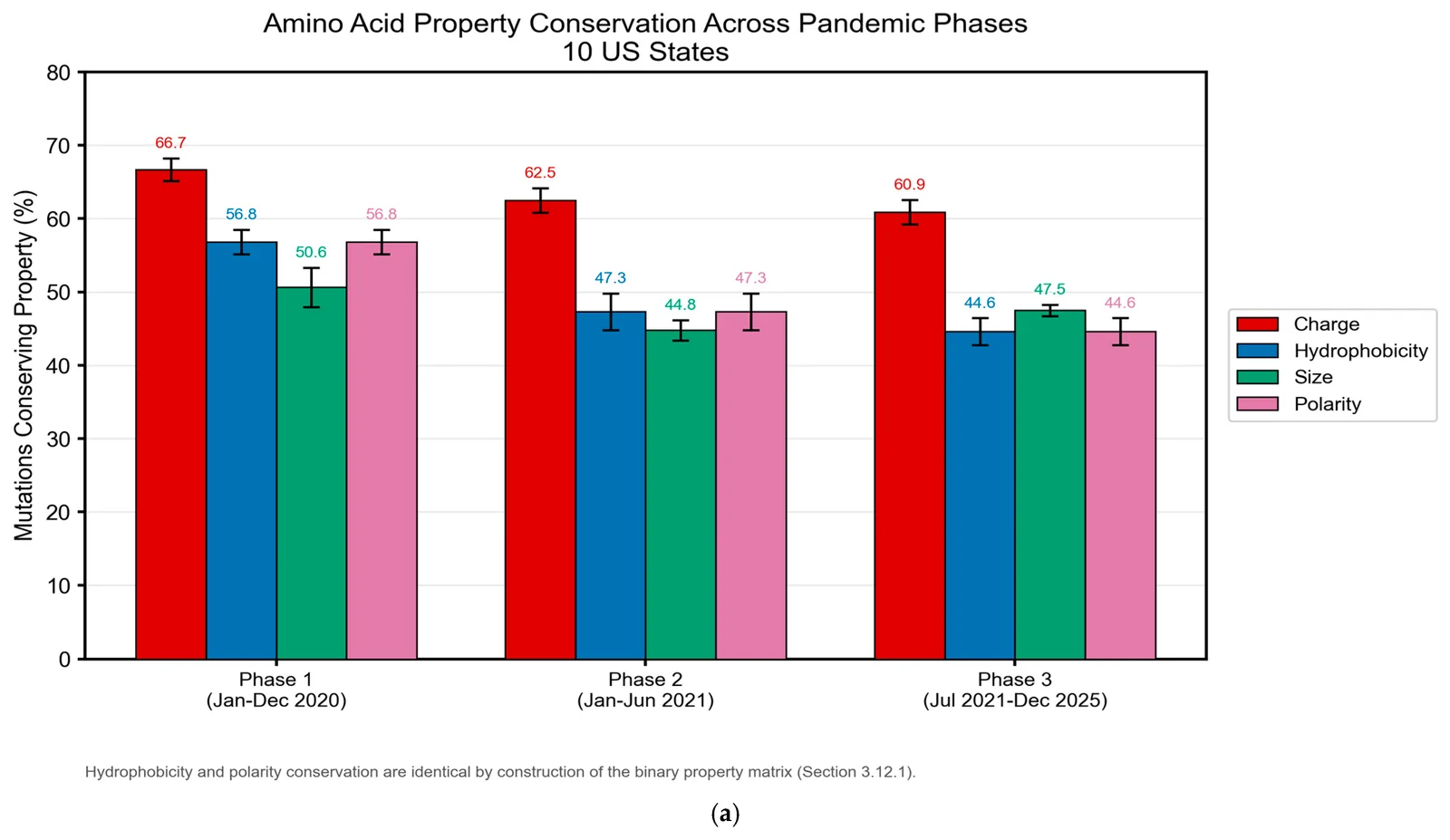

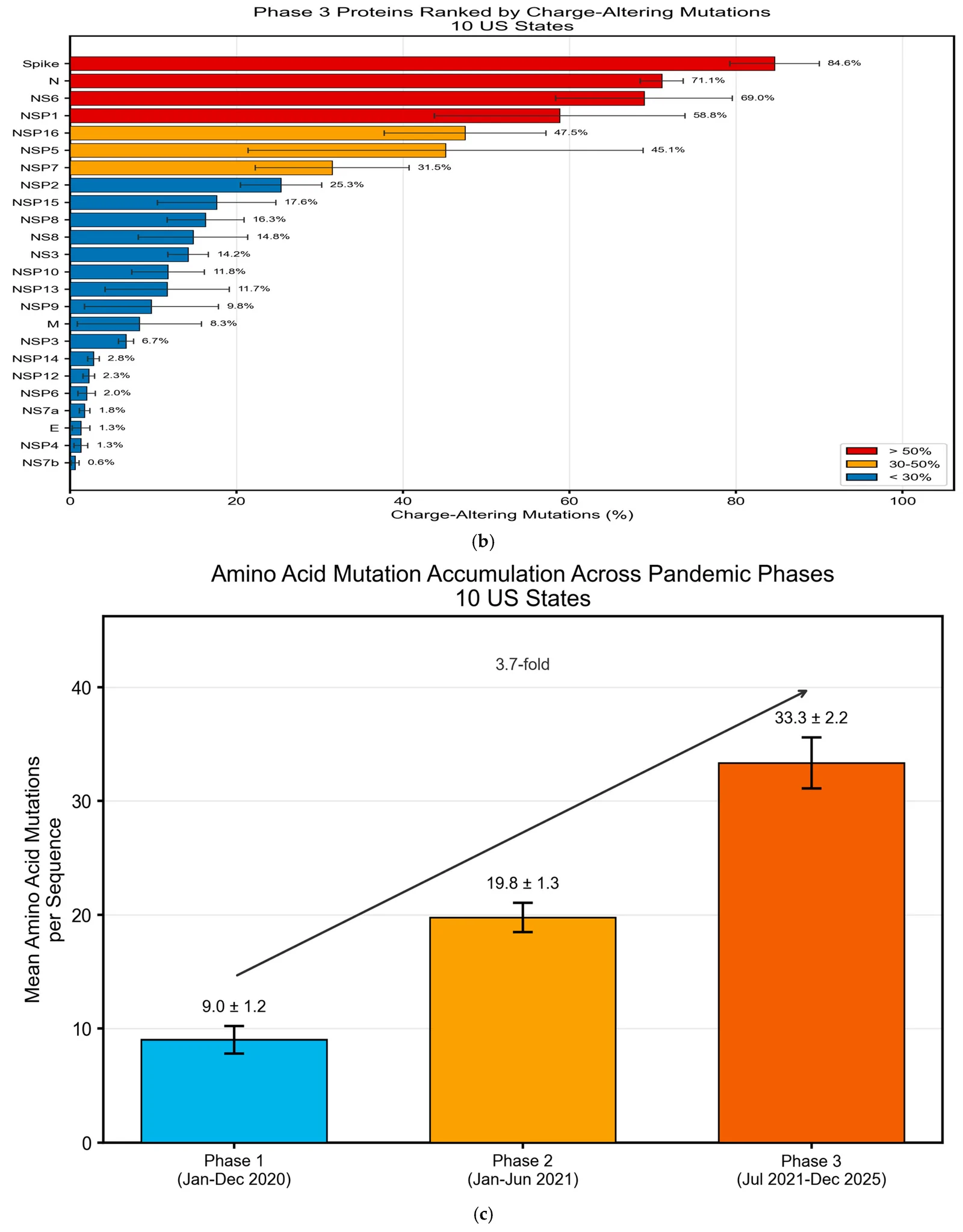
(**a**) Amino acid property conservation rates across three pandemic phases for 10 US states. Grouped bars show percentage of mutations conserving charge (red), hydrophobicity (blue), size (green), and polarity (pink). Error bars = ± SD across locations. Individual location values omitted for clarity. (**b**) Phase 3 proteins ranked by percentage of charge-altering mutations for 10 US states. Red bars >50%, orange bars 30–50%, blue bars <30%. Error bars = ± SD across locations. Individual location values omitted for clarity. (**c**) Mean amino acid mutations per sequence across three pandemic phases for 10 US states. Error bars = ± SD across locations. Individual location values omitted for clarity.

#### 3.12.2. Protein-Level Charge-Altering Mutations (Figure 14b)

Phase 3 protein ranking by charge-altering mutations revealed Spike (84.6%) and N protein (71.1%) as the proteins most tolerant of charge-altering mutations. This is biologically expected: Spike is under the strongest antibody-mediated immune selection, requiring surface charge alterations to escape neutralization (Xue et al. [122]; Wang et al. [126]). N protein is the most abundant viral protein and a major T cell response target (Grifoni et al. [140]). NS6 (69.0%) and NSP1 (58.8%) also showed high charge change rates. NSP1 suppresses host innate immunity by blocking the ribosomal mRNA channel (Thoms et al. [141]); charge-altering mutations may modulate this interaction. The ranking Spike > N > NS6 > NSP1 indicates which proteins are under strongest selection for surface property alteration (Figure 14b).

#### 3.12.3. Amino Acid Mutation Accumulation (Figure 14c)

Mean amino acid mutations per sequence increased progressively: 9.0 ± 1.2 (Phase 1) → 19.8 ± 1.3 (Phase 2) → 33.3 ± 2.2 (Phase 3), a 3.7-fold increase. Note that these values differ from the Parameter 13 totals by up to 17% (9.12 → 23.90 → 36.03) because Parameter 11 counts only amino acid substitutions successfully mapped to physicochemical property categories, excluding stop codons, ambiguous positions, and indels that are captured in the broader Parameter 13 count. While early pandemic accumulation closely tracked canonical molecular clock estimates (Duchene et al. [23]), the deceleration of net fixation in Phase 3—despite its 54-month duration—suggests the virus is approaching functional fitness plateaus where purifying selection prunes further accumulation. This progressive accumulation contrasts with the V-shaped lineage diversity in Parameter 10, confirming that while total mutation count increases steadily, the functional impact and lineage composition of each phase are shaped by distinct selection regimes (Figure 14c).

In summary, this parameter provides: (1) Population-level tracking of amino acid property conservation rates (charge, hydrophobicity, size, polarity) across pandemic phases, revealing progressive decline consistent with immune-driven radical substitution selection; (2) Protein-level ranking of charge-altering mutations identifying Spike (84.6%) and N (71.1%) as the most charge-disruptive, correlating with known antibody and T cell selection pressure; (3) Non-monotonic size conservation (50.6% → 44.8% → 47.5%), suggesting Omicron mutations are more size-conservative than Delta; (4) Cross-parameter integration: 3.7-fold mutation accumulation (9.0 → 33.3) coincides with declining property conservation, quantifying the shift from conservative to radical substitutions.

### 3.13. Protein-Wise dN/dS Ranking (Parameter 12)

The ratio of nonsynonymous to synonymous substitution rates (dN/dS) is the canonical metric for detecting the direction and intensity of natural selection acting on protein-coding sequences. When dN/dS > 1, nonsynonymous mutations accumulate faster than expected under neutral evolution, signaling positive (diversifying) selection favoring amino acid change. When dN/dS < 1, purifying selection dominates, eliminating deleterious nonsynonymous mutations. Parameter 12 extends the genome-wide dN/dS analysis established in Parameter 3 by decomposing selection pressure at the individual protein level, ranking all 25 individually analyzable SARS-CoV-2 proteins by mean dN/dS across 10 US states for each pandemic phase; the genome-wide value from Parameter 3 is reported alongside these rankings for reference and is excluded from all protein counts. This protein-level resolution identifies which proteins are under the strongest positive selection pressure—key information for understanding immune escape, vaccine target stability, and viral fitness trade-offs.

Published genome-wide dN/dS analyses (Gupta et al. [18]; Gayvert et al. [19]) report aggregate values but do not systematically rank all proteins across phases. The protein-level ranking approach used here, applied across up to 5000 subsampled sequences per state per phase (n = 137,059 total sequences), provides a comprehensive selection landscape that complements per-site FUBAR analysis (Parameter 4).

#### 3.13.1. Phase 1 Protein Selection Landscape (Pre-VOC) (Figure 15a)

During Phase 1, the pre-VOC period characterized by ancestral SARS-CoV-2 lineages, the selection landscape was dominated by strong positive selection on accessory and structural proteins. ORF3a ranked highest (dN/dS = 4.86 ± 1.51), followed by ORF8 (3.43 ± 1.93) and NSP16 (2.17 ± 1.92). Notably, the Spike protein showed near-neutral selection with a slight diversifying trend (dN/dS = 1.14 ± 0.33), consistent with early-pandemic diversification before immune pressure drove directional selection. NSP12 (RNA-dependent RNA polymerase) was essentially neutral (1.01 ± 0.26). At the purifying end, M (0.11 ± 0.05), NSP15 (0.13 ± 0.06), and NSP3 (0.14 ± 0.03) were under the strongest purifying selection, consistent with their essential replication and structural functions. The mean genome-wide dN/dS was 0.61 ± 0.06 (moderate purifying selection) (Figure 15a).

#### 3.13.2. Phase 2 Protein Selection Landscape (Alpha/Delta) (Figure 15b)

Phase 2 marked a dramatic reshuffling of the protein selection hierarchy. ORF8 emerged as the most positively selected protein (dN/dS = 14.23 ± 10.30), an extraordinary value consistent with the intense mutational pressure immediately preceding the widespread ORF8 truncations documented in later Alpha/Delta sublineages, capturing the diversification phase before stop-codon fixation. ORF7b rose from moderate purifying (0.69) to strong positive selection (4.61 ± 4.14). The Spike protein showed the largest directional shift among structural proteins, rising from 1.14 to 4.09 ± 1.15, reflecting intense antibody-mediated selection pressure from vaccine rollout and prior infection immunity. NSP13 became positively selected (1.17 ± 0.39), a notable finding as the NSP13 helicase is a replication complex component not typically considered a major selection target. In contrast, NSP5 (main protease) dropped sharply from positive (1.25) to strong purifying selection (0.35 ± 0.17), suggesting functional constraint reasserted after initial diversification. The exceptionally high standard deviation observed for ORF8 in Phase 2 (14.23 ± 10.30) reflects intense, state-specific episodic selection spikes rather than a uniform national trend, likely driven by localized variant founder effects in which individual states experienced dominant Alpha or Delta sublineages carrying distinct ORF8 truncation-preceding mutations (Figure 15b).

#### 3.13.3. Phase 3 Protein Selection Landscape (Delta to Early Omicron) (Figure 15c)

Phase 3, spanning the Delta wave and early Omicron sublineages, produced the most broad-based positive selection landscape observed across all three phases. A total of 11 of 25 proteins showed dN/dS > 1, compared to 9 in Phase 1 and 8 in Phase 2, and the genome-wide dN/dS rose to 1.10 ± 0.07—the only phase to exceed the neutral threshold at the genome level. ORF7b led the ranking (8.64 ± 13.57; the exceptionally high SD reflects intense, state-specific episodic positive selection rather than a uniform national trend, consistent with localized Omicron subvariant founder effects), followed by Spike (3.47 ± 0.73) and ORF7a (3.22 ± 0.75). The simultaneous elevation of ORF7a and ORF7b under positive selection in Phase 3—neither showed this in Phase 1—suggests coordinated immune evasion through accessory protein diversification. The M protein, under strong purifying selection in Phases 1 and 2 (0.11 and 0.26), became positively selected in Phase 3 (1.80 ± 0.41), documented here at population scale across US states, suggesting Omicron M protein mutations contribute to immune escape or host interaction changes. NSP14 (proofreading exonuclease) also became positively selected (1.40 ± 0.16), consistent with evidence that specific NSP14 mutations accelerate genomic diversity by reducing ExoN proofreading fidelity (Takada et al. [142]) (Figure 15c).

**Figure 15:**
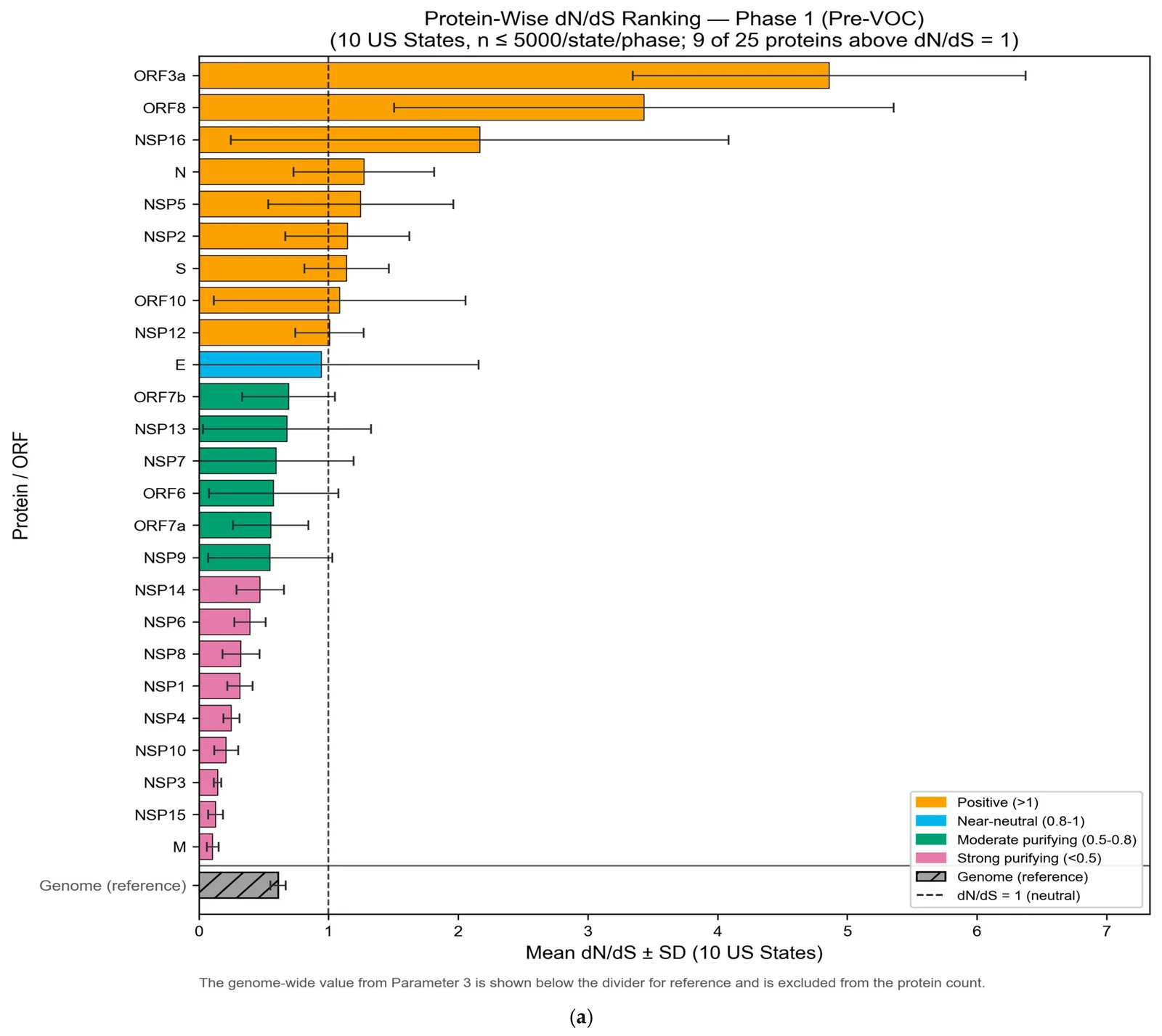

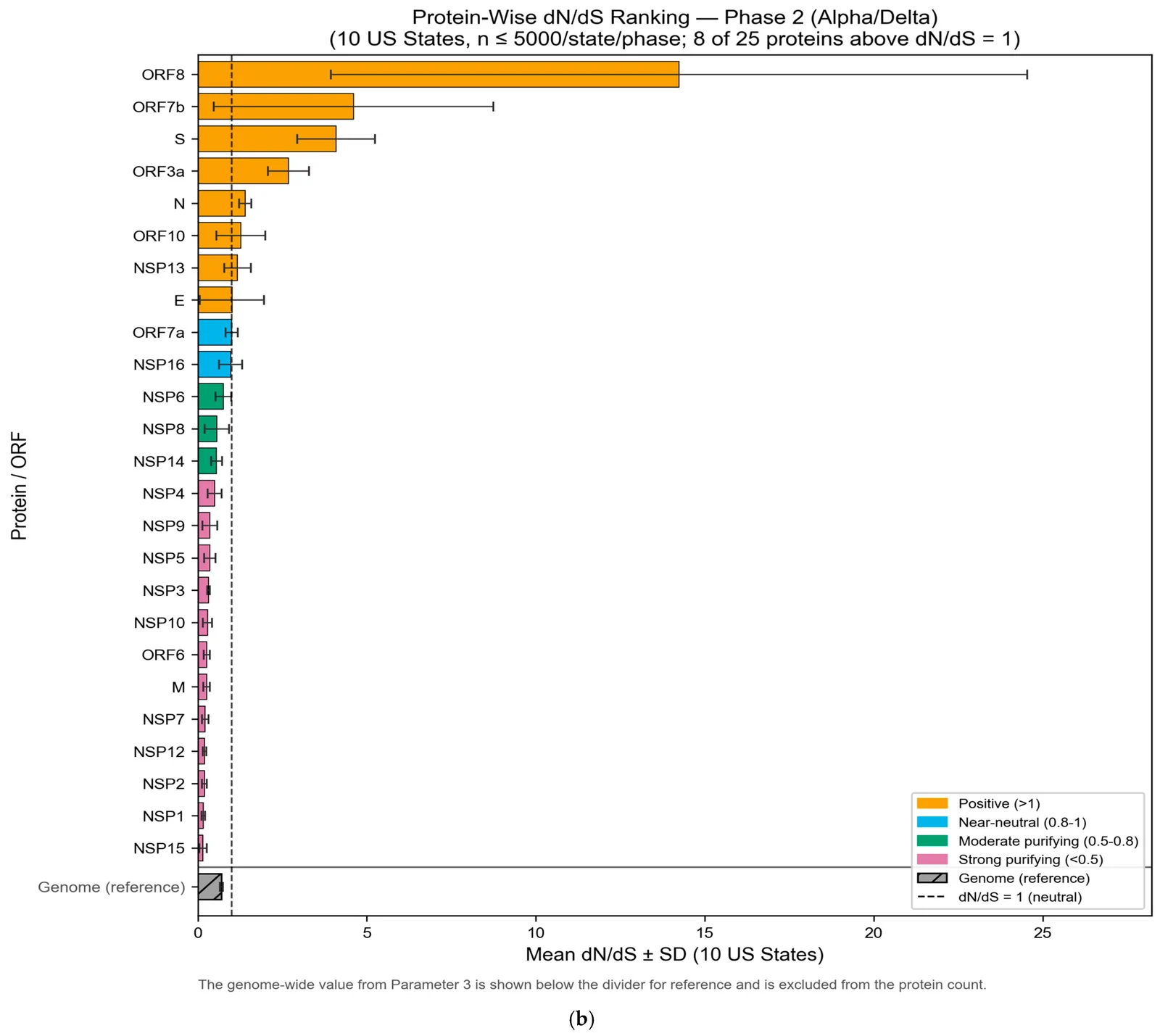

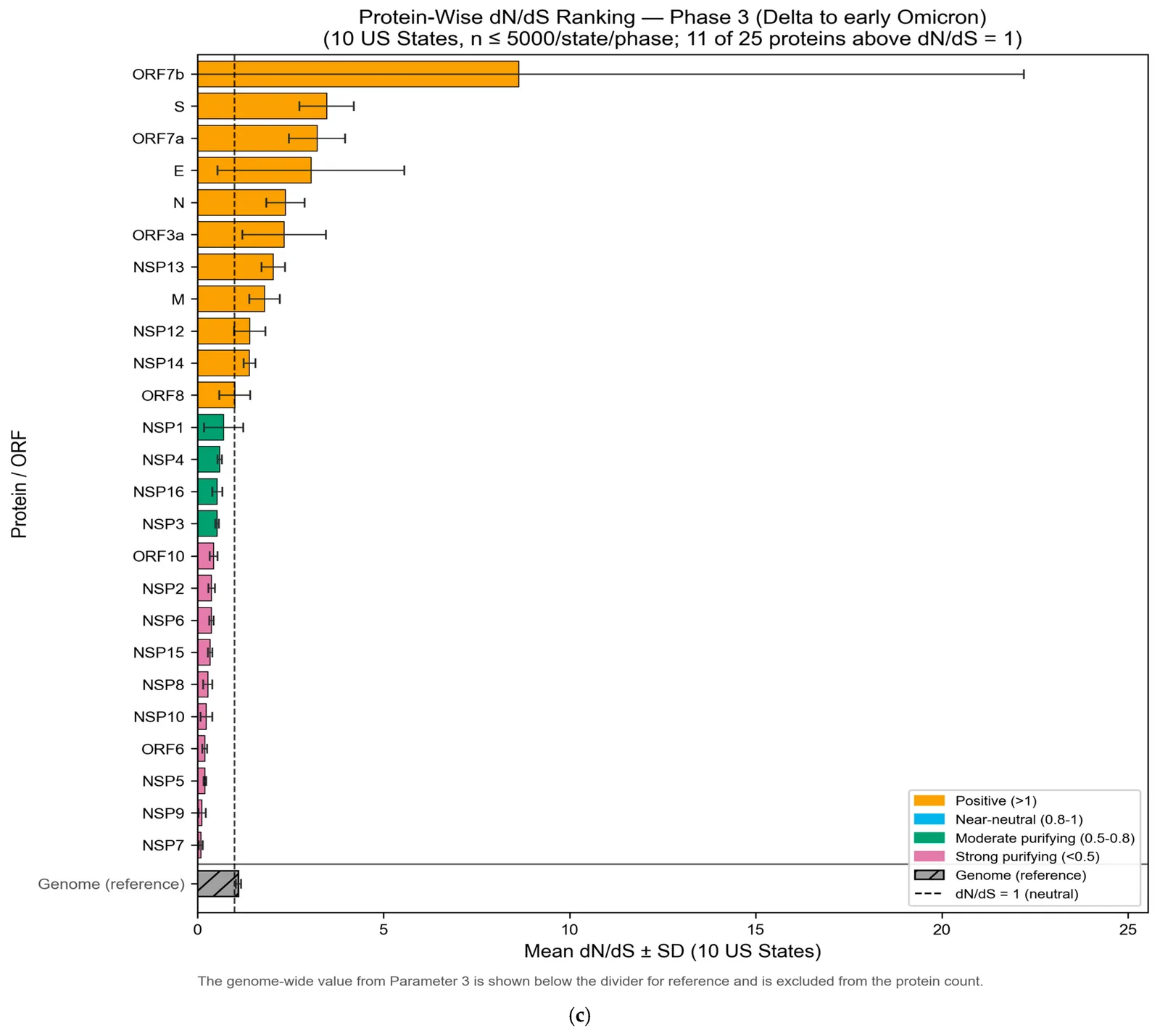

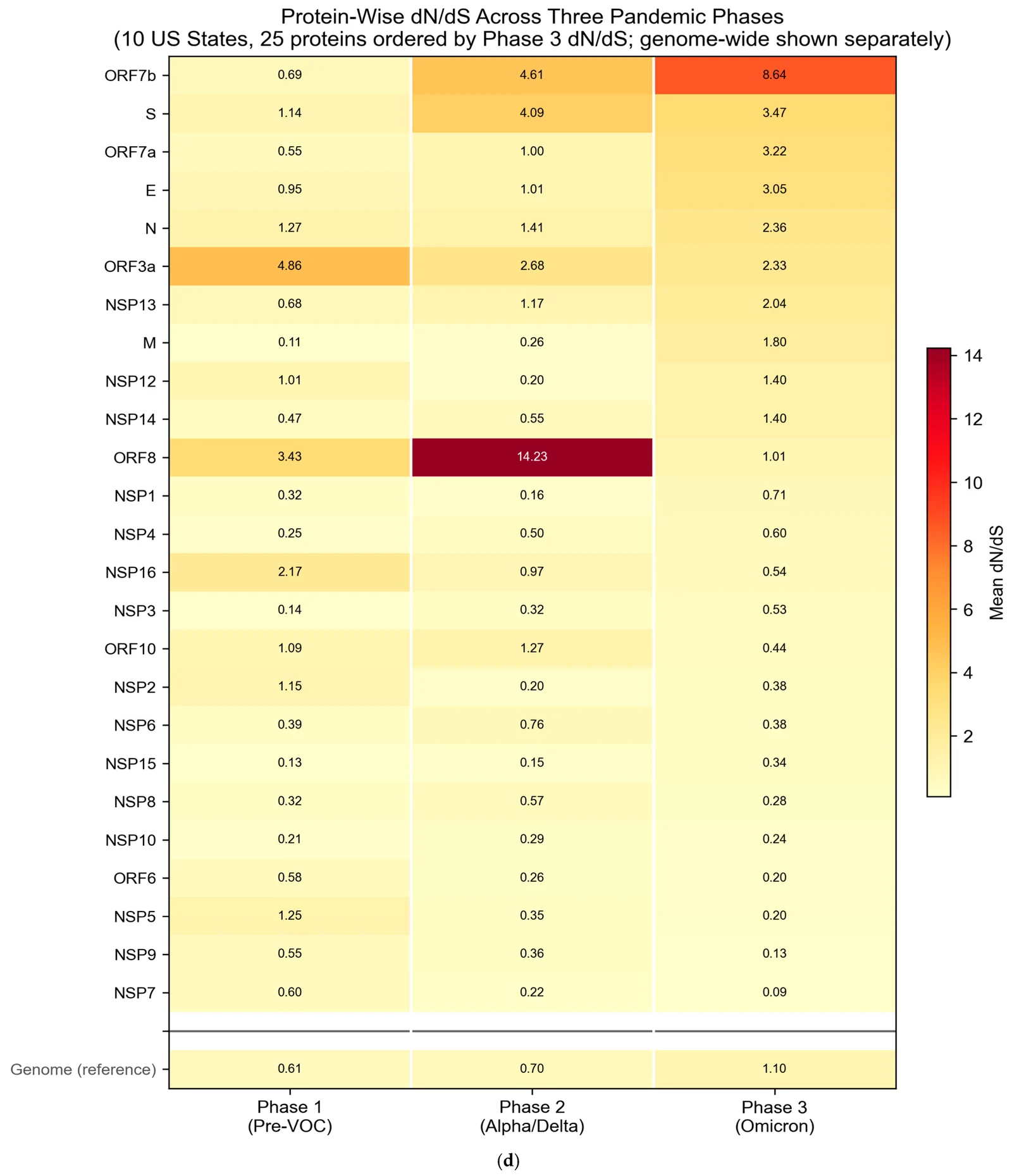
(**a**) Phase 1 protein-wise dN/dS ranking for 10 US States (Pre-VOC period). Horizontal bars show mean dN/dS ± SD across 10 states. Colors indicate selection category: orange = positive (>1), blue = near-neutral (0.8–1.0), green = moderate purifying (0.5–0.8), pink = strong purifying (<0.5). The divider row labeled “Parameter 3” in the plot is the genome-wide dN/dS of Section 3.2; the analysis scripts number that parameter differently, as noted in Section 2.9. Dashed vertical line at dN/dS = 1.0 (neutral threshold). Error bars = SD across states. (**b**) Phase 2 protein-wise dN/dS ranking for 10 US states (Alpha/Delta period). Format as Figure 15a. Note the extreme dN/dS for ORF8 (14.23 ± 10.30) and the shift of Spike into strong positive selection territory (4.09 ± 1.15). Error bars = SD across states. (**c**) Phase 3 protein-wise dN/dS ranking for 10 US states (Delta to early Omicron period). Eleven proteins show dN/dS > 1 (positive selection), the highest count across any phase; the genome-wide dN/dS of Section 3.2 is plotted separately, on the divider row, the plot labels “Parameter 3”, and is not counted among them. Note that genome-wide dN/dS = 1.10, the only phase to exceed the neutral threshold. Error bars = SD across states. (**d**) Cross-phase heatmap of protein-wise dN/dS for 10 US states. Rows ordered by Phase 3 dN/dS (descending). Cell values = mean dN/dS. Color scale: yellow (low) to red (high). White gridlines separate phases.

#### 3.13.4. Cross-Phase Protein Selection Dynamics (Figure 15d)

The cross-phase heatmap reveals four distinct evolutionary trajectories across all 25 proteins. Trajectory 1—Consistently positive selection: ORF3a, ORF8, N, and S showed dN/dS > 1 across all three phases, identifying them as under continuous diversifying selection pressure throughout the pandemic. Trajectory 2—Progressive elevation: Spike (1.14 → 4.09 → 3.47), ORF7a (0.55 → 1.00 → 3.22), and NSP13 (0.68 → 1.17 → 2.04) showed monotonic increases in positive selection, consistent with escalating immune pressure. Trajectory 3—Phase 2 peak then relaxation: ORF8 (3.43 → 14.23 → 1.01) showed a non-monotonic pattern, suggesting functional re-optimization. NSP5 (1.25 → 0.35 → 0.20) declined monotonically from positive selection in Phase 1 to strong constraint thereafter. Trajectory 4—Late-phase emergence: M (0.11 → 0.26 → 1.80) and NSP14 (0.47 → 0.55 → 1.40) transitioned to positive selection only in Phase 3, while NSP12 (1.01 → 0.20 → 1.40) followed a V-shaped course—positive in Phase 1, constrained in Phase 2, positive again in Phase 3—identifying selection targets not apparent from earlier phases (Figure 15d).

In summary, this parameter provides: (1) Comprehensive protein-level dN/dS ranking across all 25 individually analyzable SARS-CoV-2 proteins for three pandemic phases, giving a systematic ranking across phases for US states; (2) NSP14 transition to positive selection in Phase 3 (1.40 ± 0.16) is compatible with an alteration in proofreading fidelity and motivates direct experimental testing; elevated dN/dS alone does not establish a change in fidelity; (3) Four evolutionary trajectories (consistently positive, progressively elevated, Phase 2 peak, late-phase emergence) provide a framework for understanding which proteins drive each pandemic phase’s adaptive evolution.

### 3.14. Protein Domain-Specific Mutation Load (Parameter 13)

While Parameter 2 (genome-wide dN/dS) and Parameter 12 (protein-wise dN/dS ranking) characterize the selective forces acting on SARS-CoV-2 proteins, Parameter 13 quantifies the absolute and normalized mutation burden accumulated per protein across pandemic phases. Mutation load—measured as mean amino acid mutations per sequence and normalized per 100 amino acids to account for protein size—provides a complementary lens: it captures not only which proteins are under selection pressure but how much mutational divergence from the ancestral sequence has actually accumulated in the circulating population. This distinction is critical because a protein under strong positive selection may still carry fewer total mutations than a large structural protein, while a small accessory protein may carry disproportionate mutation density relative to its size.

Parameter 13 is applied across the annotated SARS-CoV-2 proteins represented in the GISAID substitution metadata, using up to 5000 subsampled sequences per state per phase (10 states x 3 phases, n = 137,059 total sequences; sampling seed 42, triplicate split seed 123). Both absolute mutation load (mean mutations per sequence) and normalized load (mutations per 100 amino acids) are computed. Triplicate coefficient of variation was 0.84% (mean) and 3.93% (maximum), confirming high reproducibility across all location-phase combinations.

#### 3.14.1. Overall Mutation Load Across Pandemic Phases (Figure 16a,b)

Mean amino acid mutations per sequence increased substantially across all three phases. The grand mean across 10 US states was 9.12 ± 1.14 (Phase 1), 23.90 ± 2.16 (Phase 2), and 36.03 ± 2.75 (Phase 3). This represents a 2.62-fold increase from Phase 1 to Phase 2 and a 1.51-fold increase from Phase 2 to Phase 3, yielding a 3.95-fold overall increase from Phase 1 to Phase 3. The decelerating fold-change between phases (2.62× vs. 1.51×) is consistent with Markov et al. [143], who described a sustained but non-accelerating rate of amino acid substitution accumulation as SARS-CoV-2 adapted to the human host. All 24 proteins were mutated in every location–phase combination, confirming that mutation load is a genome-wide phenomenon rather than isolated to specific regions. The protein count differs between parameters because the annotation sources differ: Parameters 11 and 13 use the GISAID substitution annotation and its NS naming for accessory proteins, whereas Parameter 12 uses the de novo codon reconstruction across the 25 individually analyzable coding regions defined in Section 2.6.

Location-level variation in Phase 3 ranged from 32.76 mutations/sequence (Florida) to 42.86 (New York), a 1.31× within-phase spread. This geographic heterogeneity is modest compared to the 3.95× cross-phase increase, indicating that temporal phase is the dominant driver of mutation load variation. The elevated New York Phase 3 burden follows from the same compositional gradient described in Section 3.2: New York carries the largest early-Omicron fraction of any state (31.6 percent), and Omicron content correlates with mutation load at r = +0.97 across the ten states. The identical mechanism accounts for New York’s highest divergence and its lowest genome-wide dN/dS, since the additional Omicron substitutions are disproportionately synonymous (Figure 16a,b).

#### 3.14.2. Protein-Level Normalized Mutation Load (Figure 16c)

When normalized per 100 amino acids, NS7b (43 AA) and NS7a (121 AA) emerged as the most disproportionately mutated proteins in Phase 3, with normalized loads of 1.906 and 1.665 mutations per 100 AA, representing 40.81-fold and 31.47-fold increases from Phase 1. García-García et al. [144] demonstrated that ORF7a and ORF7b (referred to as NS7a and NS7b in this framework) expression inhibits the IFN-I response and increases proinflammatory cytokines, creating conditions favorable for viral survival. Critically, Guo et al. [145] reported that an ORF7a-deleted Delta isolate exhibited a 2.1-fold higher IFNβ IC50 than the intact ORF7a isolate, that is, was more resistant rather than more sensitive. The authors describe this result as unexpected and report that a second Delta isolate carrying an intact ORF7a showed similar or even higher interferon sensitivity, which they take to suggest that ORF7a may not be a dominant mechanism contributing to interferon antagonism in live virus. The functional role of ORF7a in interferon antagonism therefore remains unsettled, and no causal claim is made here. Liang (2023) [146] further documented that Omicron BA.4 specifically showed elevated ORF7b amino acid mutation percentages (2.182–2.667%) relative to other VOCs at the variant level. The 40.81-fold and 31.47-fold normalized load increases we document in NS7b and NS7a across ten geographically distinct US state cohorts provide the population-scale counterpart to these molecular findings, quantifying how extensively these proteins diversified as the immune landscape matured. Spike ranked fourth by normalized load in Phase 3 (0.983 per 100 AA), behind NS7b, NS7a, and N protein—despite carrying the highest absolute mutation burden (12.51 mutations/sequence). NSP7 (83 AA) showed the lowest fold change across phases (0.27×), representing a net reduction in accumulated mutations and confirming it as the most evolutionarily conserved protein, consistent with the essential role of NSP7 interface residues in the replication complex (Biswal et al. [147]) (Figure 16c).

**Figure 16:**
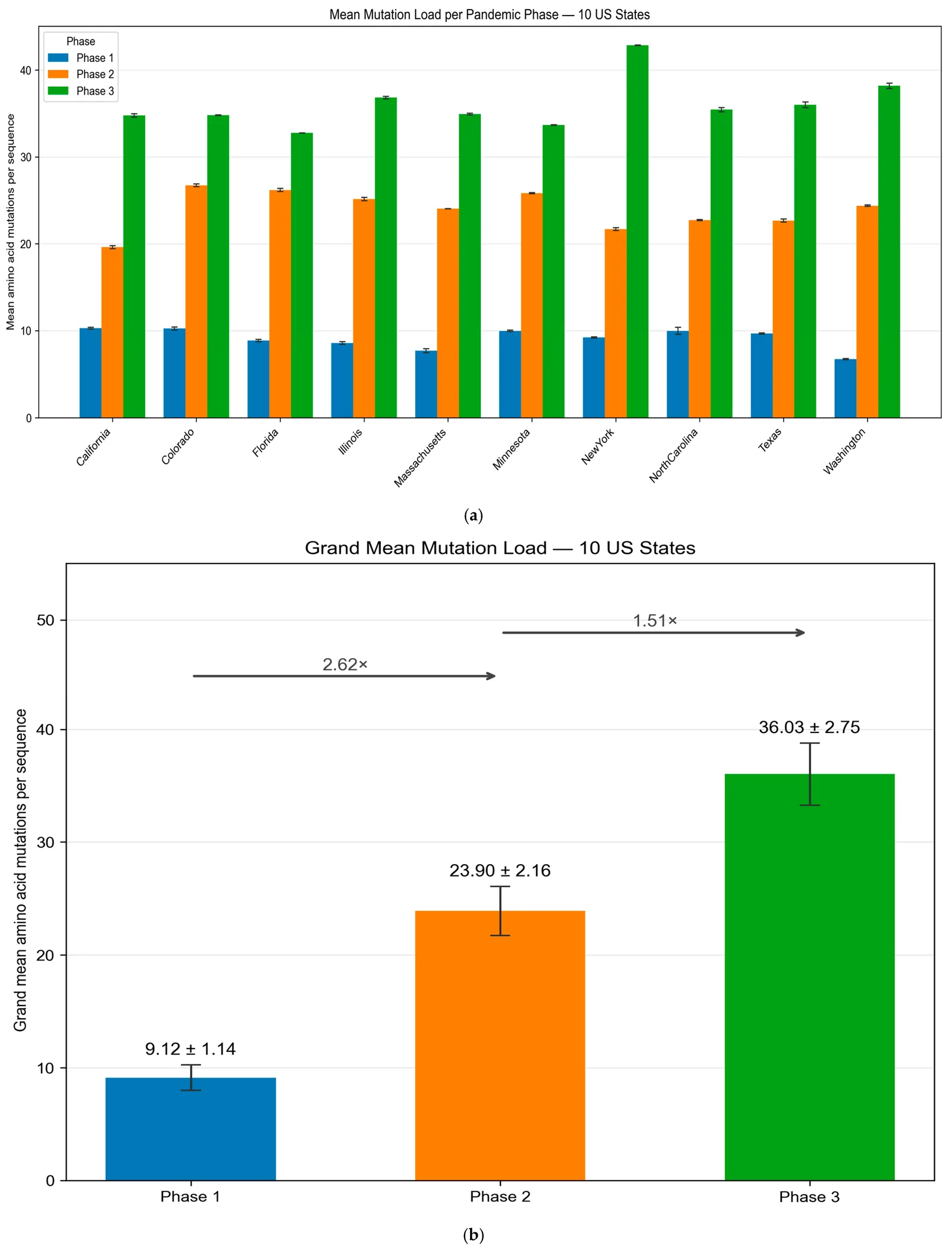

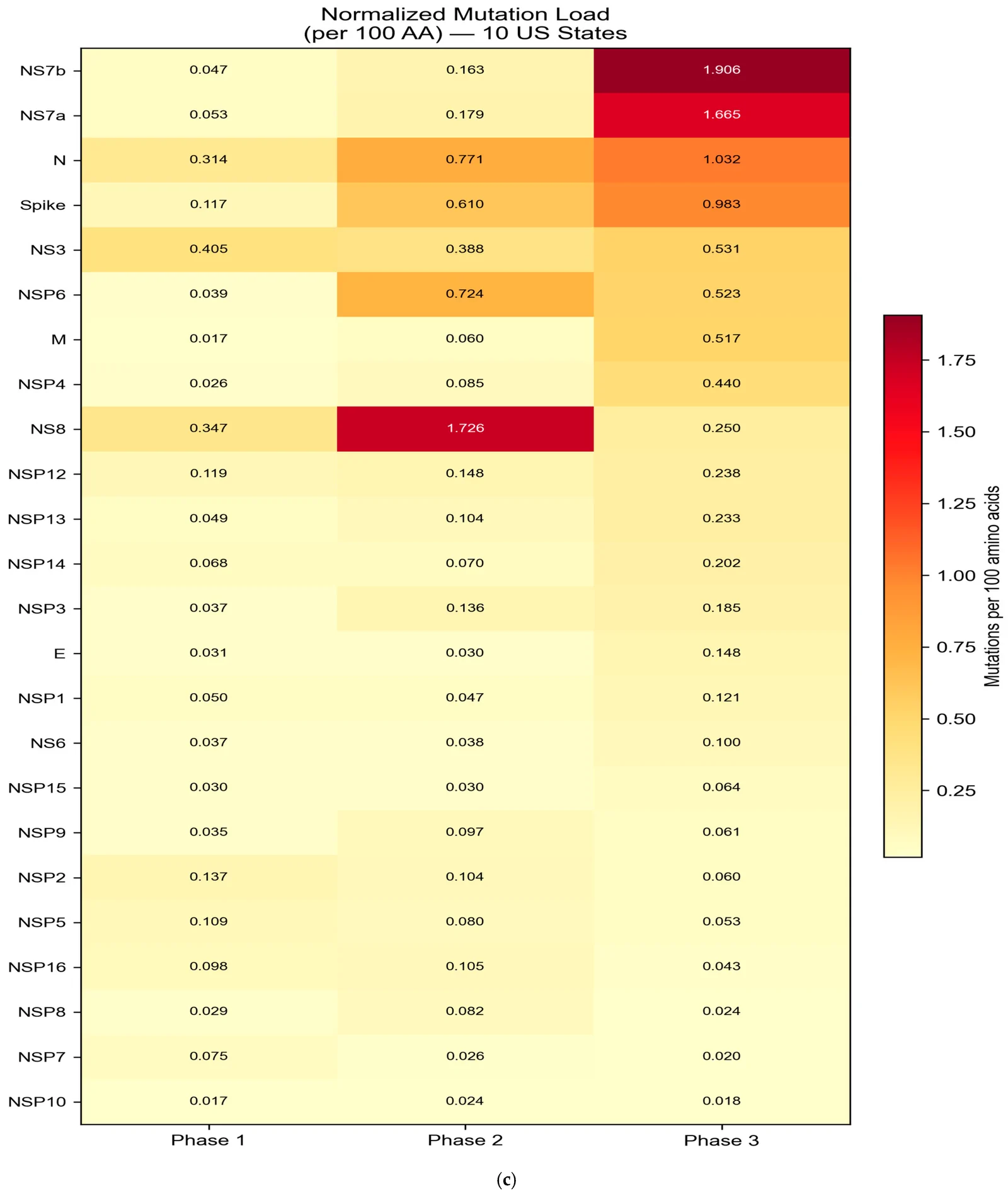

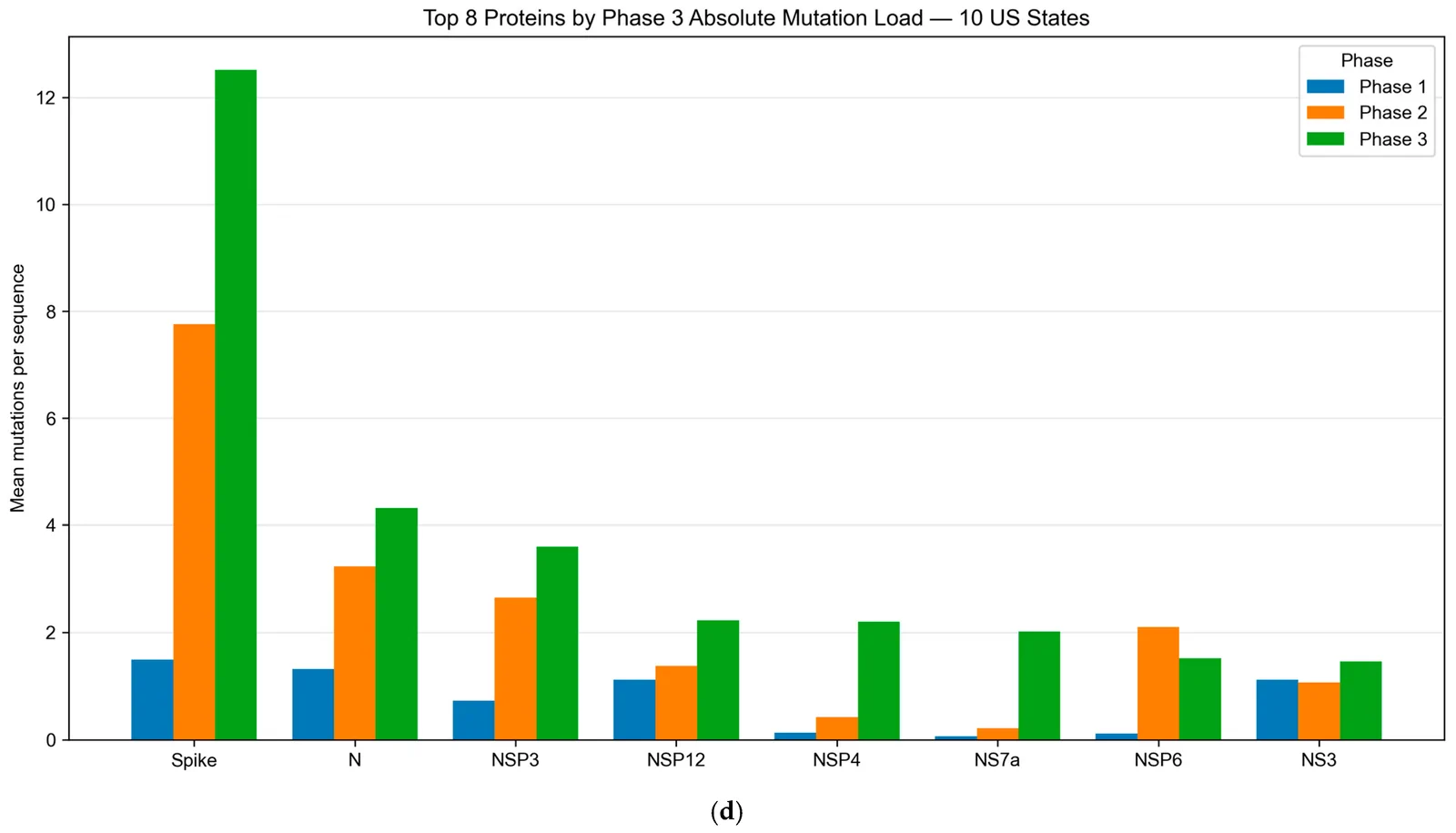
(**a**) Mean amino acid mutations per sequence per pandemic phase for 10 US states. Grouped bars show per-state means; colors indicate phase (blue = Phase 1, orange = Phase 2, green = Phase 3). Error bars = ± SD across triplicates within each state. (**b**) Grand mean mutation load across 10 US states per pandemic phase. Values above bars = grand mean ± SD. Arrows show fold-change between phases (2.62× Phase 1→2, 1.51× Phase 2→3). (**c**) Protein-level normalized mutation load heatmap (mutations per 100 AA) across pandemic phases for 10 US states. Rows ordered by Phase 3 normalized load (descending). Cell values = mean normalized load. Color scale: yellow (low) to red (high). (**d**) Top 8 proteins by Phase 3 absolute mutation load for 10 US states. Grouped bars show mean mutations per sequence per phase (blue = Phase 1, orange = Phase 2, green = Phase 3).

#### 3.14.3. Top Proteins by Phase 3 Absolute Mutation Load (Figure 16d)

While normalized metrics highlight the extreme evolutionary pressures acting on small accessory proteins, absolute mutation counts remain dominated by large structural targets. By absolute mutations per sequence, Spike led all proteins in every phase: 1.49 (Phase 1), 7.76 (Phase 2), and 12.51 (Phase 3) mutations/sequence. These counts exceed the Spike mutation burden reported in Parameter 5 (1.47 → 6.17 → 9.46) because the two parameters derive substitutions differently: Parameter 5 counts amino acid differences recovered from de novo pairwise alignment of translated Spike coding sequences, subject to the parameter-level exclusion of ambiguous positions described in Section 2.5, whereas Parameter 13 counts every substitution annotated in the GISAID “AA Substitutions” metadata. The two agree closely in Phase 1 (1.47 versus 1.49) and diverge as sequence divergence and ambiguous-position masking increase. N protein ranked second in absolute load by Phase 3, consistent with its role as both a replication scaffold and major T cell target (Grifoni et al. [140]). NSP3 (papain-like protease, the largest SARS-CoV-2 protein at 1945 residues) ranked third, accumulating the most mutations of any non-structural protein, followed by NSP12 (RdRp, the RNA-dependent RNA polymerase) in fourth. NSP16, which ranked third by Phase 1 dN/dS in Parameter 12, showed a normalized load that was essentially flat between Phases 1 and 2 before falling sharply in Phase 3 (0.098 → 0.105 → 0.043 per 100 AA). This pattern is mechanistically explained by NSP16’s function as a 2′-O-methyltransferase required for viral RNA capping and innate immune evasion: coronavirus mutants with disrupted NSP16 activity are consistently attenuated in vitro and in vivo due to recognition of unmethylated viral RNA by host sensors IFIT1 and MDA5 (Schindewolf and Menachery [148]). The high Phase 1 dN/dS reflects early diversification to optimize 2′-O-methyltransferase immune evasion efficiency; once a functionally optimized sequence became fixed, purifying selection reasserted—a “diversify-then-fix” evolutionary pattern for NSP16 at the population level (Figure 16d).

In summary, this parameter provides: (1) NS7b and NS7a are the most disproportionately mutated proteins genome-wide in Phase 3 (40.81× and 31.47× normalized load increases), connecting population-level mutation density to known interferon antagonism functions (García-García et al. [144]; Guo et al. [145]; Liang 2023 [146]); (2) NSP7 is the most evolutionarily conserved protein across all phases (0.27× fold change, declining normalized load), identifying it as a primary candidate for a stable, cross-variant antiviral drug target; (3) Spike leads absolute mutation load in all phases (12.51 mutations/sequence in Phase 3) but ranks fourth by normalized load—the distinction between absolute and normalized burden reveals that small accessory proteins carry greater per-residue mutation pressure than Spike; (4) NSP16 cross-parameter paradox: high Phase 1 dN/dS (Parameter 12, rank 3) with declining Phase 3 normalized load (0.098 → 0.043 per 100 AA) documents a “diversify then fix” evolutionary pattern mechanistically linked to 2′-O-methyltransferase immune evasion optimization (Schindewolf and Menachery [148]) for this protein at population level.

Evolutionary Predictions and Vaccine Implications

The integrated framework supports several data-grounded predictions about the continued evolution of SARS-CoV-2. First, the reproducible, immune-driven convergence observed at receptor-binding-domain antibody-escape sites (notably 484, 452, 478, and 142) across ten geographically distinct state populations suggests that future evolution will continue to concentrate at these and adjacent epitope residues, with convergent escape variants arising repeatedly and independently under sustained population immunity. Second, the distinctive ORF8 trajectory, in which early diversifying selection gives way to progressive loss of function, points to ongoing streamlining of dispensable accessory genes as the virus optimizes for endemic transmission. Third, the continued remodeling of furin-cleavage and glycosylation sites indicates that entry efficiency and immune shielding will remain active targets of selection. For vaccine strategy, these findings carry two implications. The genomic regions identified as under strong purifying selection, including the functionally constrained replication core and the Paxlovid target NSP5 (Mpro), represent comparatively stable targets whose conservation makes them attractive for broadly protective vaccines and antivirals. Conversely, the predictable, convergent nature of immune escape at defined spike positions suggests that vaccine-strain updates could anticipate likely escape sites rather than react to them, and that multivalent or conserved-epitope approaches may offer more durable population-level protection than strain-matched designs alone. These projections are offered as hypotheses supported by the present multi-parameter analysis and warrant prospective validation.

### 3.15. Sensitivity Analyses of the Phase 3 Findings

Phase 3 spans four and a half years and is weighted by submission volume toward its earliest months, so two sensitivity analyses are reported here to test how far the Phase 3 results depend on that composition. Both re-aggregate the existing subsampled cohort and required no additional sequence retrieval, and neither alters any result reported above.

#### 3.15.1. Temporal Subdivision of Phase 3

Phase 3 spans fifty-four months and, because sequences are drawn in proportion to submission volume, is dominated by the high-volume Delta period (Section 2.2.2). To test whether the reported trends persist when the later Omicron signal is no longer diluted, the Phase 3 sample was partitioned by collection date (Table 3) into three windows: 3a (July–December 2021), 3b (January–June 2022), and 3c (July 2022 onward). All 50,000 Phase 3 sequences carried complete collection dates and were assigned; the partition is exhaustive.

The reported trends persist and are substantially stronger once the Delta-dominated window is separated. Spike mutation burden rises 3.6-fold from 3a to 3c, and burden at the seventeen-position ACE2 panel rises from 0.044 to 7.20. The magnitude of the latter reflects panel composition rather than a discontinuity in evolutionary rate: the Delta-defining receptor-binding domain substitutions L452R and T478K both fall outside the seventeen structurally defined panel positions, whereas the Omicron lineages carry substitutions at K417, S477, E484, Q493, Q498, N501, and Y505, all of which are within it. The phase-averaged Phase 3 value of 0.614 therefore understates interface remodeling during the Omicron period, and the apparent decline from Phase 2 (0.739) is a composition effect: Phase 2 was Alpha-dominated, and Alpha carries N501Y within the panel, while the Delta genomes that dominate Phase 3 carry nothing within it. This subdivision does not revise the interpretation given in Section 3.8.1, which already attributes the apparent decline to Delta dilution at these seventeen residues; it quantifies it. The within-phase contrast spans more than two orders of magnitude, from 0.044 in the Delta window to 7.20 in the latest.

Two limits apply. The windows are of unequal size, with 3a comprising 91.4% of the Phase 3 sample and 3c 2.4%, and per-state counts in 3c range from 4 to 287, so 3c should be read as a cross-state aggregate rather than per state. This under-representation of the later Omicron period is itself a finding: submission-volume-weighted sampling does not recover the 2022–2025 era in proportion to its duration, which is the sampling limitation stated in Section 2.2.2, quantified directly.

#### 3.15.2. Effect of F486 Inclusion on the ACE2 Panel

The seventeen-position ACE2 panel excludes F486, a principal escape position in later Omicron lineages (Section 2.6). To quantify the effect of that exclusion, panel burden was recomputed with F486 added as an eighteenth position (Table 4).

Inclusion of F486 raises Phase 3 panel burden by 0.020 substitutions per sequence and leaves Phases 1 and 2 unchanged. The effect is reported as a difference rather than as a revised absolute, because the comparison of interest is whether F486 alters the trajectory, and it does not. The trajectory shape is unaffected. The residual effect is concentrated in states with greater representation of the later Omicron window: F486 substitution frequency in Phase 3 ranges from 0.04% in Colorado, which contributes 8 sequences to window 3c, to 5.42% in Texas, which contributes 274. The exclusion of F486 is therefore conservative for Phase 3, as stated in Section 2.6, but immaterial at the aggregate level. The reason is the same sampling composition documented in Section 3.15.1: F486 substitutions are characteristic of the XBB-era lineages that dominate window 3c, and 3c contributes 1195 of the 50,000 Phase 3 sequences.

## 4. Conclusions

This study presents a comprehensive 13-parameter evolutionary analysis of SARS-CoV-2 across ten US states spanning the complete vaccination era (2020–2025) using a computational framework applied to 137,059 high-quality genomic sequences, with triplicate subsampling used to quantify subsampling stability as described in Section 4.3. The integrated analysis was designed to test four primary hypotheses regarding viral adaptation under sustained immune pressure, and to identify evolutionary patterns that single-parameter or single-region studies cannot resolve. The principal conclusions are summarized below.

### 4.1. Unique Insights from Multi-Parameter Cross-State Integration

Previous studies have predominantly focused on individual parameters, such as spike mutations alone, or have worked at country or global scale [18,19,88], capturing only a partial view of viral adaptation at subnational resolution. The integrated 13-parameter cross-state framework presented here yields several insights that are not visible from any single-parameter or single-region analysis. First, the convergence of ACE2 contact residue substitutions, furin cleavage site evolution, and N-glycosylation shifts across all ten states with diverse demographics, healthcare systems, and policy environments demonstrates that universal host–pathogen molecular pressures dominate over state-specific selective environments—a conclusion only achievable by joint cross-state cross-parameter examination. Second, the cross-parameter NSP16 paradox (high Phase 1 dN/dS combined with declining Phase 3 normalized mutation load) documents a “diversify-then-fix” evolutionary pattern that would be undetectable with either Parameter 12 or Parameter 13 alone. Third, the divergence between absolute and normalized mutation load rankings (Spike leads absolute load but ranks fourth by normalized load) reveals that small accessory proteins (NS7a, NS7b) carry far greater per-residue mutation pressure than Spike, a finding obscured by spike-centric analyses. Fourth, the simultaneous tracking of FUBAR site-specific selection, lineage temporal dynamics, and protein-domain mutation load across geographies uncovers convergent adaptive trajectories that establish viral evolution under vaccination as highly deterministic at the molecular level. Together, these findings demonstrate that the integrated multi-parameter cross-state approach provides a qualitatively different and more comprehensive view of SARS-CoV-2 adaptation than the sum of its components.

### 4.2. Confirmation of Primary Hypotheses

The four primary hypotheses stated in Section 1.4 were tested using the multi-parameter framework. The data support each hypothesis, with quantitative evidence summarized below.

**Hypothesis** **1** 
(CONFIRMED)**.** *Genome-wide mutation rates and dN/dS ratios were predicted to increase progressively across the three temporal phases in all ten US states. The data confirm this hypothesis: mutation rates rose monotonically from Phase 1 to Phase 3 in all ten states, and genome-wide dN/dS ratios rose monotonically in all ten states as well. This is consistent with intensifying immune-driven positive selection acting principally through lineage replacement: both metrics are computed as divergence from Wuhan-Hu-1, and the dominant lineage differs in every phase, so the phase-to-phase change reflects the substitution content of successively fitter prevailing lineages. The analysis does not separate this from any change in within-lineage selective pressure (Section 4.3).*

**Hypothesis** **2** 
(CONFIRMED)**.** *Ti/Tv ratios were predicted to decrease from Phase 1 to Phase 3, reflecting a declining relative contribution of host deaminase editing to the observed substitution spectrum. The data confirm this hypothesis: Ti/Tv ratios were lower in Phase 3 than in Phase 1 in all ten US states, with the population average falling from 3.69 ± 0.06 in Phase 1 to 2.18 ± 0.02 in Phase 2 and 2.11 ± 0.01 in Phase 3 (Table 1c). The decline was sharpest between Phases 1 and 2 in every state; five states showed a marginal Phase 2 to Phase 3 increase within the stabilized range, consistent with the new mutational equilibrium described in Section 3.3 rather than with continued decline. This 0.6-fold reduction reflects a declining relative contribution of transitions to accumulated differences, evidenced directly by the shift in dominant substitution types (C→T declining from 54.6% to 39.3% across phases). Because APOBEC3 and ADAR editing both generate transitions, the pattern indicates a declining relative contribution of these editing processes rather than a change in their character; C→T remains the single largest substitution class in Phase 3. As with Hypothesis 1, this shift is expressed through lineage replacement between phases, since the defining substitution set of each successively dominant lineage carries its own transition bias (Section 4.3).*

**Hypothesis** **3** 
(CONFIRMED)**.** *ACE2 contact residues, furin cleavage site positions, and N-glycosylation sites were predicted to show convergent evolution across geographically distinct states, consistent with universal immune pressure. The data strongly confirm this hypothesis: convergent substitutions at canonical ACE2 contact residues, the P681H/P681R furin cleavage transitions, and parallel glycan-shield reorganization were observed in all ten states, with the same positions recurring across geographies. Absolute mutation burdens at these sites varied between states with sampling composition (Section 3.8.1), but the identity of the positions under selection did not.*

**Hypothesis** **4** 
(CONFIRMED)**.** *Protein domain-specific mutation loads were predicted to be highest in spike and accessory proteins across all phases, with significant phase-dependent shifts in protein-wise dN/dS ranking. The data confirm this hypothesis: Spike led in absolute mutation load in all phases, the small accessory proteins NS7a and NS7b carried the highest per-residue mutation density in Phase 3 while ORF8 peaked in Phase 2 and fell to ninth by Phase 3, and the protein-wise dN/dS ranking shifted markedly across phases, with NSP12 (RdRp) and NSP16 occupying high-rank positions in early phases.*

Taken together, all four primary hypotheses are confirmed by the multi-parameter analysis. The convergence of evidence across genome-wide metrics (Hypotheses 1 and 2), functional sites (Hypothesis 3), and protein-domain partitioning (Hypothesis 4) establishes a coherent picture of vaccination-era SARS-CoV-2 evolution as highly reproducible across geographies and increasingly shaped by host immune pressure across the ten US states examined. The convergence observed is consistent with strong and largely shared selective constraints, but because the same lineages circulated nationally, it does not by itself establish independent evolutionary origin in each state (Section 4.3).

In addition to the four primary hypotheses, the framework’s amino acid physicochemical analysis (Parameter 11) reinforced this picture: charge- and hydrophobicity-altering substitutions accumulated preferentially at the same functional spike and RBD positions implicated above, indicating that the convergent immune-driven selection acted not only on which residues changed but on the physicochemical nature of those changes, consistent with directed antigenic remodeling rather than random drift.

### 4.3. Limitations and Future Work

This study has several limitations that suggest valuable directions for future research.

State-level vaccination rate correlation analysis: A particularly meaningful extension would be to correlate state-level vaccination rates and vaccine type distribution (mRNA vs. adenovirus-vectored vaccines) with the genomic evolutionary parameters reported here. The present study deliberately focused on viral genomic evolution as the primary outcome and did not incorporate state-level vaccination coverage data from CDC or state health departments. A follow-up study integrating per-state vaccination rates, booster uptake, and vaccine-type distribution with the 13-parameter framework would clarify the extent to which observed evolutionary patterns reflect heterogeneous immune pressure landscapes versus shared universal selective forces, and could identify whether high-vaccination states show distinct selection signatures compared to lower-vaccination states.

The ten state populations are geographically distinct sampling frames within a single national epidemic rather than evolutionarily independent replicates; the same lineages circulated across all ten states, so a mutation reaching high frequency in every state may reflect the spread of a dominant lineage as readily as parallel selection. Reproducibility across states therefore establishes that a signal is not an artifact of any one state’s surveillance, which is the inference drawn here, but it does not by itself demonstrate independent evolutionary origin. A further consideration concerns the dN/dS estimator. Both the genome-wide analysis (Section 3.2) and the protein-wise ranking (Section 3.13) use the unmodified Nei–Gojobori counting method, which classifies synonymous and non-synonymous sites assuming equal transition and transversion rates. The transition/transversion analysis (Section 3.3) shows that this assumption does not hold and is not constant across the study period, with κ declining from 3.69 in Phase 1 to 2.11 in Phase 3. Because transitions are disproportionately synonymous at third codon positions, the unmodified method undercounts synonymous sites and therefore understates dN/dS, and it does so more strongly where κ is higher. The reported values are consequently conservative, and the apparent Phase 1 to Phase 3 rise is somewhat larger than the underlying change; the direction of the trend is unaffected, and the Phase 3 crossing of neutrality is, if anything, more secure under correction. Recombination represents a further methodological consideration. Phase 3 encompasses recombinant lineages, most notably the XBB family and its descendants, and codon-based selection inference of the kind used for Parameters 2 and 4 assumes the absence of recombination within the analyzed alignment. Recombination can generate mosaic site patterns that inflate apparent rates of positive selection, and no formal recombination screening (for example, GARD) was applied prior to selection analysis. The site-specific selection signals reported here should therefore be interpreted with that caveat, particularly for Phase 3, and the extensive recombination landscape documented for SARS-CoV-2 [149] makes explicit recombination screening a priority refinement for subsequent work. Additional limitations include GISAID sequencing bias toward well-funded laboratories and high-income regions, the inability of population-level genomic surveillance data to fully resolve within-host evolutionary dynamics, the use of consensus sequences, which represent the dominant intra-host variant and cannot resolve minority quasispecies, and the focus on the United States, which limits generalizability to settings with different vaccine availability, healthcare infrastructure, or non-pharmaceutical intervention timelines. An equivalent multi-parameter analysis at country scale is a planned extension of this work. A caveat specific to Parameter 4 arises from the alignment preparation it requires. Internal stop codons were replaced with NNN before selection analysis, which is required for HyPhy but removes premature terminations from the alignment. ORF8 acquires exactly such a truncation in Alpha (Q27*), so the ORF8 selection results characterize codon-level selection in sequences whose stop codons have been masked, and the loss-of-function interpretation drawn from them rests on the wider literature and on the diversifying-then-declining trajectory rather than on direct detection of truncation. The pooling of triplicates for this parameter also means the site-level selection results carry no replicate-based dispersion estimate; variation reported for Parameter 4 is across locations, not across subsampling replicates. A further caveat applies specifically to Parameter 9. The sequon scan requires all three residues of an N-X-S/T motif to be unambiguously translated, so where low coverage or a deletion mapped into reference coordinates renders any of the three positions ambiguous, the sequon is scored as absent rather than as undetermined. Some fraction of the net Phase 3 glycan loss reported in Section 3.10 may therefore reflect incomplete sequence determination rather than genuine sequon loss, and the bias runs in the direction of overestimating loss. The principal Phase 3 result—near-universal loss of the N17 sequon through the T19I/R coding substitution—does not depend on ambiguous positions and is unaffected, but the absolute magnitude of the net loss should be read as an upper bound. Future work integrating population-level genomic surveillance with within-host longitudinal data and clinical metadata will further strengthen the inferential framework presented here.

A consideration of scale applies to the genome-wide metrics. Parameters 1, 2, 3, and 12 are computed as pairwise divergence from Wuhan-Hu-1, averaged across a sample whose dominant lineage differs in each phase: ancestral and B.1 lineages in Phase 1, Alpha in Phase 2, and Delta with an early-Omicron contribution in Phase 3. The defining substitutions of each of those lineages reached high frequency because they conferred a transmission or immune-escape advantage, so the phase contrasts reported here capture selection as it acted through lineage replacement, which was the principal mode of SARS-CoV-2 adaptation over this period. What the design does not separate is the intensity of selection operating within a lineage from the effect of which lineage prevailed; a phase in which a heavily substituted lineage dominates will register a higher mutation rate and dN/dS than one in which a less divergent lineage does, independently of any change in within-lineage selective pressure. The two components are both genuine evolutionary signals and are not separated by the analysis presented here. Section 3.6.2 and Section 3.9.4 apply the same reasoning at the level of individual substitutions, attributing the apparent decline of N501Y and the low Phase 3 share of the NSHRRAR motif to lineage composition rather than to reversion. The magnitude of this effect is directly measurable within Phase 3: across the ten states, the early-Omicron fraction of the sample correlates with genome-wide mutation rate at r = +0.95 and with mutation load at r = +0.97, while correlating with genome-wide dN/dS at r = −0.83 (Section 3.2). Composition therefore shifts these metrics substantially even with calendar time held constant. Stratifying these parameters within the dominant lineage of each phase would partition the two contributions and would establish whether the observed trends persist at constant lineage background; the lineage assignments required are already present in the Parameter 10 outputs, and this is identified as a natural extension of the framework rather than a correction to it.

### 4.4. Closing Statement

In summary, this 13-parameter cross-state evolutionary analysis confirms all four primary hypotheses and provides a comprehensive, integrated view of SARS-CoV-2 adaptation under sustained population immune pressure across the United States from 2020 through 2025. Vaccination coverage, vaccine type, and prior-infection history were not measured in this study, so the immune pressure referred to here is the aggregate of vaccine-induced, infection-induced, and hybrid immunity rather than a vaccine-specific effect. The findings establish that viral evolution during the vaccination era has been highly convergent at the molecular level, dominated by universal host–pathogen pressures rather than state-specific selective environments, and characterized by distinct phase-dependent shifts in selection regimes across structural and accessory proteins. The integrated multi-parameter cross-state framework presented here provides a methodological template for future surveillance of SARS-CoV-2 and other emerging RNA viruses, and identifies specific molecular targets (notably NSP7 conservation, NSP12 high mutation accumulation, and the NSP16 diversify-then-fix pattern) for next-generation antiviral and vaccine development.

## Figures and Tables

**Figure 3:**
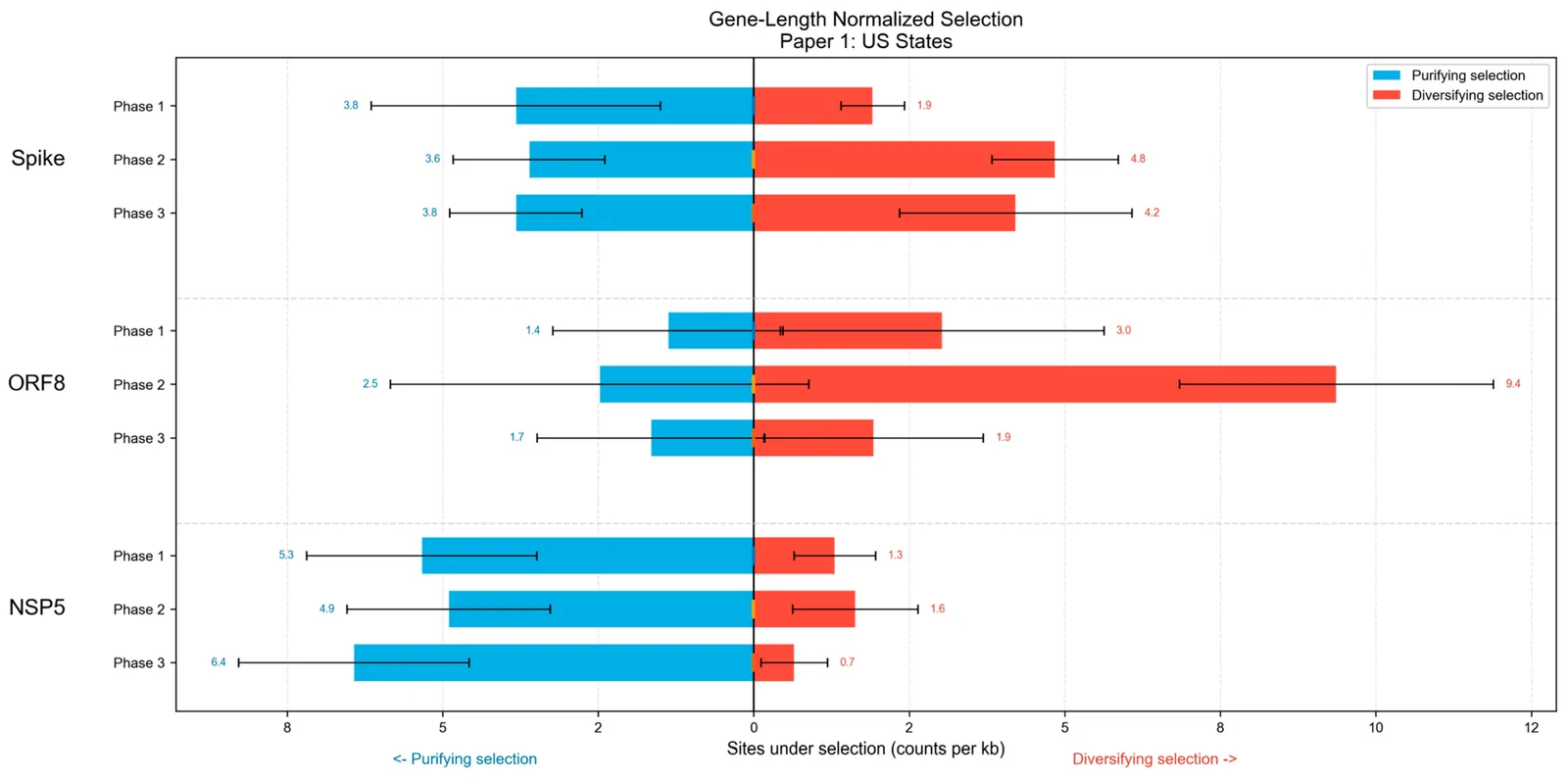
Gene-length normalized FUBAR selection analysis for Spike, ORF8, and NSP5 across 10 US states. Butterfly chart showing purifying selection (light blue, extending left) and diversifying selection (red, extending right) in counts per kilobase. Bars = mean across 10 states; error bars = ± SD. Zero center line separates selection types. Each gene shows Phase 1 (January 2020–December 2020), Phase 2 (January 2021–June 2021), Phase 3 (July 2021–December 2025).

**Figure 4:**
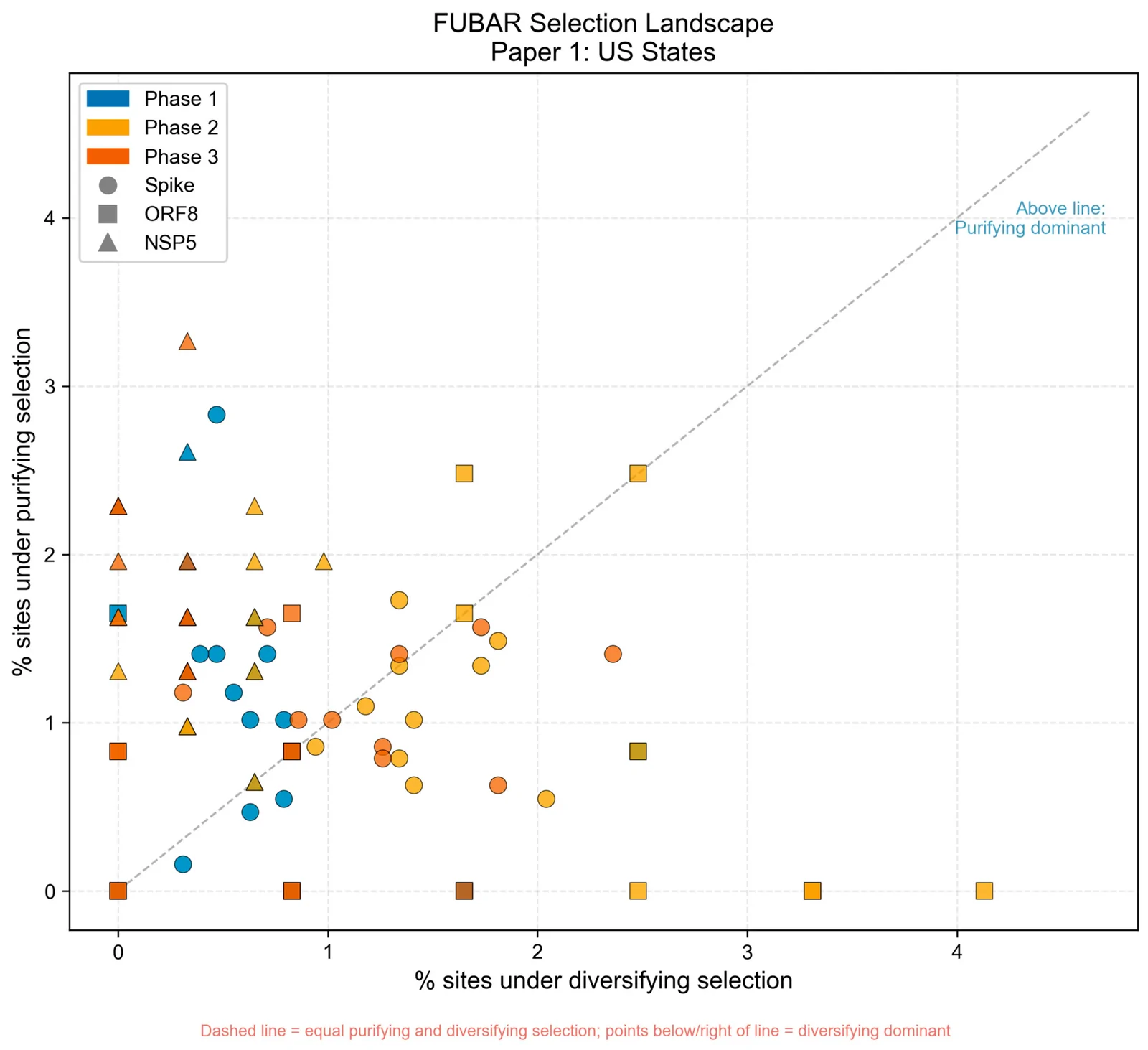
FUBAR selection landscape for Spike (●), ORF8 (■), and NSP5 (▲) across 10 US states. Each dot = one state in one phase (n = 90 total). Dashed diagonal = equal diversifying and purifying selection. Color: blue = Phase 1, amber = Phase 2, vermillion = Phase 3.

**Figure 5:**
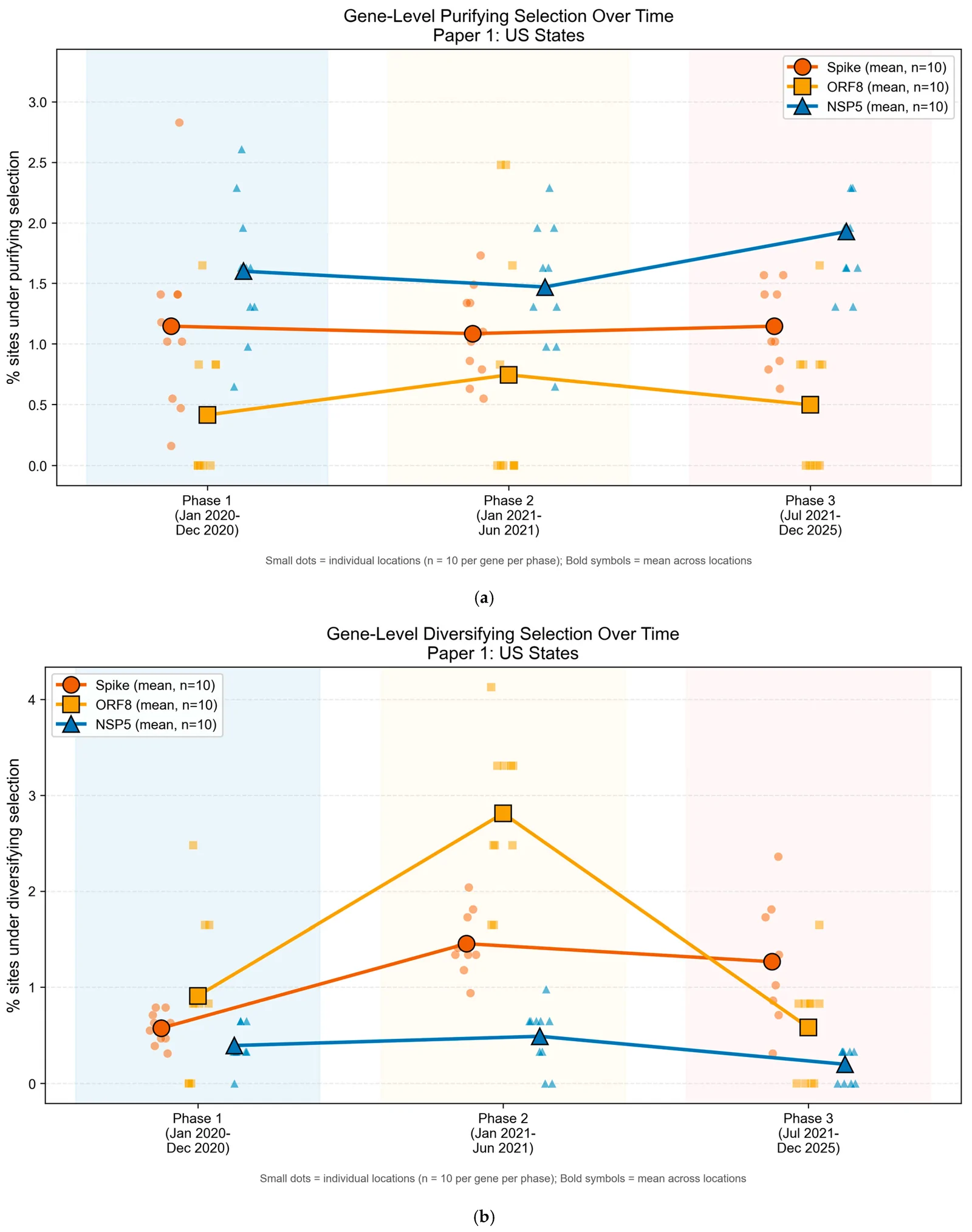
(**a**) Gene-level purifying selection over time for Spike (●), ORF8 (■), and NSP5 (▲) across 10 US states. Bold symbols = mean across 10 states (n = 10 per gene per phase); small dots = individual state values. Era-shaded backgrounds: blue = pre-vaccination, yellow = early vaccination, pink = sustained immune pressure. (**b**) Gene-level diversifying selection over time. Same format as Figure 5a. Note the dramatic ORF8 diversifying spike in Phase 2 (mean = 2.81%) followed by collapse in Phase 3 (0.58%), consistent with evolutionary abandonment. Spike diversifying selection increases while NSP5 remains near zero.

**Figure 6:**
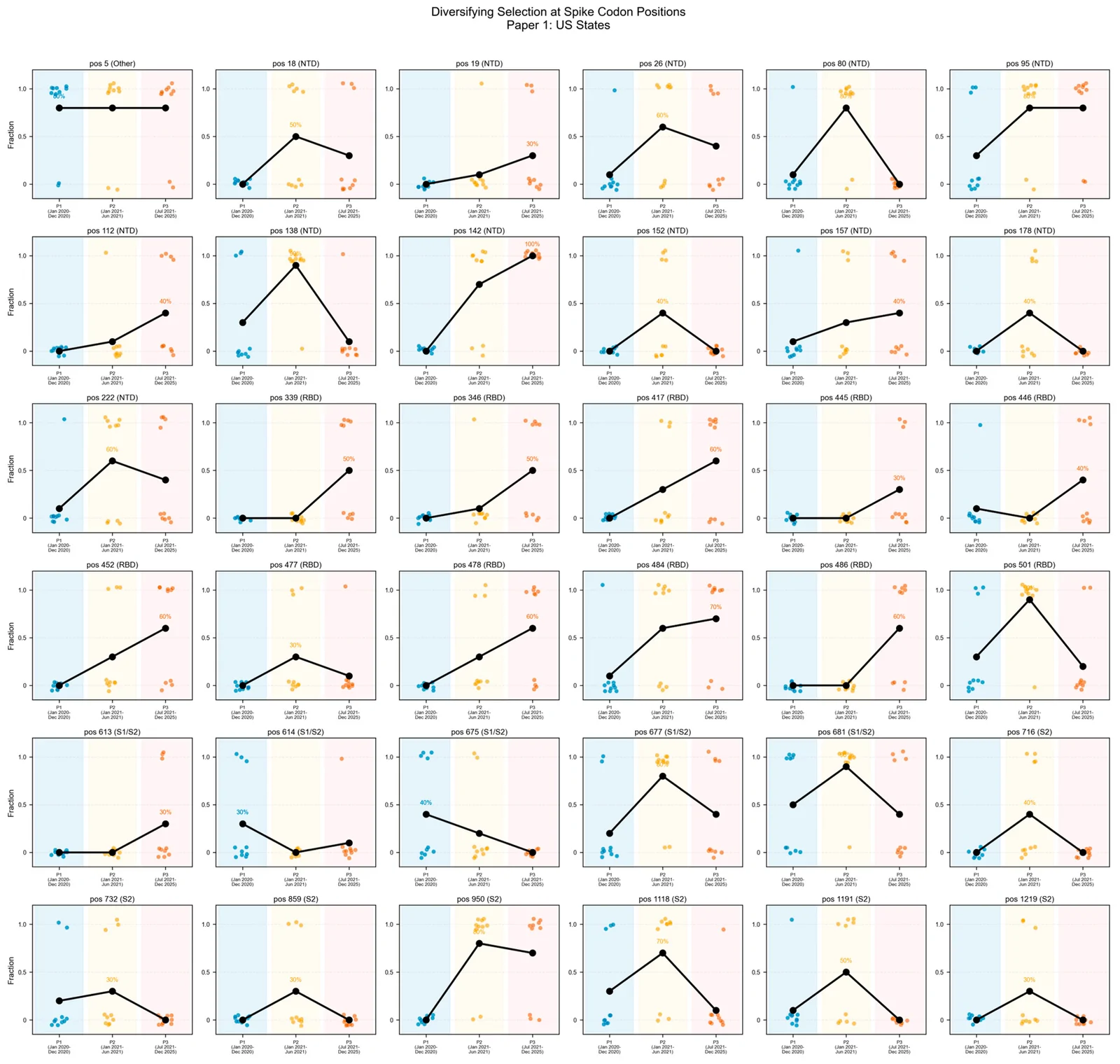
Convergent diversifying selection at 36 Spike codon positions across 10 US states. Each panel = one codon position. Colored dots = individual state detection (1 = detected, 0 = not detected, with jitter); bold black line = convergence frequency (fraction of 10 states). Phase-shaded backgrounds: blue = P1 (January 2020–December 2020), yellow = P2 (January 2021–June 2021), pink = P3 (July 2021–December 2025). Percentage = peak convergence.

**Figure 7:**
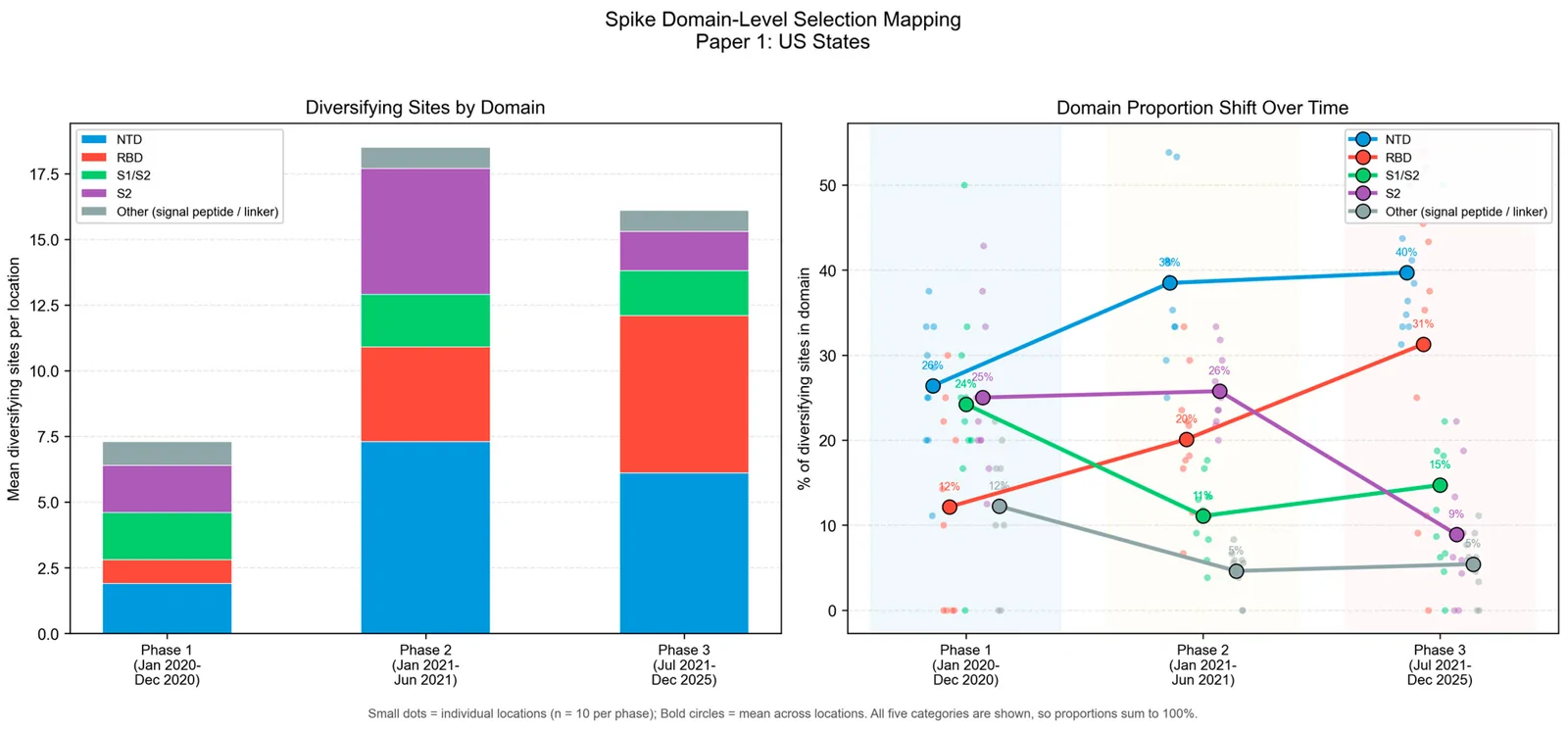
Spike domain-level selection mapping across 10 US states. (**Left**) stacked bars showing mean diversifying sites per location by domain (NTD, RBD, S1/S2, S2, Other). (**Right**) temporal domain proportion shift; bold circles = pooled proportion across 10 states, small dots = individual state proportions. RBD triples from 12% (P1) to 37% (P3); S2 declines from 25% to 9%.

**Figure 8:**
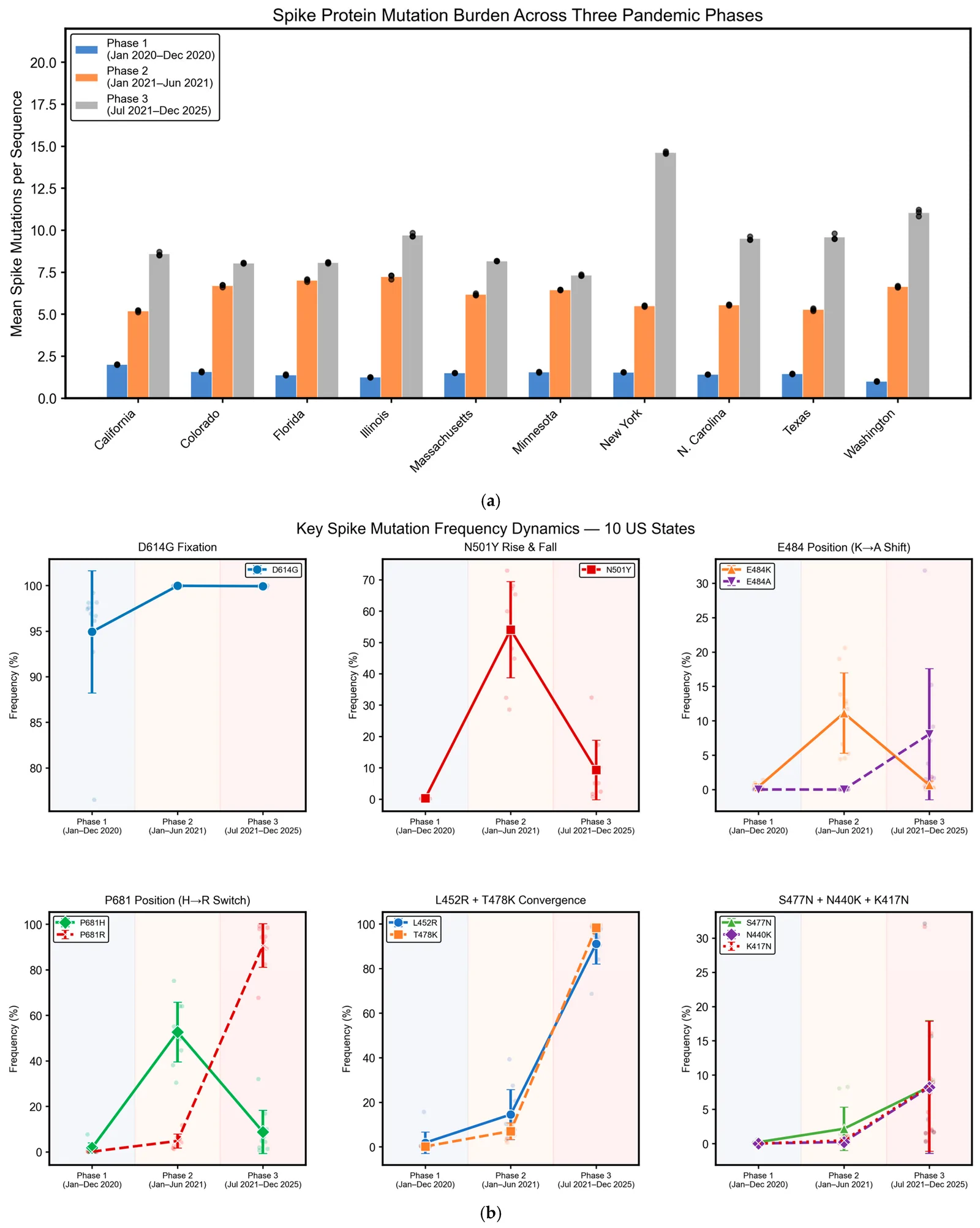

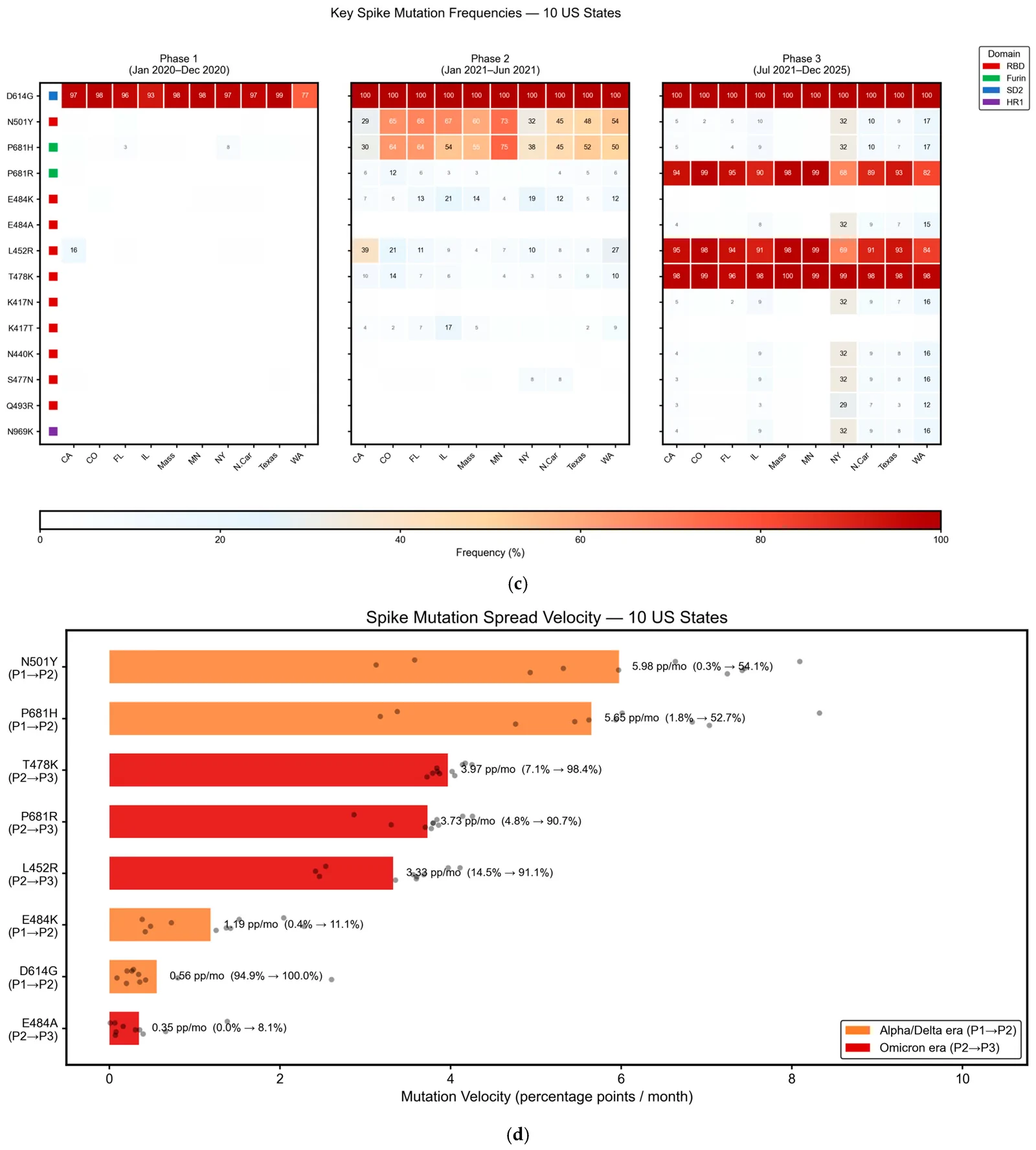

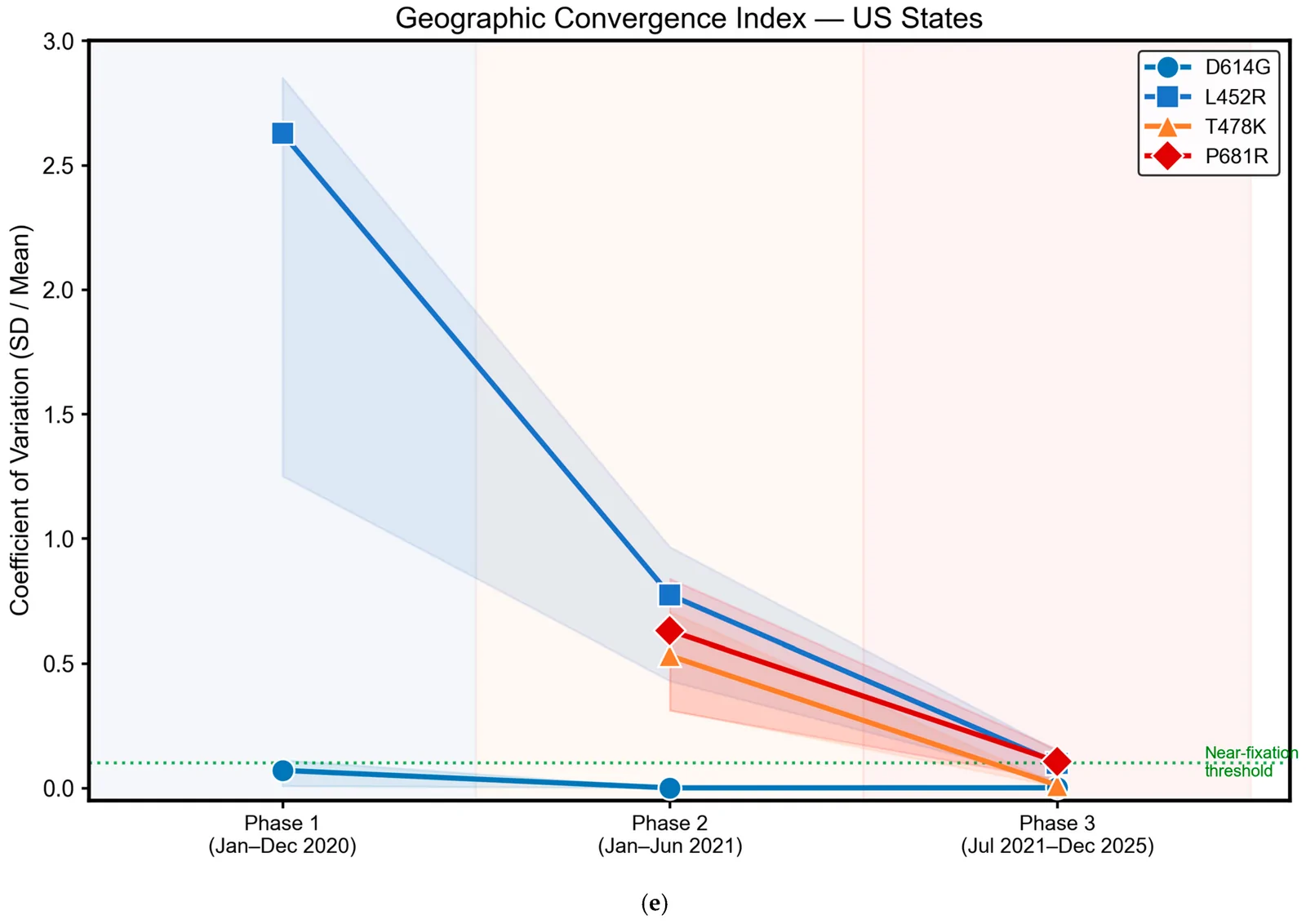
(**a**) Spike protein mutation burden across three pandemic phases for 10 US states. Blue = Phase 1 (January–December 2020), Orange = Phase 2 (January–June 2021), Gray = Phase 3 (July 2021–December 2025). Black dots = individual triplicate values. (**b**) Key Spike mutation frequency dynamics across 10 US states. Bold symbols = cross-location mean. Faint dots = individual location values. Error bars = ± SD. Background shading: blue = pre-vaccination, orange = early vaccination, red = sustained immune pressure era. (**c**) Mutation frequency heatmap across 10 US states. Rows = 14 tracked mutations. Columns = locations. Three panels = three pandemic phases. Color intensity proportional to frequency (%). Left margin squares indicate functional domain. (**d**) Mutation spread velocity across 10 US states. Orange bars = Alpha/Delta era (P1→P2). Red bars = Omicron era (P2→P3). Black/gray dots = per-location velocities. Annotations show absolute frequency change. (**e**) Geographic Convergence Index across 10 US states. Each line = one mutation tracked over time. Shaded bands = 95% bootstrap confidence intervals. Green dashed line = low-dispersion threshold (CV < 0.10), labeled “near-fixation threshold” in the plot; the coefficient of variation measures between-state dispersion, not fixation, and the low-dispersion reading is the correct one. Declining lines indicate convergent evolution.

**Figure 9:**
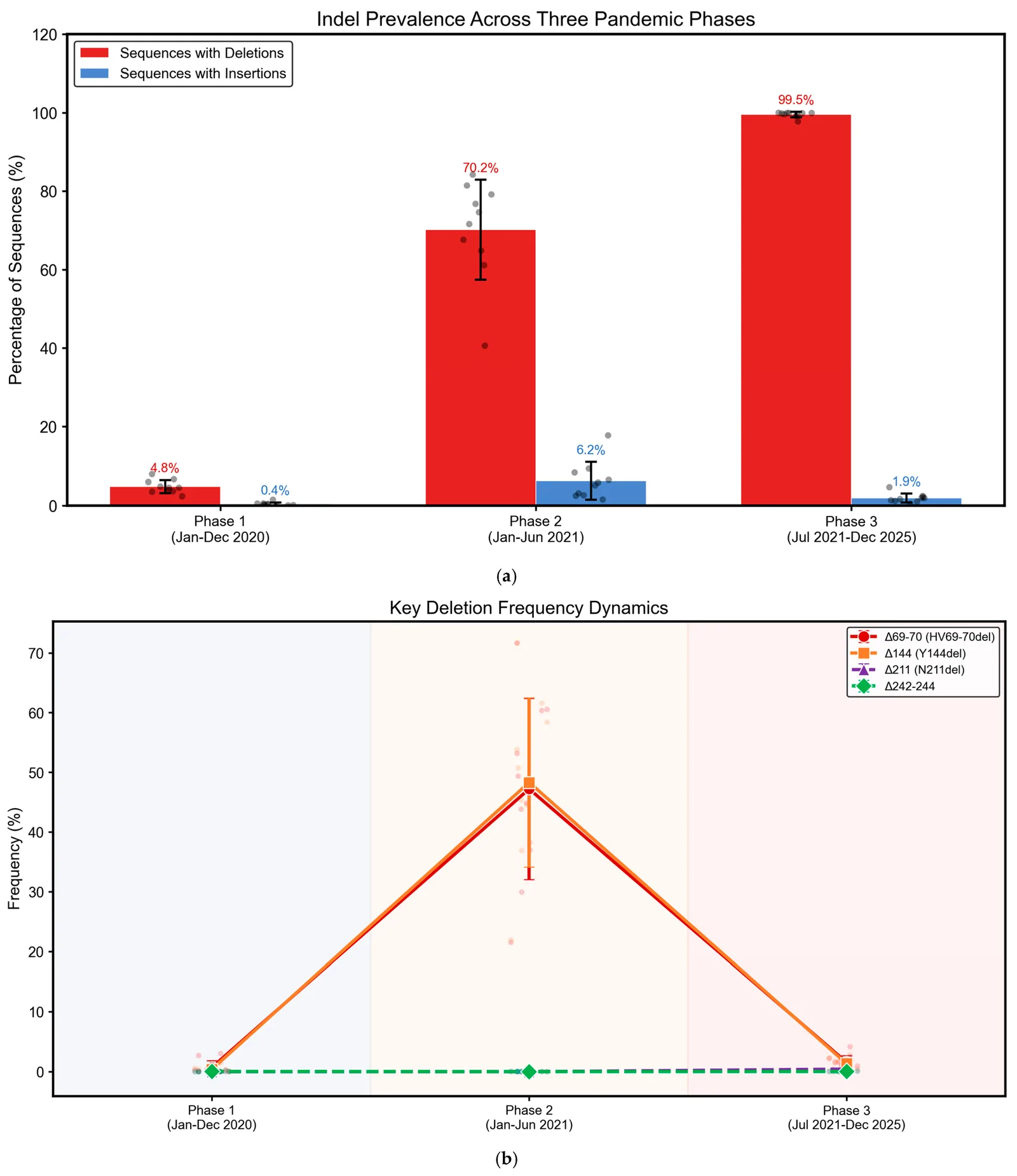

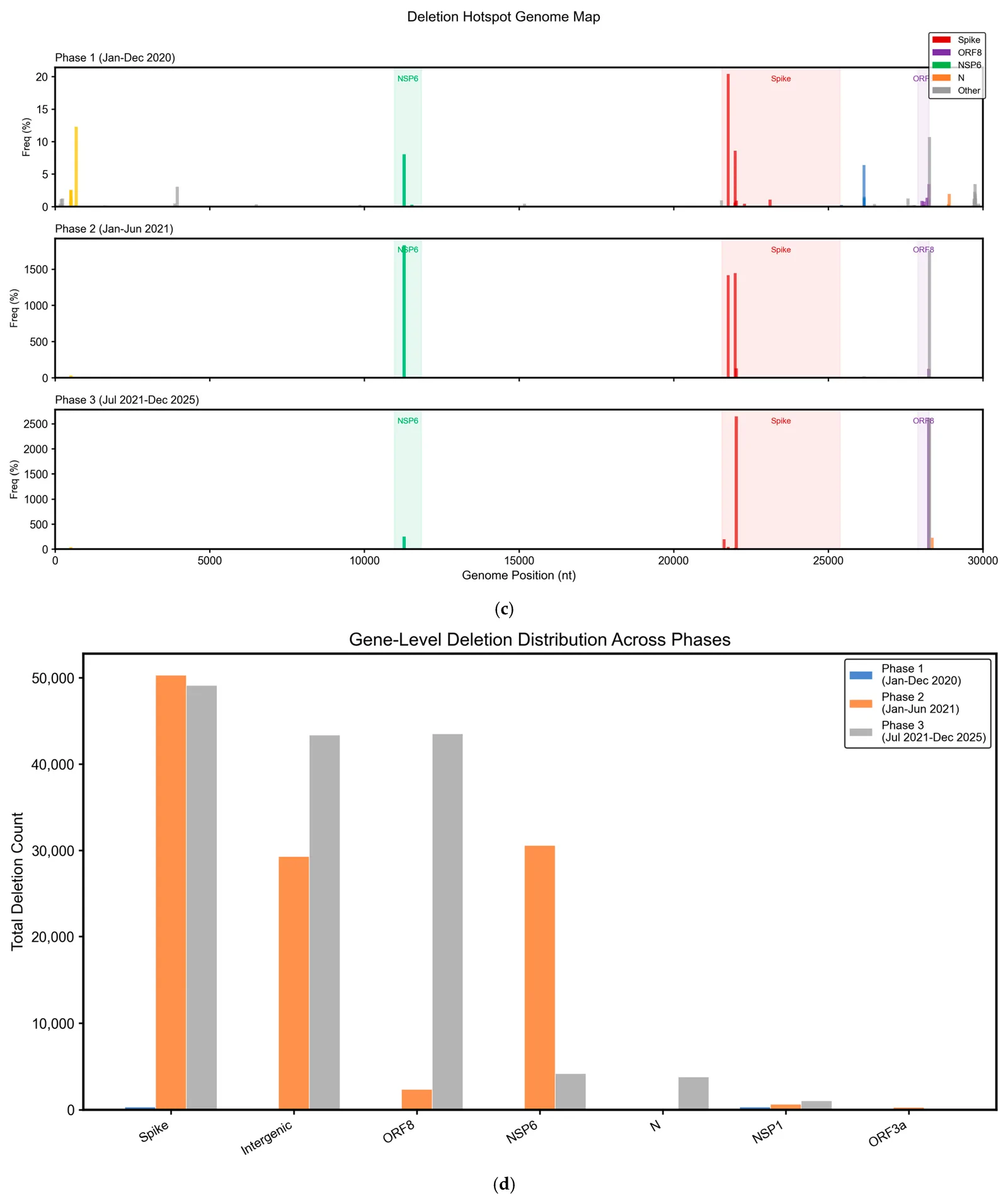
(**a**) Indel prevalence across three pandemic phases for 10 US states. Red bars = percentage of sequences with at least one deletion. Blue bars = percentage with at least one insertion. Black dots = individual location means. Error bars = ± SD. (**b**) Key deletion frequency dynamics across 10 US states. Four Spike deletions are shown; the ORF8 Δ25–27 deletion has been withdrawn from this figure because the 50% reciprocal-overlap detection criterion cannot resolve it. Bold symbols = cross-location mean. Faint dots = individual location values. Error bars = ± SD. Background shading: blue = pre-vaccination, orange = early vaccination, red = sustained immune pressure era. (**c**) Deletion hotspot genome map across 10 US states. Three panels = Phase 1, Phase 2, Phase 3. Bar height = cumulative deletion frequency at each genome position. Colors: Spike (red), ORF8 (purple), NSP6 (green), N (orange), Other (gray). (**d**) Gene-level deletion distribution across 10 US states. Grouped bars show total deletion count per gene across three phases. Blue = Phase 1, Orange = Phase 2, Gray = Phase 3.

**Table 3:** Phase 3 partitioned by collection date. Burdens are amino acid substitutions per sequence. The partition is validated against two independently reported quantities: weighted by n, the three windows reconstruct the Phase 3 Spike burden of 12.51 reported in Section 3.14 to three significant figures, and the Phase 3 ACE2 panel burden of 0.614 to within 2%.

Window	Period	n	Dominant Lineages	Spike Burden	ACE2 Panel Burden
3a	July–December 2021	45,704	AY.103 19.3%, AY.44 15.1%, AY.3 10.6%	10.59	0.044
3b	January–June 2022	3101	BA.2.12.1 46.9%, BA.2 23.6%	30.83	6.65
3c	July 2022 onward	1195	XBB.1.5 13.4%, BA.2.12.1 10.0%, BA.5.2.1 3.7%	38.57	7.20

**Table 4:** ACE2 panel burden with and without F486. Panel-17 values are those reported in Section 3.8. The F486 difference was computed by re-scoring the same cohort against an eighteen-position panel; the recomputation reproduced the Section 3.8 panel-17 values to within 2% at every phase.

Phase	17-Position Panel	18 Positions (+F486)	Difference	Sequences Carrying F486
Phase 1	0.021	0.021	+0.000	0.01%
Phase 2	0.739	0.739	+0.000	0.01%
Phase 3	0.614	0.634	+0.020	1.97%

## Data Availability

The SARS-CoV-2 genome sequences analyzed in this study are available from the GISAID EpiCoV database (www.gisaid.org), subject to the GISAID Database Access Agreement. The specific EPI_ISL accession numbers for all 137,059 analyzed sequences are provided in Supplementary Table S2; sequences were retrieved between 25 and 27 February 2026. The Wuhan-Hu-1 reference genome (NC_045512.2) is available from NCBI GenBank. All analysis code supporting the findings of this study is available from the corresponding author on request and is deposited in a public repository with a citable DOI. Full details of the reproducibility procedure are given in Section 2.9.

## Supplementary Materials

The following supporting information can be downloaded at: https://www.mdpi.com/article/10.3390/bioengineering13101136/s1, Table S1: Mutation type proportions by state and phase—C→T, G→T, A→G and G→A percentages for each of the ten US states across the three pandemic phases, the location-resolved values underlying Table 2; Table S2: GISAID EPI_ISL accession numbers for all 137,059 analyzed sequences, with state, phase, triplicate, collection date, Pango lineage, clade and sequencing technology for each sequence.

## References

[1] Zhu N., Zhang D., Wang W., Li X., Yang B., Song J., Zhao X., Huang B., Shi W., Lu R. (2020). A novel coronavirus from patients with pneumonia in China, 2019. N. Engl. J. Med..

[2] World Health Organization (2020). Director-General’s Opening Remarks at the Media Briefing on COVID-19, 11 March 2020.

[3] Centers for Disease Control and Prevention (2024). COVID-19 Data Tracker.

[4] Wu F., Zhao S., Yu B., Chen Y.-M., Wang W., Song Z.-G., Hu Y., Tao Z.-W., Tian J.-H., Pei Y.-Y. (2020). A new coronavirus associated with human respiratory disease in China. Nature.

[5] Kim D., Lee J.Y., Yang J.S., Kim J.W., Kim V.N., Chang H. (2020). The architecture of SARS-CoV-2 transcriptome. Cell.

[6] Smith E.C., Denison M.R. (2013). Coronaviruses as DNA wannabes: A new model for the regulation of RNA virus replication fidelity. PLoS Pathog..

[7] Robson F., Khan K.S., Le T.K., Paris C., Demirbag S., Barfuss P., Rocchi P., Ng W.-L. (2020). Coronavirus RNA proofreading: Molecular basis and therapeutic targeting. Mol. Cell.

[8] Sender R., Bar-On Y.M., Gleizer S., Bernshtein B., Flamholz A., Phillips R., Milo R. (2021). The total number and mass of SARS-CoV-2 virions. Proc. Natl. Acad. Sci. USA.

[9] Faria N.R., Mellan T.A., Whittaker C., Claro I.M., Candido D.D.S., Mishra S., Crispim M.A.E., Sales F.C.S., Hawryluk I., McCrone J.T. (2021). Genomics and epidemiology of the P.1 SARS-CoV-2 lineage in Manaus, Brazil. Science.

[10] Hattab D., Amer M.F.A., Al-Alami Z.M., Bakhtiar A. (2024). SARS-CoV-2 journey: From alpha variant to omicron and its sub-variants. Infection.

[11] Rambaut A., Holmes E.C., O’Toole A., Hill V., McCrone J.T., Ruis C., du Plessis L., Pybus O.G. (2020). A dynamic nomenclature proposal for SARS-CoV-2 lineages to assist genomic epidemiology. Nat. Microbiol..

[12] Davies N.G., Abbott S., Barnard R.C., Jarvis C.I., Kucharski A.J., Munday J.D., Pearson C.A.B., Russell T.W., Tully D.C., Washburne A.D. (2021). Estimated transmissibility and impact of SARS-CoV-2 lineage B.1.1.7 in England. Science.

[13] Planas D., Veyer D., Baidaliuk A., Staropoli I., Guivel-Benhassine F., Rajah M.M., Planchais C., Porrot F., Robillard N., Puech J. (2021). Reduced sensitivity of SARS-CoV-2 variant Delta to antibody neutralization. Nature.

[14] Viana R., Moyo S., Amoako D.G., Tegally H., Scheepers C., Althaus C.L., Anyaneji U.J., Bester P.A., Boni M.F., Chand M. (2022). Rapid epidemic expansion of the SARS-CoV-2 Omicron variant in southern Africa. Nature.

[15] Koyama T., Platt D., Parida L. (2020). Variant analysis of SARS-CoV-2 genomes. Bull. World Health Organ..

[16] Rochman N.D., Wolf Y.I., Faure G., Mutz P., Zhang F., Koonin E.V. (2021). Ongoing global and regional adaptive evolution of SARS-CoV-2. Proc. Natl. Acad. Sci. USA.

[17] Cao Y., Wang J., Jian F., Xiao T., Song W., Yisimayi A., Huang W., Li Q., Wang P., An R. (2022). Omicron escapes the majority of existing SARS-CoV-2 neutralizing antibodies. Nature.

[18] Gupta S., Gupta D., Bhatnagar S. (2024). Analysis of SARS-CoV-2 genome evolutionary patterns. Microbiol. Spectr..

[19] Gayvert K., McKay S., Lim W.K., Baum A., Kyratsous C., Copin R., Atwal G.S. (2023). Evolutionary trajectory of SARS-CoV-2 genome shifts during widespread vaccination and emergence of Omicron variant. npj Viruses.

[20] Khurana M.P., Curran-Sebastian J., Scheidwasser N., Morgenstern C., Rasmussen M., Fonager J., Stegger M., Tang M.-H.E., Juul J.L., Escobar-Herrera L.A. (2024). High-resolution epidemiological landscape from ~290,000 SARS-CoV-2 genomes from Denmark. Nat. Commun..

[21] Lemey P., Ruktanonchai N., Hong S.L., Colizza V., Poletto C., Broeck F.V.D., Gill M.S., Ji X., Levasseur A., Munnink B.B.O. (2021). Untangling introductions and persistence in COVID-19 resurgence in Europe. Nature.

[22] Carabelli A.M., Peacock T.P., Thorne L.G., Harvey W.T., Hughes J., Peacock S.J., Barclay W.S., de Silva T.I., Towers G.J., COVID-19 Genomics UK Consortium (2023). SARS-CoV-2 variant biology: Immune escape, transmission, and fitness. Nat. Rev. Microbiol..

[23] Duchene S., Featherstone L., Haritopoulou-Sinanidou M., Rambaut A., Lemey P., Baele G. (2020). Temporal signal and the phylodynamic threshold of SARS-CoV-2. Virus Evol..

[24] Hadfield J., Megill C., Bell S.M., Huddleston J., Potter B., Callender C., Sagulenko P., Bedford T., Neher R.A. (2018). Nextstrain: Real-time tracking of pathogen evolution. Bioinformatics.

[25] van Dorp L., Acman M., Richard D., Shaw L.P., Ford C.E., Ormond L., Owen C.J., Pang J., Tan C.C.S., Boshier F.A.T. (2020). Emergence of genomic diversity and recurrent mutations in SARS-CoV-2. Infect. Genet. Evol..

[26] Cao Y., Jian F., Wang J., Yu Y., Song W., Yisimayi A., Wang J., An R., Chen X., Zhang N. (2023). Imprinted SARS-CoV-2 humoral immunity induces convergent Omicron RBD evolution. Nature.

[27] Wang Q., Guo Y., Iketani S., Nair M.S., Li Z., Mohri H., Wang M., Yu J., Bowen A.D., Chang J.Y. (2022). Antibody evasion by SARS-CoV-2 Omicron subvariants BA.2.12.1, BA.4, and BA.5. Nature.

[28] Yang S., Yu Y., Xu Y., Jian F., Song W., Yisimayi A., Wang P., Wang J., Liu J., Yu L. (2024). Fast evolution of SARS-CoV-2 BA.2.86 to JN.1 under heavy immune pressure. Lancet Infect. Dis..

[29] Turakhia Y., Thornlow B., Hinrichs A.S., De Maio N., Gozashti L., Lanfear R., Haussler D., Corbett-Detig R. (2021). Ultrafast sample placement on existing trees (UShER) enables real-time phylogenetics for the SARS-CoV-2 pandemic. Nat. Genet..

[30] Haddox H.K., Angehrn G., Sesta L., ennings-Shaffer C., Temple S.D., Galloway J.G., Hinrichs A.S., DeWitt W.S., Bloom J.D., Iv F.A.M. (2025). The mutation rate of SARS-CoV-2 is highly variable between sites and is influenced by sequence context, genomic region, and RNA structure. Nucleic Acids Res..

[31] Amicone M., Borges V., Alves M.J., Isidro J., Zé-Zé L., Duarte S., Vieira L., Guiomar R., Gomes J.P., Gordo I. (2022). Mutation rate of SARS-CoV-2 and emergence of mutators during experimental evolution. Evol. Med. Public Health.

[32] Ma W., Zhang K., Cao Y., Li M. (2025). SARS-CoV-2 keeps evolving, so must our research efforts. Natl. Sci. Rev..

[33] Kryazhimskiy S., Plotkin J.B. (2008). The population genetics of dN/dS. PLoS Genet.

[34] Yang Z., Nielsen R., Goldman N., Pedersen A.M. (2000). Codon-substitution models for heterogeneous selection pressure at amino acid sites. Genetics.

[35] Murrell B., Wertheim J.O., Moola S., Weighill T., Scheffler K., Kosakovsky Pond S.L. (2012). Detecting individual sites subject to episodic diversifying selection. PLoS Genet..

[36] Maiti A.K. (2024). Progressive evolutionary dynamics of gene-specific ω led to the emergence of novel SARS-CoV-2 strains having super-infectivity and virulence with vaccine neutralization. Int. J. Mol. Sci..

[37] Liu Y. (2025). Is SARS-CoV-2 facing constraints in its adaptive evolution?. Biomol. Biomed..

[38] Ghafari M., Kemp S.A., Hall M., Clarke J., Ferretti L., Thomson L., Studley R., Walker A.S., The COVID-19 Infection Survey Group, The COVID-19 Genomics UK (COG-UK) Consortium (2025). SARS-CoV-2 genomic diversity and within-host evolution in individuals with persistent infection in the UK: An observational, longitudinal, population-based surveillance study. Lancet Microbe.

[39] Xia X., Yuen K.Y. (2005). Differential selection and mutation between dsDNA and ssDNA phages shape the evolution of their genomic AT percentage. BMC Genet..

[40] Simmonds P. (2020). Rampant C→U hypermutation in the genomes of SARS-CoV-2 and other coronaviruses: Causes and consequences for their short- and long-term evolutionary trajectories. mSphere.

[41] Simmonds P., Ansari M.A. (2021). Extensive C→U transition biases in the genomes of a wide range of mammalian RNA viruses; potential associations with transcriptional mutations, damage- or host-mediated editing of viral RNA. PLoS Pathog..

[42] Nei M., Kumar S. (2000). Molecular Evolution and Phylogenetics.

[43] Harvey W.T., Carabelli A.M., Jackson B., Gupta R.K., Thomson E.C., Harrison E.M., Ludden C., Reeve R., Rambaut A., COVID-19 Genomics UK (COG-UK) Consortium (2021). SARS-CoV-2 variants, spike mutations, and immune escape. Nat. Rev. Microbiol..

[44] Simmonds P. (2024). C→U transition biases in SARS-CoV-2: Still rampant 4 years from the start of the COVID-19 pandemic. mBio.

[45] Di Giorgio S., Martignano F., Torcia M.G., Mattiuz G., Conticello S.G. (2020). Evidence for host-dependent RNA editing in the transcriptome of SARS-CoV-2. Sci. Adv..

[46] Azgari C., Kilinc Z., Turhan B., Circi D., Adebali O. (2021). The mutation profile of SARS-CoV-2 is primarily shaped by the host antiviral defense. Viruses.

[47] Murrell B., Moola S., Mabona A., Weighill T., Sheward D., Pond S.L.K., Scheffler K. (2013). FUBAR: A fast, unconstrained Bayesian approximation for inferring selection. Mol. Biol. Evol..

[48] Kosakovsky Pond S.L., Poon A.F.Y., Velazquez R., Weaver S., Hepler N.L., Murrell B., Shank S.D., Magalis B.R., Bouvier D., Nekrutenko A. (2020). HyPhy 2.5—A customizable platform for evolutionary hypothesis testing using phylogenies. Mol. Biol. Evol..

[49] Kosakovsky Pond S.L., Frost S.D.W., Muse S.V. (2005). HyPhy: Hypothesis testing using phylogenies. Bioinformatics.

[50] Martin D.P., Lytras S., Lucaci A.G., Maier W., Grüning B., Shank S.D., Weaver S., MacLean O.A., Orton R.J., Lemey P. (2022). Selection analysis identifies clusters of unusual mutational changes in Omicron lineage BA.1 that likely impact spike function. Mol. Biol. Evol..

[51] Oliver J.L., Bernaola-Galván P., Carpena P., Perfectti F., Gómez-Martín C., Castiglione S., Raia P., Verdú M., Moya A. (2025). Strong evidence for the evolution of decreasing compositional heterogeneity in SARS-CoV-2 genomes during the pandemic. Sci. Rep..

[52] Essabbar A., El Mazouri S., Boumajdi N., Bendani H., Aanniz T., Mouna O., Lahcen B., Ibrahimi A. (2024). Temporal dynamics and genomic landscape of SARS-CoV-2 after four years of evolution. Cureus.

[53] Watanabe Y., Allen J.D., Wrapp D., McLellan J.S., Crispin M. (2020). Site-specific glycan analysis of the SARS-CoV-2 spike. Science.

[54] Walls A.C., Park Y.J., Tortorici M.A., Wall A., McGuire A.T., Veesler D. (2020). Structure, function, and antigenicity of the SARS-CoV-2 spike glycoprotein. Cell.

[55] Wrapp D., Wang N., Corbett K.S., Goldsmith J.A., Hsieh C.-L., Abiona O., Graham B.S., McLellan J.S. (2020). Cryo-EM structure of the 2019-nCoV spike in the prefusion conformation. Science.

[56] Korber B., Fischer W.M., Gnanakaran S., Yoon H., Theiler J., Abfalterer W., Hengartner N., Giorgi E.E., Bhattacharya T., Foley B. (2020). Tracking changes in SARS-CoV-2 spike: Evidence that D614G increases infectivity of the COVID-19 virus. Cell.

[57] Yurkovetskiy L., Wang X., Pascal K.E., Tomkins-Tinch C., Nyalile T., Wang Y., Baum A., Diehl W.E., Dauphin A., Carbone C. (2020). Structural and functional analysis of the D614G SARS-CoV-2 spike protein variant. Cell.

[58] Zhang L., Jackson C.B., Mou H., Ojha A., Peng H., Quinlan B.D., Rangarajan E.S., Pan A., Vanderheiden A., Suthar M.S. (2020). SARS-CoV-2 spike-protein D614G mutation increases virion spike density and infectivity. Nat. Commun..

[59] Ito J., Suzuki R., Uriu K., Itakura Y., Zahradnik J., Kimura K.T., Deguchi S., Wang L., Lytras S., Tamura T. (2023). Convergent evolution of SARS-CoV-2 Omicron subvariants leading to the emergence of BQ.1.1 variant. Nat. Commun..

[60] Thomas S.V., Dienger-Stambaugh K., Jordan M., Wang Y., Hammonds J., Spearman P., Shi D. (2023). Inactivation of SARS-CoV-2 on surfaces by cold-plasma-generated reactive species. Bioengineering.

[61] Thomas S.V., Wang Y., Shi D. (2025). Biomedical applications of cold atmospheric plasma: Cancer therapy and virus inactivation. Nano LIFE.

[62] McCarthy K.R., Rennick L.J., Nambulli S., Robinson-McCarthy L.R., Bain W.G., Haidar G., Duprex W.P. (2021). Recurrent deletions in the SARS-CoV-2 spike glycoprotein drive antibody escape. Science.

[63] Meng B., Kemp S.A., Papa G., Datir R., Ferreira I.A., Marelli S., Harvey W.T., Lytras S., Mohamed A., Gallo G. (2021). Recurrent emergence of SARS-CoV-2 spike deletion H69/V70 and its role in the Alpha variant B.1.1.7. Cell Rep..

[64] Lan J., Ge J., Yu J., Shan S., Zhou H., Fan S., Zhang Q., Shi X., Wang Q., Zhang L. (2020). Structure of the SARS-CoV-2 spike receptor-binding domain bound to the ACE2 receptor. Nature.

[65] Greaney A.J., Loes A.N., Crawford K.H.D., Starr T.N., Malone K.D., Chu H.Y., Bloom J.D. (2021). Comprehensive mapping of mutations in the SARS-CoV-2 receptor-binding domain that affect recognition by polyclonal human plasma antibodies. Cell Host Microbe.

[66] Starr T.N., Greaney A.J., Hilton S.K., Ellis D., Crawford K.H.D., Dingens A.S., Navarro M.J., Bowen J.E., Tortorici M.A., Walls A.C. (2020). Deep mutational scanning of SARS-CoV-2 receptor-binding domain reveals constraints on folding and ACE2 binding. Cell.

[67] Yan R., Zhang Y., Li Y., Xia L., Guo Y., Zhou Q. (2020). Structural basis for the recognition of SARS-CoV-2 by full-length human ACE2. Science.

[68] Liu L., Iketani S., Guo Y., Chan J.F.-W., Wang M., Liu L., Luo Y., Chu H., Huang Y., Nair M.S. (2022). Striking antibody evasion manifested by the Omicron variant of SARS-CoV-2. Nature.

[69] Johnson B.A., Xie X., Bailey A.L., Kalveram B., Lokugamage K.G., Muruato A., Zou J., Zhang X., Juelich T., Smith J.K. (2021). Loss of furin cleavage site attenuates SARS-CoV-2 pathogenesis. Nature.

[70] Hoffmann M., Kleine-Weber H., Schroeder S., Krüger N., Herrler T., Erichsen S., Schiergens T.S., Herrler G., Wu N.-H., Nitsche A. (2020). SARS-CoV-2 cell entry depends on ACE2 and TMPRSS2 and is blocked by a clinically proven protease inhibitor. Cell.

[71] Bestle D., Heindl M.R., Limburg H., Van Lam Van T., Pilgram O., Moulton H., Stein D.A., Hardes K., Eickmann M., Dolnik O. (2020). TMPRSS2 and furin are both essential for proteolytic activation of SARS-CoV-2 in human airway cells. Life Sci. Alliance.

[72] Lubinski B., Fernandes M.H., Frazier L., Tang T., Daniel S., Diel D.G., Jaimes J.A., Whittaker G.R. (2022). Functional evaluation of the P681H mutation on the proteolytic activation of the SARS-CoV-2 variant B.1.1.7 (Alpha) spike. iScience.

[73] Peacock T.P., Goldhill D.H., Zhou J., Baillon L., Frise R., Swann O.C., Kugathasan R., Penn R., Brown J.C., Sanchez-David R.Y. (2021). The furin cleavage site in the SARS-CoV-2 spike protein is required for transmission in ferrets. Nat. Microbiol..

[74] Saito A., Irie T., Suzuki R., Maemura T., Nasser H., Uriu K., Kosugi Y., Shirakawa K., Sadamasu K., Kimura I. (2022). Enhanced fusogenicity and pathogenicity of SARS-CoV-2 Delta P681R mutation. Nature.

[75] Meng B., Abdullahi A., Ferreira I.A.T.M., Goonawardane N., Saito A., Kimura I., Yamasoba D., Gerber P.P., Fatihi S., Rathore S. (2022). Altered TMPRSS2 usage by SARS-CoV-2 Omicron impacts infectivity and fusogenicity. Nature.

[76] Watanabe Y., Bowden T.A., Wilson I.A., Crispin M. (2019). Exploitation of glycosylation in enveloped virus pathobiology. Biochim. Biophys. Acta Gen. Subj..

[77] Newby M.L., Fogarty C.A., Allen J.D., Butler J., Fadda E., Crispin M. (2023). Variations within the glycan shield of SARS-CoV-2 impact viral spike dynamics. J. Mol. Biol..

[78] Grant O.C., Montgomery D., Ito K., Woods R.J. (2020). Analysis of the SARS-CoV-2 spike protein glycan shield reveals implications for immune recognition. Sci. Rep..

[79] Casalino L., Gaieb Z., Goldsmith J.A., Hjorth C.K., Dommer A.C., Harbison A.M., Fogarty C.A., Barros E.P., Taylor B.C., McLellan J.S. (2020). Beyond shielding: The roles of glycans in the SARS-CoV-2 spike protein. ACS Cent. Sci..

[80] Ives C.M., Nguyen L., Fogarty C.A., Harbison A.M., Durocher Y., Klassen J.S., Fadda E. (2024). Role of N343 glycosylation on the SARS-CoV-2 S RBD structure and co-receptor binding across variants of concern. eLife.

[81] Lempp F.A., Soriaga L.B., Montiel-Ruiz M., Benigni F., Noack J., Park Y.-J., Bianchi S., Walls A.C., Bowen J.E., Zhou J. (2021). Lectins enhance SARS-CoV-2 infection and influence neutralizing antibodies. Nature.

[82] Shajahan A., Pepi L., Kumar B., Murray N., Azadi P. (2023). Site-specific N- and O-glycosylation mapping of the spike proteins of SARS-CoV-2 variants of concern. Sci. Rep..

[83] Wang D., Zhang Z., Baudys J., Haynes C., Osman S.H., Zhou B., Barr J.R., Gumbart J.C. (2024). Enhanced surface accessibility of SARS-CoV-2 Omicron spike protein due to an altered glycosylation profile. ACS Infect. Dis..

[84] Hodcroft E.B. (2021). CoVariants: SARS-CoV-2 Mutations and Variants of Interest. https://covariants.org/.

[85] Grantham R. (1974). Amino acid difference formula to help explain protein evolution. Science.

[86] Chou J.M., Tsai J.L., Hung J.N., Chen I.H., Chen S.T., Tsai M.H. (2022). The ORF8 protein of SARS-CoV-2 modulates the spike protein and its implications in viral transmission. Front. Microbiol..

[87] Kim I.J., Lee Y.H., Khalid M.M., Chen I.P., Zhang Y., Ott M., Verdin E. (2023). SARS-CoV-2 protein ORF8 limits expression levels of Spike antigen and facilitates immune evasion of infected host cells. J. Biol. Chem..

[88] Gangavarapu K., Latif A.A., Mullen J.L., Alkuzweny M., Hufbauer E., Tsueng G., Haag E., Zeller M., Aceves C.M., Zaiets K. (2023). Outbreak.info genomic reports: Scalable and dynamic surveillance of SARS-CoV-2 variants and mutations. Nat. Methods.

[89] Shu Y., McCauley J. (2017). GISAID: Global initiative on sharing all influenza data—From vision to reality. Euro Surveill..

[90] Elbe S., Buckland-Merrett G. (2017). Data, disease, and diplomacy: GISAID’s innovative contribution to global health. Glob. Chall..

[91] Cock P.J.A., Antao T., Chang J.T., Chapman B.A., Cox C.J., Dalke A., Friedberg I., Hamelryck T., Kauff F., Wilczynski B. (2009). Biopython: Freely available Python tools for computational molecular biology and bioinformatics. Bioinformatics.

[92] Pandas Development Team (2023). pandas-dev/pandas: Pandas (Version 2.0.0). *Zenodo*. https://zenodo.org/records/7794821.

[93] Marcais G., Delcher A.L., Phillippy A.M., Coston R., Salzberg S.L., Zimin A. (2018). MUMmer4: A fast and versatile genome alignment system. PLoS Comput. Biol..

[94] Katoh K., Standley D.M. (2013). MAFFT multiple sequence alignment software version 7: Improvements in performance and usability. Mol. Biol. Evol..

[95] Nei M., Gojobori T. (1986). Simple methods for estimating the numbers of synonymous and nonsynonymous nucleotide substitutions. Mol. Biol. Evol..

[96] Price M.N., Dehal P.S., Arkin A.P. (2010). FastTree 2—Approximately maximum-likelihood trees for large alignments. PLoS ONE.

[97] Virtanen P., Gommers R., Oliphant T.E., Haberland M., Reddy T., Cournapeau D., Burovski E., Peterson P., Weckesser W., Bright J. (2020). SciPy 1.0: Fundamental algorithms for scientific computing in Python. Nat. Methods.

[98] Harris C.R., Millman K.J., van der Walt S.J., Gommers R., Virtanen P., Cournapeau D., Wieser E., Taylor J., Berg S., Smith N.J. (2020). Array programming with NumPy. Nature.

[99] Hunter J.D. (2007). Matplotlib: A 2D graphics environment. Comput. Sci. Eng..

[100] Waskom M. (2021). seaborn: Statistical data visualization. J. Open Source Softw..

[101] Yi K., Kim S.Y., Bleazard T., Kim T., Youk J., Ju Y.S. (2021). Mutational spectrum of SARS-CoV-2 during the global pandemic. Exp. Mol. Med..

[102] Ravi V., Swaminathan A., Yadav S., Arya H., Pandey R. (2022). SARS-CoV-2 variants of concern and variations within their genome architecture: Does nucleotide distribution and mutation rate alter the functionality and evolution of the virus?. Viruses.

[103] Wagner C., Kistler K.E., Perchetti G.A., Baker N., Frisbie L.A., Torres L.M., Aragona F., Yun C., Figgins M., Greninger A.L. (2024). Positive selection underlies repeated knockout of ORF8 in SARS-CoV-2 evolution. Nat. Commun..

[104] Lee J.T., Yang Q., Gribenko A.V., Perrin B.S., Zhu Y., Cardin R., Liberator P.A., Anderson A.S., Hao L. (2022). Genetic surveillance of SARS-CoV-2 Mpro reveals high sequence and structural conservation prior to the introduction of protease inhibitor Paxlovid. mBio.

[105] Rashid F., Dzakah E.E., Wang H., Tang S. (2021). The ORF8 protein of SARS-CoV-2 induced endoplasmic reticulum stress and mediated immune evasion by antagonizing production of interferon beta. Virus Res..

[106] Zhang L., Lin D., Sun X., Curth U., Drosten C., Sauerhering L., Becker S., Rox K., Hilgenfeld R. (2020). Crystal structure of SARS-CoV-2 main protease provides a basis for design of improved α-ketoamide inhibitors. Science.

[107] Zhu Y., Yurgelonis I., Noell S., Yang Q., Guan S., Li Z., Hao L., Rothan H., Rai D.K., McMonagle P. (2024). In vitro selection and analysis of SARS-CoV-2 nirmatrelvir resistance mutations contributing to clinical virus resistance surveillance. Sci. Adv..

[108] Yaghi R.M., Wylie D.C., Andrews C.L., Dickert O.H., Ram A., Iverson B.L. (2024). An investigation of nirmatrelvir (Paxlovid) resistance in SARS-CoV-2 Mpro. ACS Bio Med. Chem. Au.

[109] Neilsen G., Lan S., Slack R.L., Lorson Z.C., Castaner A.E., Lee R., Edwards K.G., Zhang H., Lee J., Cantara W.A. (2025). Strategy to overcome a nirmatrelvir resistance mechanism in the SARS-CoV-2 nsp5 protease. Sci. Adv..

[110] Tchesnokova V., Kulasekara H., Larson L., Bowers V., Rechkina E., Kisiela D., Sledneva Y., Choudhury D., Maslova I., Deng K. (2021). Acquisition of the L452R mutation in the ACE2-binding interface of spike protein triggers recent massive expansion of SARS-CoV-2 variants. J. Clin. Microbiol..

[111] Cherian S., Potdar V., Jadhav S., Yadav P., Gupta N., Das M., Rakshit P., Singh S., Abraham P., Panda S. (2021). SARS-CoV-2 spike mutations, L452R, T478K, E484Q and P681R, in the second wave of COVID-19 in Maharashtra, India. Microorganisms.

[112] Cerutti G., Guo Y., Zhou T., Gorman J., Lee M., Rapp M., Reddem E.R., Yu J., Bahna F., Bimela J. (2021). Potent SARS-CoV-2 neutralizing antibodies directed against spike N-terminal domain target a single supersite. Cell Host Microbe.

[113] Dijokaite-Guraliuc A., Das R., Zhou D., Ginn H.M., Liu C., Duyvesteyn H.M., Huo J., Nutalai R., Supasa P., Selvaraj M. (2023). Rapid escape of new SARS-CoV-2 Omicron variants from BA.2-directed antibody responses. Cell Rep..

[114] Chen J., Wang R., Gilby N.B., Wei G.W. (2022). Omicron variant (B.1.1.529): Infectivity, vaccine breakthrough, and antibody resistance. J. Chem. Inf. Model..

[115] Balech B., Lo Presti A., Telegrafo C., Maisto L., Giombini E., Di Martino A., Ambrosio L., Tullo A., Stefanelli P. (2025). Investigating the evolutionary dynamics and mutational pattern of the SARS-CoV-2 spike gene on selected SARS-CoV-2 variants. PLoS ONE.

[116] Rogozin I.B., Saura A., Bykova A., Brover V., Yurchenko V. (2023). Deletions across the SARS-CoV-2 genome: Molecular mechanisms and putative functional consequences of deletions in accessory genes. Microorganisms.

[117] Akaishi T., Fujiwara K. (2023). Insertion and deletion mutations preserved in SARS-CoV-2 variants. Arch. Microbiol..

[118] Parsons R.J., Acharya P. (2023). Evolution of the SARS-CoV-2 Omicron spike. Cell Rep..

[119] Alisoltani A., Jaroszewski L., Iyer M., Iranzadeh A., Godzik A. (2022). Increased frequency of indels in hypervariable regions of SARS-CoV-2 proteins—A possible signature of adaptive selection. Front. Genet..

[120] Akaishi T., Fujiwara K., Ishii T. (2022). Insertion/deletion hotspots in the Nsp2, Nsp3, S1, and ORF8 genes of SARS-related coronaviruses. BMC Ecol. Evol..

[121] Mannar D., Saville J.W., Zhu X., Srivastava S.S., Berezuk A.M., Tuttle K.S., Marquez A.C., Sekirov I., Subramaniam S. (2022). SARS-CoV-2 Omicron variant: Antibody evasion and cryo-EM structure of spike protein–ACE2 complex. Science.

[122] Xue S., Han Y., Wu F., Wang Q. (2024). Mutations in the SARS-CoV-2 spike receptor-binding domain and their delicate balance between ACE2 affinity and antibody evasion. Protein Cell.

[123] Nguyen H.L., Nguyen T.Q., Li M.S. (2024). SARS-CoV-2 Omicron subvariants do not differ much in binding affinity to human ACE2: A molecular dynamics study. J. Phys. Chem. B.

[124] Starr T.N., Greaney A.J., Stewart C.M., Walls A.C., Hannon W.W., Veesler D., Bloom J.D. (2022). Deep mutational scans for ACE2 binding, RBD expression, and antibody escape in the SARS-CoV-2 Omicron BA.1 and BA.2 receptor-binding domains. PLoS Pathog..

[125] Xia S., Wang L., Zhu Y., Lu L., Jiang S. (2022). Origin, virological features, immune evasion and intervention of SARS-CoV-2 Omicron sublineages. Signal Transduct. Target. Ther..

[126] Wang Q., Iketani S., Li Z., Liu L., Guo Y., Huang Y., Bowen A.D., Liu M., Wang M., Yu J. (2023). Alarming antibody evasion properties of rising SARS-CoV-2 BQ and XBB subvariants. Cell.

[127] Gu H., Chen Q., Yang G., He L., Fan H., Deng Y.-Q., Wang Y., Teng Y., Zhao Z., Cui Y. (2020). Adaptation of SARS-CoV-2 in BALB/c mice for testing vaccine efficacy. Science.

[128] Mustafa Z., Kalbacher H., Burster T. (2022). Occurrence of a novel cleavage site for cathepsin G adjacent to the polybasic sequence within the proteolytically sensitive activation loop of the SARS-CoV-2 Omicron variant: The amino acid substitution N679K and P681H of the spike protein. PLoS ONE.

[129] Lubinski B., Frazier L.E., Phan M.V.T., Bugembe D.L., Cunningham J.L., Tang T., Daniel S., Cotten M., Jaimes J.A., Whittaker G.R. (2022). Spike protein cleavage-activation in the context of the SARS-CoV-2 P681R mutation: An analysis from its first appearance in lineage A.23.1 identified in Uganda. Microbiol. Spectr..

[130] Khatri R., Siddqui G., Sadhu S., Maithil V., Vishwakarma P., Lohiya B., Goswami A., Ahmed S., Awasthi A., Samal S. (2023). Intrinsic D614G and P681R/H mutations in SARS-CoV-2 VoCs Alpha, Delta, Omicron and viruses with D614G plus key signature mutations in spike protein alters fusogenicity and infectivity. Med. Microbiol. Immunol..

[131] Focosi D., Quiroga R., McConnell S., Johnson M.C., Casadevall A. (2023). Convergent evolution in SARS-CoV-2 spike creates a variant soup from which new COVID-19 waves emerge. Int. J. Mol. Sci..

[132] Lubinski B., Jaimes J.A., Whittaker G.R. (2022). Intrinsic furin-mediated cleavability of the spike S1/S2 site from SARS-CoV-2 variant B.1.1.529 (Omicron). bioRxiv.

[133] Aynaud M.M., Caldwell L., Al-Zahrani K.N., Barutcu S., Chan K., Obersterescu A., Ogunjimi A.A., Jin M., Zakoor K.-R., Patel S. (2024). Systematic surveillance of SARS-CoV-2 reveals dynamics of variant mutagenesis and transmission in a large urban population. Nat. Commun..

[134] Wang S., Ran W., Sun L., Fan Q., Zhao Y., Wang B., Yang J., He Y., Wu Y., Wang Y. (2024). Sequential glycosylations at the multibasic cleavage site of SARS-CoV-2 spike protein regulate viral activity. Nat. Commun..

[135] Huang H.Y., Liao H.Y., Chen X., Wang S.-W., Cheng C.-W., Mahmud S.A., Liu Y.-M., Mohapatra A., Chen T.-H., Lo J.M. (2022). Vaccination with SARS-CoV-2 spike protein lacking glycan shields elicits enhanced protective responses in animal models. Sci. Transl. Med..

[136] Zhang F., Schmidt F., Muecksch F., Wang Z., Gazumyan A., Nussenzweig M.C., Gaebler C., Caskey M., Hatziioannou T., Bieniasz P.D. (2024). SARS-CoV-2 spike glycosylation affects function and neutralization sensitivity. mBio.

[137] Liu P., Yue C., Meng B., Xiao T., Yang S., Liu S., Jian F., Zhu Q., Yu Y., Ren Y. (2024). Spike N354 glycosylation augments SARS-CoV-2 fitness for human adaptation through structural plasticity. Natl. Sci. Rev..

[138] Obermeyer F., Jankowiak M., Barkas N., Schaffner S.F., Pyle J.D., Yurkovetskiy L., Bosso M., Park D.J., Babadi M., MacInnis B.L. (2022). Analysis of 6.4 million SARS-CoV-2 genomes identifies mutations associated with fitness. Science.

[139] Bloom J.D., Neher R.A. (2023). Fitness effects of mutations to SARS-CoV-2 proteins. Virus Evol..

[140] Grifoni A., Weiskopf D., Ramirez S.I., Mateus J., Dan J.M., Moderbacher C.R., Rawlings S.A., Sutherland A., Premkumar L., Jadi R.S. (2020). Targets of T cell responses to SARS-CoV-2 coronavirus in humans with COVID-19 disease and unexposed individuals. Cell.

[141] Thoms M., Buschauer R., Ameismeier M., Koepke L., Denk T., Hirschenberger M., Kratzat H., Hayn M., Mackens-Kiani T., Cheng J. (2020). Structural basis for translational shutdown and immune evasion by the Nsp1 protein of SARS-CoV-2. Science.

[142] Takada K., Ueda M.T., Shichinohe S., Kida Y., Ono C., Matsuura Y., Watanabe T., Nakagawa S. (2023). Genomic diversity of SARS-CoV-2 can be accelerated by mutations in the nsp14 gene. iScience.

[143] Markov P.V., Ghafari M., Beer M., Lythgoe K., Simmonds P., Stilianakis N.I., Katzourakis A. (2023). The evolution of SARS-CoV-2. Nat. Rev. Microbiol..

[144] García-García T., Fernández-Rodríguez R., Redondo N., de Lucas-Rius A., Zaldívar-López S., López-Ayllón B.D., Suárez-Cárdenas J.M., Jiménez-Marín Á., Montoya M., Garrido J.J. (2022). Impairment of antiviral immune response and disruption of cellular functions by SARS-CoV-2 ORF7a and ORF7b. iScience.

[145] Guo K., Barrett B.S., Morrison J.H., Mickens K.L., Vladar E.K., Hasenkrug K.J., Poeschla E.M., Santiago M.L. (2022). Interferon resistance of emerging SARS-CoV-2 variants. Proc. Natl. Acad. Sci. USA.

[146] Liang F. (2023). Quantitative Mutation Analysis of Genes and Proteins of Major SARS-CoV-2 Variants of Concern and Interest. Viruses.

[147] Biswal M., Diggs S., Xu D., Khudaverdyan N., Lu J., Fang J., Blaha G., Hai R., Song J. (2021). Two conserved oligomer interfaces of NSP7 and NSP8 underpin the dynamic assembly of SARS-CoV-2 RdRP. Nucleic Acids Res..

[148] Schindewolf C., Menachery V.D. (2023). Coronavirus 2′-O-methyltransferase: A promising therapeutic target. Virus Res..

[149] Turakhia Y., Thornlow B., Hinrichs A., McBroome J., Ayala N., Ye C., Smith K., De Maio N., Haussler D., Lanfear R. (2022). Pandemic-scale phylogenomics reveals the SARS-CoV-2 recombination landscape. Nature.

